# Genetic encoding and mutagenesis of RNA droplet phenotypes

**DOI:** 10.1038/s44286-026-00454-4

**Published:** 2026-10-07

**Authors:** William Verstraeten, Mai P. Tran, Alena Taskina, Pranay Jaiswal, Ivar S. Haugerud, Claudia Helbig, Moritz Hamberger, Edward W. Green, Michael Platten, Christoph A. Weber, Kerstin Göpfrich

**Affiliations:** 1 Center for Molecular Biology of Heidelberg University (ZMBH), Heidelberg University, Heidelberg, Germany; 2 Max Planck School Matter to Life, Heidelberg, Germany; 3 Faculty of Mathematics, Natural Science, and Materials Engineering, Institute of Physics, University of Augsburg, Augsburg, Germany; 4 German Cancer Research Center (DKFZ) Heidelberg, CCU Neuroimmunology and Brain Tumor Immunology, Heidelberg, Germany; 5 German Cancer Consortium (DKTK), DKFZ, Core Center Heidelberg, Heidelberg, Germany; 6 Department of Neurology, Medical Faculty Mannheim, MCTN, University of Heidelberg, Mannheim, Germany; 7 Faculty of Bioscience, Heidelberg University, Heidelberg, Germany; 8 Helmholtz Institute for Translational Oncology Mainz, Mainz, Germany; 9 DKFZ-Hector Cancer Institute at the Medical Faculty Mannheim, Heidelberg University, Heidelberg, Germany; 10 Cluster of Excellence SynthImmune, Heidelberg University, Heidelberg, Germany

**Keywords:** RNA, Biological physics

## Abstract

RNA droplets assembled from cotranscriptionally folded nanostructures have recently emerged as a promising platform for constructing protocells and minimal synthetic cell models. A custom-designed DNA template encodes an RNA nanostar that self-assembles into droplets by kissing-loop interactions. Here we show that such RNA droplets encapsulate their DNA template, creating a genotype–phenotype link. We find that low mutation rates in the DNA template increase the phenotypical and functional complexity of the RNA droplets. Surprisingly, mutagenesis leads to the formation of long-lived vacuoles. With theory and next-generation sequencing, we explain this emergent behavior. We identify mutant nanostars that facilitate the formation of vacuoles through altered interactions between nanostars. Strikingly, vacuolated droplets exhibit micrometer-scale movement driven by internal vacuole dynamics – an emergent function resulting from mutagenesis. Beyond motility, we show that the vacuoles can be used as subcompartments to store cargo in the RNA droplets. Our findings establish RNA droplets as self-contained, evolvable systems, opening new avenues for the bottom-up evolution of synthetic cells and origins-of-life research.

**Figure:**
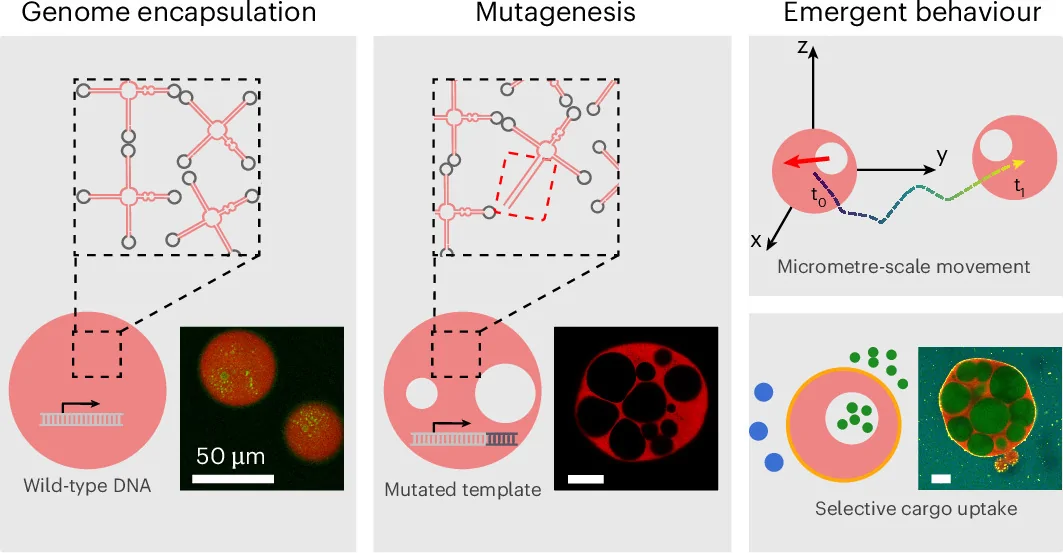

## Abstract (web summary)

This study shows that genetic mutations can generate cellular-like traits in RNA droplets. By encapsulating their DNA templates, the droplets establish a genotype–phenotype link, and low levels of mutagenesis give rise to stable vacuoles that enable cargo storage and micrometer-scale motion.

## Main

‘How simple can a cell be?’^1^. The answer depends on how we define life. At the transition from chemical to biological evolution, a minimal cell that can act as a precursor for life needs to self-replicate and evolve^2–4^. For evolution to occur, genetic material needs to undergo changes, and these changes must be reflected in the cell’s phenotype and functions which are subject to selection. Crucially, the genetic material must remain physically linked to the phenotype, for instance, in a compartment, to enable its own selection and inheritance^5–8^. Looking at today’s cells, it might seem obvious that a cell should be engineered from phospholipid membranes and proteins. However, it was estimated that such a cell would require a minimum of 150 genes for transcription and translation alone^9,10^. Lipid synthesis within vesicles, required for growth, is complex. Progress has been made^11,12^, but a sufficient rate for sustained growth and division cycles has not yet been achieved. Furthermore, the division of lipid-based compartments would have to be coupled to the replication and segregation of the information content inside^13^. A potential alternative is to bypass lipid membranes for compartmentalization and to use condensates instead. Condensates are liquid droplets that form through liquid–liquid phase separation. Unlike phospholipid vesicles, condensates can grow passively by fusion or spontaneous recruitment of additional monomers from the surrounding solution without the need for membrane-specific enzymes or complex synthetic pathways^11,12^. Division of condensates has also been achieved^14–17^, and theoretical work shows that simple active condensates^18^ can undergo sustained growth and division cycles^19,20^.

It is worth noting that most nucleic acid condensates assemble primarily through nonspecific electrostatic interactions between polycations and polyanions: accordingly, these droplets usually show limited sequence specificity^21,22^.

By contrast, condensates made purely from DNA nanostars are a promising model for synthetic cells because of their inherent integration of programmable information storage^14–16,23–28^. Recently, droplets made only from RNA nanostars that assemble through kissing-loop (KL) interactions have also been achieved^29,30^. These RNA droplets are produced cotranscriptionally from a DNA template. This system separates genotype (encoded in DNA) from phenotype (the physical traits of the RNA droplet), which is beneficial for its evolvability^4^. However, the evolvability of RNA droplets remains to be demonstrated. It is still unclear if they even fulfill the prerequisites for evolution, namely (1) encapsulation of the DNA template inside the compartment to establish a genotype–phenotype correlation, (2) mutations in the DNA affect the droplet phenotype, that is, support heritable phenotypic variation once genotype–phenotype coupling is in place, and (3) mutagenesis leads to functional phenotypic features and increased complexity (rather than only loss-of-function outcomes)^5,31–34^. Here, we first show that RNA droplets encapsulate their DNA template upon formation, thus fulfilling prerequisite (1). Taking advantage of this link between DNA code and its compartment, we study the effects of mutagenesis on the compartment’s architecture. Indeed, mutations in the DNA template lead to new droplet phenotypes (prerequisite (2)). In particular, we observe the formation of long-lived vacuoles—hence the emergence of a multicompartment system (prerequisite (3)). Unlike previously reported vacuolated condensates^35–37^, the vacuoles in these RNA droplets are stable for days. Using next-generation sequencing, we identify mutated DNA templates encoding mutant nanostars. We reverse-engineer the mutant nanostars and mix them at different ratios with the non-mutated variants to establish a phase diagram. By combining our experimental data with theory, we show that vacuoles form through a local instability of the RNA droplet driven by the RNA nanostar mutant that perturbs the interactions inside the droplet and facilitates the formation of vacuolated droplets. Interestingly, vacuolated RNA droplets exhibit micron-scale movement, leading to increased droplet fusion and growth, suggesting a functional consequence of mutation-induced changes in droplet architecture, fulfilling prerequisite (3). Moreover, we show that the vacuoles can be used as a new compartment for the recruitment of cargo within RNA droplets.

## Results

### Genotype–phenotype coupling and vacuole formation in RNA droplets

**Fig. 1:**
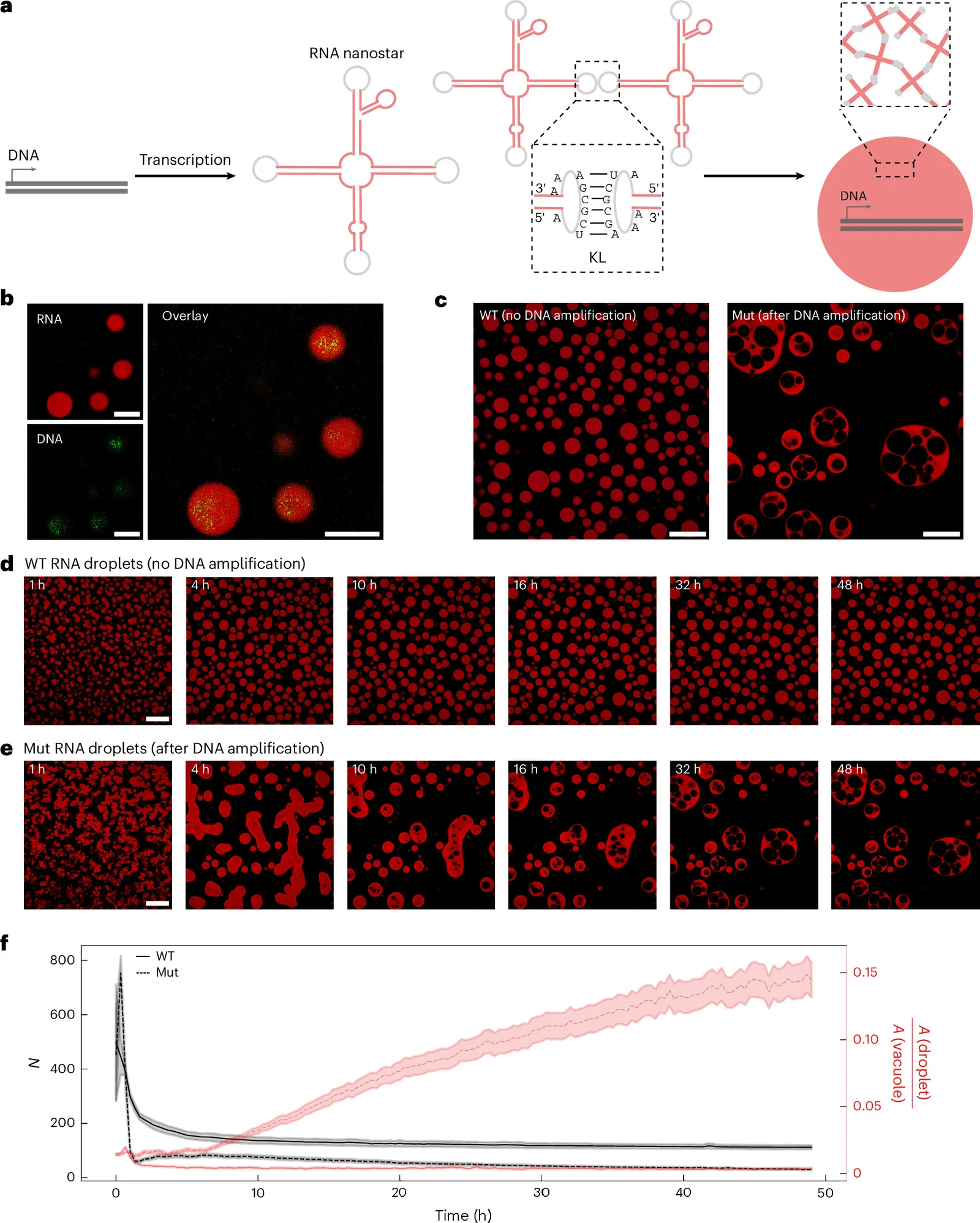
Genotype–phenotype coupling and vacuole formation in RNA droplets. **a**, Schematic representation of cotranscriptional folding of RNA nanostars from a DNA template and subsequent droplet formation by KL interactions between the nanostars. **b**, Confocal snapshots of RNA droplets (red) and their DNA template (green, labeled with fluorescein, *λ*_ex _= 488 nm). The overlay image shows that the RNA droplets encapsulate their DNA template. Scale bars, 50 μm. **c**, Confocal snapshots of RNA droplets (red) transcribed from nonamplified DNA WT (left) and amplified DNA Mut (right), respectively. Scale bars, 100 μm. **d**,**e**, Confocal snapshots showing WT (**d**) and Mut (**e**) RNA droplet formation over 48 h (red, droplets contain MGA and are labeled with malachite green dye, *λ*_ex_ = 640 nm). Top left corner: time refers to the time since the start of transcription. Scale bars, 100 μm. Experiments shown in **b**–**e** were performed twice independently with similar results. **f**, Quantification of the number of droplets *N* per field of view (left axis, black) and the ratio of vacuole-to-droplet area (*A*_vacuole_/*A*_droplet_) over time (right axis, red) for both WT (continuous line) and Mut (dashed line) droplets. The shaded area denotes the standard error of the mean for seven fields of view per condition (*n* = 7), across two independent replicates. Source data

First, we investigate whether RNA droplets encapsulate their DNA template, effectively linking the droplet phenotype and genotype. To this end, we use an RNA nanostar which folds cotranscriptionally from a single RNA strand (adapted from Fabrini et al.)^29^. The RNA nanostar consists of four arms, separated by single uracils. Each arm terminates with a self-complementary KL, which enables self-assembly of the nanostars and hence droplet formation^29,30,38^ (Fig. 1a). Two arms contain a 25 base pair (bp)-long RNA duplex stem. A third arm is functionalized with a malachite green aptamer (MGA) for fluorescence microscopy. We integrate a biotin aptamer into the fourth arm for binding of secondary molecular targets for further applications^39–41^ (Fig. 1a). In addition, we optimize the promoter and starting sequences from the original design^29^ to ensure optimal transcription yield (DNA template and primer sequences are shown in Supplementary Tables 1 and 2). To determine whether the DNA template is retained within the RNA droplets, we label the DNA template, dubbed wild-type (WT) DNA, using fluorescein-labeled dUTP (Supplementary Information section 1.1).

Confocal microscopy, shown in Fig. 1b, reveals that the DNA template is localized within the RNA droplets, suggesting that RNA droplets entrap their genetic material during the droplet formation process. The DNA is not homogeneously distributed within the RNA droplet. Instead, it forms DNA hotspots with diameters on the order of 1 μm to 3 μm, which may arise from sequence-specific RNA–RNA interactions together with electrostatic repulsion between DNA and RNA. A plausible mechanism is cotranscriptional capture: nascent nanostar RNA folds and engages in multivalent interactions while still tethered to the DNA template via the RNA polymerase, thereby promoting incorporation of the template during droplet assembly.

If the genotype, that is, the DNA template, is mutated, we could thus expect to see alterations in the droplet’s phenotype. Making use of the inherent self-complementarity of the DNA sequence, we perform polymerase chain reaction (PCR) to induce mutations in the WT DNA. The self-complementary sequences act as primers and generate a pool of mutated DNA genes (called Mut; Methods; Supplementary Figs. 1–2).

Interestingly, we find that transcription of Mut DNA consistently produces vacuolated RNA droplets (Mut droplets), whereas that of WT template produces only homogeneous spherical droplets without holes (WT droplets) (Fig. 1c, Supplementary Fig. 3).

We find that Mut droplets are significantly larger than WT droplets (Supplementary Fig. 4). Vacuoles appear in 74% of Mut droplets with a radius above 15 μm and in 94% with a radius more than 25 μm, while 0% of WT droplets exhibit vacuoles (Supplementary Fig. 5). Note that in DNA droplets, short-lived vacuoles (stable for <1 h) have been induced by DNase treatment^35^. Importantly, time-lapse imaging of WT droplets shows no vacuoles even after 48 h, while vacuoles remain stable, in Mut droplets, over days (Fig. 1d–f and Supplementary Videos 1 and 2). Long-lived vacuoles started to nucleate just after 2 h of transcription (Supplementary Video 2) and are distinctly visible from the 6-h time point onward (Fig. 1e and Supplementary Video 2). Before vacuole formation, regions of lower density emerge, which gradually shrink and coalesce into vacuoles. The vacuoles persist and grow over time (Supplementary Video 2). The quantification of the ratio of vacuole area to total droplet area reveals that the vacuoles continue to grow for 50 h (Fig. 1f). Moreover, we confirm that Mut droplets are more prone to fusion, resulting in fewer but larger droplets over time (Fig. 1f).

We find that vacuoles only rarely coalesce, indicating that internal rearrangements in the RNA-rich phase are strongly hindered. Brightfield imaging, which provides a larger depth of field than confocal fluorescence, reveals only a few vacuole–vacuole fusion events over 60 h (highlighted in Supplementary Videos 3 and 4). Consistent with this slow internal dynamics, shape relaxation after droplet–droplet fusion events proceeds on long timescales (capillary velocity ~10^−2^ μm s^−1^) and FRAP shows no recovery over 10 min (Supplementary Fig. 6), in line with previous reports for RNA condensates^29,30^. This suggests that the RNA-rich phase is highly viscous, which slows down vacuole coalescence.

While we observe that DNA clusters colocalize with the RNA-rich condensates, vacuole position does not seem spatially correlated with these clusters. As in confocal images, the DNA signal is not consistently enriched at vacuole interfaces (Supplementary Fig. 7).

Note that Fabrini et al. observed vacuoles in some RNA droplets and attributed this to enzymatic degradation by environmental nuclease activity or photodegradation^29^. To test whether nucleases are responsible for vacuole formation in our system, we perform additional control experiments using RNase inhibitors. The addition of RNase inhibitors has no effect on vacuole formation (Supplementary Fig. 8). Furthermore, WT droplets remain highly stable and do not show any vacuoles even after incubation for 13 days at 37 °C, whereas Mut droplets retain their vacuoles (Supplementary Fig. 9), suggesting that degradation of droplet material^17,35,37^ is not the underlying mechanism in our case.

It is known that perturbation of the dense liquid phase, by altering environmental conditions such as temperature or pH, can induce vacuolation in liquid droplets^36,42,43^. By contrast, in this study, environmental conditions are kept constant, with only RNA concentration gradually increasing during transcription, suggesting that the droplet phenotype is indeed a result of mutagenesis.

### Genetic encoding of vacuole formation

**Fig. 2:**
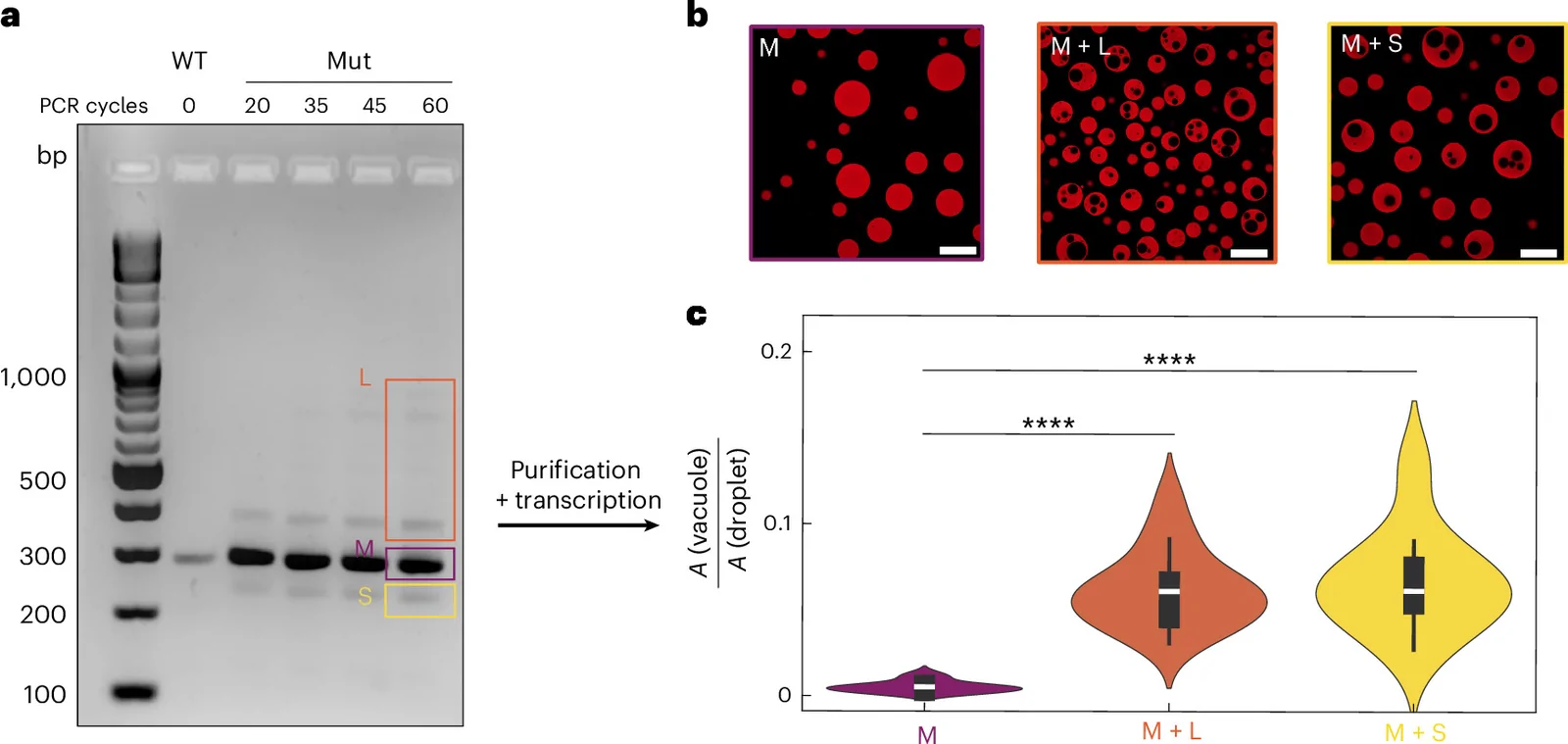
Nonspecific PCR products induce vacuole formation in RNA droplets. **a**, 3% agarose gel electrophoresis (AGE) of DNA before PCR (WT) (left) and of the PCR product (Mut) (right) from PCR with different number of cycles. Longer and shorter nonspecific PCR products are amplified. An increasing number of PCR cycles increases nonspecific product build-up. **b**, Confocal snapshots of RNA droplets (red) transcribed from gel-purified DNA bands: the main band (M) (left); a mixture of the main and higher bands (M+L) (middle); a mixture of the main and lower bands (M+S) (right). Droplets contain MGA and are labeled with malachite green dye, *λ*_ex _= 640 nm. Scale bars, 100 μm. Experiments were performed twice independently for each condition, with similar results. **c**, Quantification of the ratio of vacuole-to-total droplet area (*A*_vacuole_/*A*_droplet_), averaged per field of view, across two independent replicates. Statistics: median test (*n*_M_ = 18, *n*_M+L_ = 15, *n*_M+S_ = 10), significance with *P* values <0.001 are marked with ****. *P* = 4.3 × 10^−7^ (M versus M+L) and *P* = 4 × 10^−4^ (M versus M+S). Median is shown in white, box edges denote the lower and upper quartiles, and whiskers extend to the most extreme data points within 1.5× the interquartile range. Source data

Next, we investigate what causes vacuole formation in the Mut RNA droplets. An analysis of the WT DNA template by agarose gel electrophoresis (AGE) shows a single band before PCR as expected (Fig. 2a). After PCR, two distinct regions of DNA fragments above and below (L and S zone, respectively) the WT band (M zone) can be seen on the gel, confirming the presence of mutated DNA templates after PCR (Fig. 2a). Increasing the number of PCR cycles increases the build-up of these nonspecific PCR products. The higher bands (L), corresponding to longer DNA fragments, are probably caused by self-priming of the DNA template, whereas the lower bands (S) of shorter DNA can be explained by spurious mispriming events.

To test whether the nonspecific PCR products are responsible for vacuolation, we test the combination of the DNA from the three regions (L, M and S) for their ability to encode vacuolated droplets (Fig. 2 and Supplementary Fig. 10). As anticipated, transcription of the purified main band (M) produces normal RNA droplets without any vacuoles (Fig. 2b, left), comparable to those generated from the nonamplified WT DNA. Transcription of the purified longer DNA (L) with (M+L) or without the main band results in RNA droplets with vacuoles (Fig. 2b, middle, Fig. 2c and Supplementary Fig. 10). By contrast, RNA droplets from shorter DNA (S) only result in vacuolation in combination with the M band (M+S) (Fig. 2b, right, Fig. 2c and Supplementary Fig. 10). Taken together, these results indicate that nonspecific PCR products are indeed responsible for the vacuoles in the RNA droplets.

To confirm that self-priming of the DNA template is indeed causing the side products, we perform PCR in absence of all primers or of only the reverse primers. As expected, WT DNA is amplified in both cases, producing mainly nonspecific products (Supplementary Fig. 11). The resulting RNA droplets from PCR-amplified WT DNA without different sets of primers are vacuolated (Supplementary Fig. 11). In the absence of only the reverse primer, the resulting RNA droplets are membrane-like hollow structures; 100% of droplets with radii above 15 μm contain vacuoles (Supplementary Fig. 5).

Because we use a high-fidelity PCR kit to minimize substitution mutations, the nonspecific PCR products described above are mainly the results of deletion and duplication mutations. Nevertheless, it is highly interesting to make use of substitutions as a mutagenesis strategy as they are often used for in vitro protein engineering^44^.

### Vacuole formation by substitution mutagenesis

We thus set out to test substitution mutagenesis as a general route to increase the phenotypical complexity of RNA droplets and to probe how vacuolation depends on mutational load. Unlike the nonspecific PCR byproduct route used in Figs. 1 and 2, substitution-based mutagenesis produces a broad diversity of variants and is widely used in directed-evolution workflows because it is applicable to essentially any DNA sequence and offers quantitative control over mutation rate^45^.

For this, we use a nanostar-encoding DNA template, which can be amplified faithfully by PCR (Supplementary Fig. 1), termed WT2 (adapted from Fabrini et al.^29^; see Supplementary Table 1 for sequences).

Consistent with the WT design, the WT2 nanostar is composed of four arms terminating in a self-complementary KL. One arm contains the Broccoli aptamer (BrA) for fluorescent readout, the remaining three form 25-bp stems, and all arms are separated by single uracils.

**Fig. 3:**
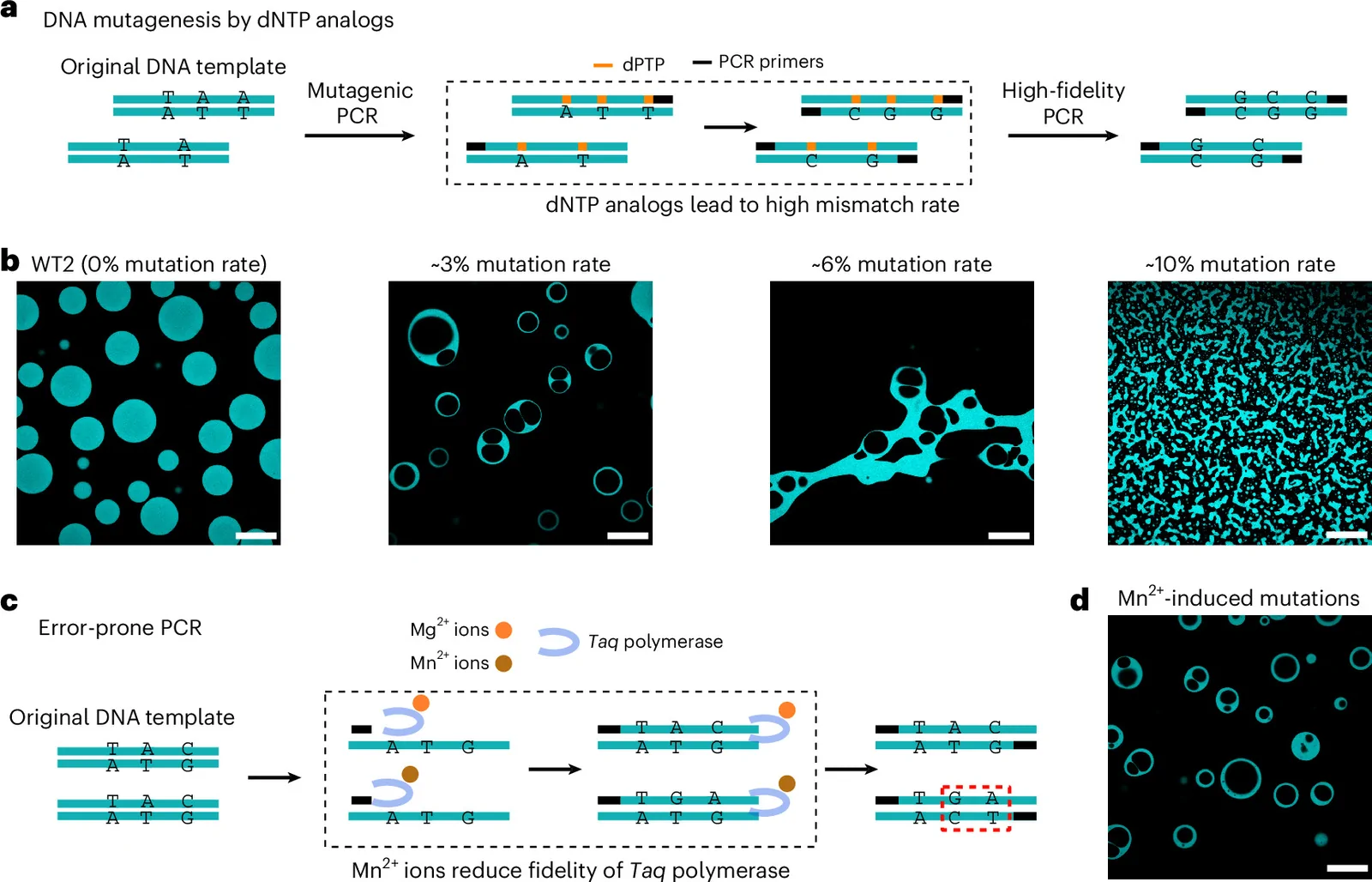
Substitution mutations in the DNA template lead to vacuolated droplets. **a**, Schematic illustration of mutagenesis induced by dNTP analogs. The mutagenesis rate is controlled by the number of PCR cycles with the dNTP analog, dPTP and followed by a high-fidelity PCR. **b**, Representative confocal snapshots of the RNA droplets (blue) transcribed from dPTP-induced mutated DNA templates after 48 h of transcription (corresponding mutation rates are annotated; Supplementary Fig. 12). **c**, Schematic illustration of mutagenesis by manganese ion-induced error-prone PCR. Manganese ions are used to destabilize *Taq* polymerase and reduce its fidelity. **d**, Representative confocal snapshot of RNA droplets (blue) transcribed from WT2 DNA mutated by error-prone PCR (five error-prone PCR cycles). RNA droplets contain a Broccoli aptamer (BrA) and are labeled with DFHBI-1T, *λ*_ex _= 488 nm. Scale bars, 100 μm. Experiments shown in **b** and **d** were performed twice independently with similar results.

We use dNTP analogs during PCR to selectively mutate the WT2 DNA template (Fig. 3a and Supplementary Fig. 12). This approach allows us to avoid mutations in the promoter region, necessary for transcription, while controlling the mutation rate by varying the number of PCR cycles^46^. We observe four distinct phenotypes, directly dependent on the mutagenesis rate (Fig. 3b and Supplementary Figs. 12 and 13). Remarkably, a low mutation rate of ~3% (corresponding on average to seven altered bases per RNA nanostar template) is sufficient to induce vacuole formation, while higher mutation rates result in a percolating RNA network or simply RNA aggregates (Fig. 3b).

As an orthogonal, widely used substitution mutagenesis method, we additionally use an error-prone PCR protocol to induce mutations by addition of manganese ions^45^. These ions reduce the ability of *Taq* polymerase to discriminate between nucleotides, thus reducing its fidelity during DNA replication^47^ (Fig. 3c). Again, RNA transcribed from the mutated DNA templates forms vacuolated droplets (Fig. 3d and Supplementary Figs. 12 and 13).

### Mechanism of vacuole formation

Although we have shown that genetic mutations can lead to vacuolation, it was observed as an emergent property, and we have yet to understand the underlying mechanism. To build an effective model for the Mut RNA droplets, we perform long-read nanopore DNA sequencing of the Mut DNA. We find that 94.7% of reads align with the WT sequence (Supplementary Fig. 14), in accordance with our observation that low mutagenesis rates are sufficient to induce vacuolation. The remaining 5.3% contain a T7 promoter and encode various nanostar-like structures, with at least one KL (Supplementary Fig. 14; see Supplementary Table 3 for sequences). The most common of these sequences (2.7%) encodes a nanostar of four arms with three functional KLs (and corresponds to the higher band in Fig. 2a). To test whether vacuolation depends on a single mutant sequence or instead on shared structural features, we design a small set of de novo mutants that reproduce the characteristic secondary-structure motif of this dominant mutant class while varying the number of functional KL interactions (1KL, 2KL and 3KL; Supplementary Fig. 15). When transcribed together with WT DNA, all three variants yield vacuolated condensates (Supplementary Fig. 15), confirming that vacuole formation is not restricted to unique mutant sequences.

**Fig. 4:**
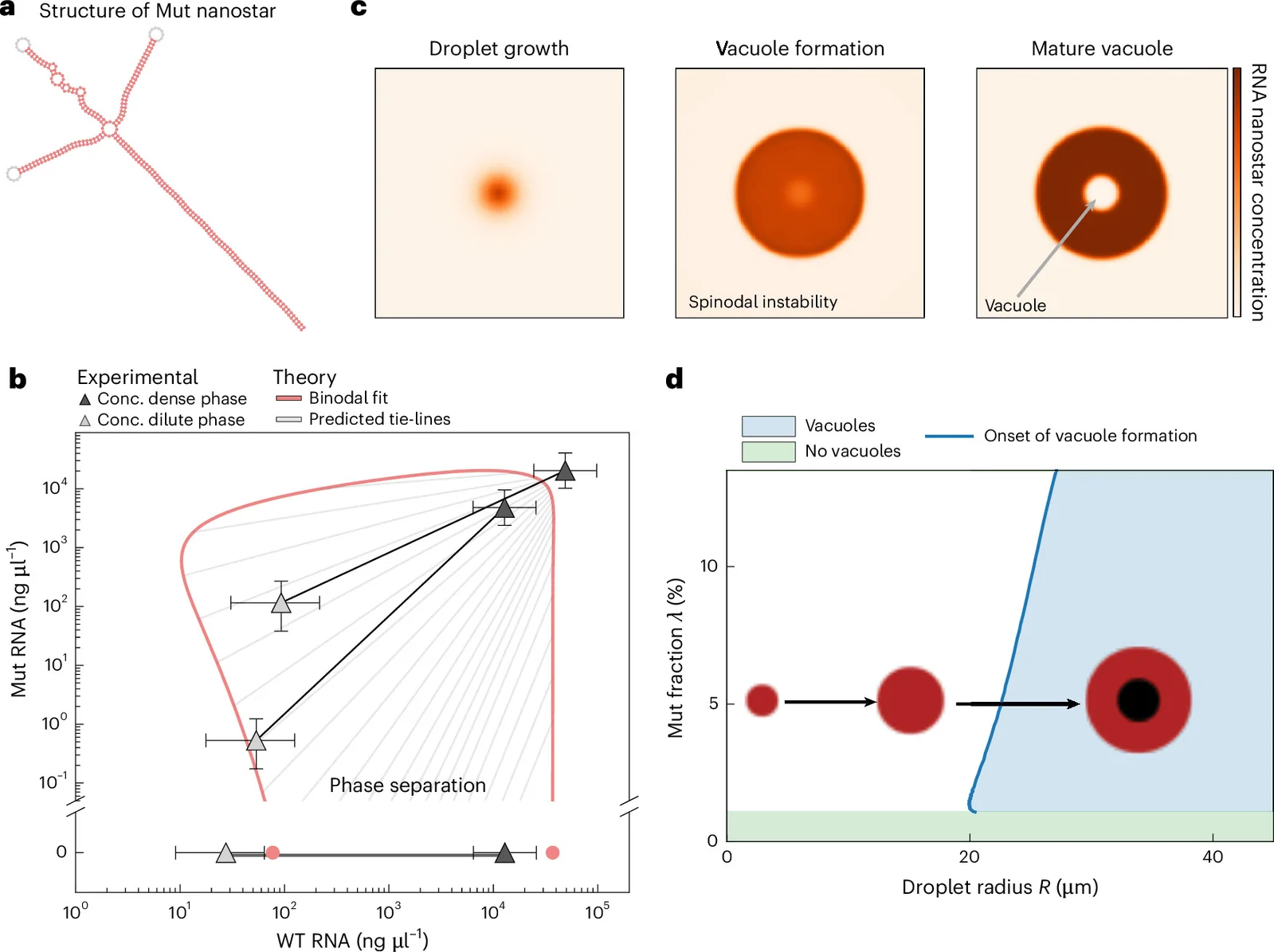
Mechanism of vacuole formation. **a**, Predicted folding of the Mut RNA. KLs are shown in gray. **b**, Thermodynamic phase diagram of nanostar phase separation. The diagram indicates the WT RNA to Mut RNA compositions for which phase separation into a nanostar-rich and poor phase occurs. Triangles are experimental data points determined from plate reader measurements (Supplementary Fig. 16 and Supplementary Table 4), showing the measured RNA concentrations (Conc.) in the dense and dilute phases, and black lines indicate the experimental tie lines. The binodal fit from the theory is shown in red, and the theoretical tie lines (light gray) agree well with the experimental tie lines (black). Data are presented as mean values ± standard deviation, *n* = 3 per condition, from two independent replicates. **c**, Schematic representation of the mechanism for vacuole formation in mutant droplets: transcription leads to droplet growth and gives rise to concentration gradients within the droplets by exchanging RNA nanostars with the nanostar-poor phase. Concentration gradients are more pronounced if diffusion inside is low. For large enough gradients, a spinodal instability is triggered locally, resulting in the formation of a vacuole. **d**, Diagram describing the onset of vacuole formation. Droplets have to be large enough to form vacuoles. According to the phase diagram shown in **b**, in the regime studied here, a large enough mutant DNA fraction *λ* is required to form the vacuole instability, which is consistent with the experimental observations. Source data

Still guided by these sequencing results, we then design a mutant nanostar (termed Mut RNA throughout this section) that reproduces the same secondary-structure motif as the most common mutant sequence (Fig. 4a and Supplementary Fig. 14) and carries a BrA to distinguish it from the WT RNA^48^.

Using this Mut RNA nanostar design, we experimentally determine the phase diagram for the WT and Mut nanostars on the basis of their mass concentrations to characterize their interactions (Fig. 4b).

We estimate the mass concentration of both WT and Mut RNA inside and outside the condensates by plate reader measurements of the respective phases (Fig. 4b and Supplementary Fig. 16; see Supplementary Information section 1.10 and Supplementary Table 4 for experimental details).

These mass concentrations provide approximate data points for a thermodynamic phase diagram spanned by WT and Mut RNA concentrations. Such a thermodynamic phase diagram is governed by the free energy of the system. Thus, to obtain the phase diagram for the experimental nanostar system, we fit the experimentally obtained data points using an effective free energy density *f*(*c*_WT_, *c*_Mut_), which depends on the mass concentrations of the WT RNA *c*_WT_ and the Mut RNA *c*_Mut_ (see equations (2) and (3) in Supplementary Information section 1.12). The interactions among the nanostars and with the solvent are described by three effective interaction parameters: *χ*_Mut,WT_, *χ*_WT,sol_ and *χ*_Mut,sol_, where sol indicates the solvent. From the fits, we obtain these three parameters together with the molecular volume of the WT RNA and Mut RNA. The theoretical phase diagram is obtained from the free energy density *f*(*c*_WT_, *c*_Mut_) by solving the conditions for phase equilibrium (see Supplementary Information section 1.12 and Supplementary Fig. 17 for details).

Figure 4b shows the fit of the phase diagram to the experimentally measured mass concentrations. In particular, the tie lines connecting the coexisting phases (thin gray lines) are captured well by the theory. Without Mut RNA, the WT RNA-rich phase has a roughly 400-fold higher mass concentration than the RNA-poor phase. Upon adding Mut RNA, the phase separation propensity remains roughly constant, which is indicated by an almost vertical slope of the RNA-poor branch of the phase diagram (Fig. 4b, left-most branch of the red binodal line). From the fit, we find that the molecular volume of mutant RNA nanostars slightly exceeds the one of the WT, which is consistent with the Mut nanostar having a slightly longer arm compared with the WT (Fig. 4a). Moreover, we find that nanostar phase separation is driven by adhesive interactions among the nanostars, while interactions with the solvent are negligible. Finally, the Mut–Mut interactions turn out to be less adhesive than the WT–WT interactions (see Supplementary Table 5 for values), which is consistent with the design of both RNA nanostars. In summary, the ternary effective free energy can reproduce the experimentally observed phase-separating behavior, yielding physically consistent parameters and highlighting how the interactions between WT and Mut RNA regulate phase separation.

Having determined the phase diagram, we can now understand the mechanism behind the vacuole formation (Fig. 4c). We begin the explanation when no nanostars have yet been transcribed and the system is homogeneous. When the T7 RNA polymerase starts transcribing the two types of nanostars, the fraction of Mut to WT nanostars *c*_Mut-RNA_(*t*)/*c*_WT-RNA_(*t*) is fixed by the fraction of mutant DNA, *λ* = *c*_Mut-DNA_/*c*_WT-DNA_. When transcription pushes the system beyond the saturation concentrations by crossing the dilute phase branch of the phase diagram (Fig. 4b), RNA nanostar-rich droplets form. Transcription drives droplet growth with a growth speed that is set by the transcription rate *k* and by thermodynamic properties such as interaction parameters and molecular volumes that are determined by the phase diagram. When droplets grow and concentrations outside change due to transcription, the droplet composition at the interface changes when the line described by *λ* deviates from the phase diagram’s tie line (Supplementary Fig. 18). This change in composition gives rise to concentration gradients inside when it is faster than nanostar diffusion inside droplets^29,49^ (Supplementary Video 5).

Such concentration gradients were indeed experimentally observed at the onset of vacuole formation (Fig. 1e, darker regions at 4-h time point). When these gradients get strong enough such that the composition crosses the spinodal line locally in the phase diagram, a spinodal instability occurs inside the droplet, leading to a new interface inside and thus a vacuole forms.

To test this mechanism, we derived a kinetic model for phase separation with transcription relying on the parameters obtained from the phase diagram (see Supplementary Information section 1.13 for details on the theoretical model). We obtain transcription rates (see Supplementary Table 6 for values) from transcription experiments and fitting the measured RNA concentration dynamics to the kinetic model (see Supplementary Information section 2.1 and Supplementary Fig. 19 for details) and we use literature-derived estimates of diffusion coefficients^50^. We apply the kinetic model (see Supplementary Table 7 for values) to the onset of vacuole formation and calculate the radius above which the spinodal instability occurs, eventually leading to vacuoles. We find that a vacuole cannot form when droplets are too small as diffusion masks concentration gradients inside the droplets. Consistent with the experimental observations, the model finds that vacuoles form for droplets with radii above ~21.5 μm (Fig. 4d and Supplementary Fig. 20). Moreover, we unravel the role of the mutant for vacuole formation inside the nanostar-rich droplets. According to our model, vacuoles only form when the mutant fraction *λ* ≳ 2% (Fig. 4d). Consistent with this, cotranscription at *λ* = 11% results in vacuoles for droplets with radii above ~24 μm (Supplementary Fig. 21). According to our model, the threshold value of *λ* results from the inside diffusivity of the Mut nanostar exceeding that of the WT, favoring the nanostar composition crossing the spinodal in the phase diagram. In summary, transcription of nanostars generates pronounced composition gradients of WT and Mut nanostars within the droplets owing to the low diffusion of RNA nanostars, triggering the formation of vacuoles via a local spinodal instability.

We also hypothesized that Mut RNA could stabilize vacuoles by acting as a surfactant, as proposed for hollow condensates in other systems^51^. The imaging of the annealed WT and Mut nanostars in water-in-oil droplets shows no enrichment of Mut at the water-condensate interface (Supplementary Fig. 22), suggesting that the Mut RNA does not behave as a surfactant under our conditions, leading us to discard the surfactant hypothesis. The long lifetime of vacuoles is probably explained by slow internal dynamics of the highly viscous RNA-rich phase (Supplementary Fig. 6), such that vacuole–vacuole fusion events remain rare on experimental timescales (Supplementary Videos 3 and 4).

### Gain of synthetic cell function through mutagenesis

A key requirement of synthetic cell models is that genotypic changes translate into functional phenotypic outcomes that can be selected for.

**Fig. 5:**
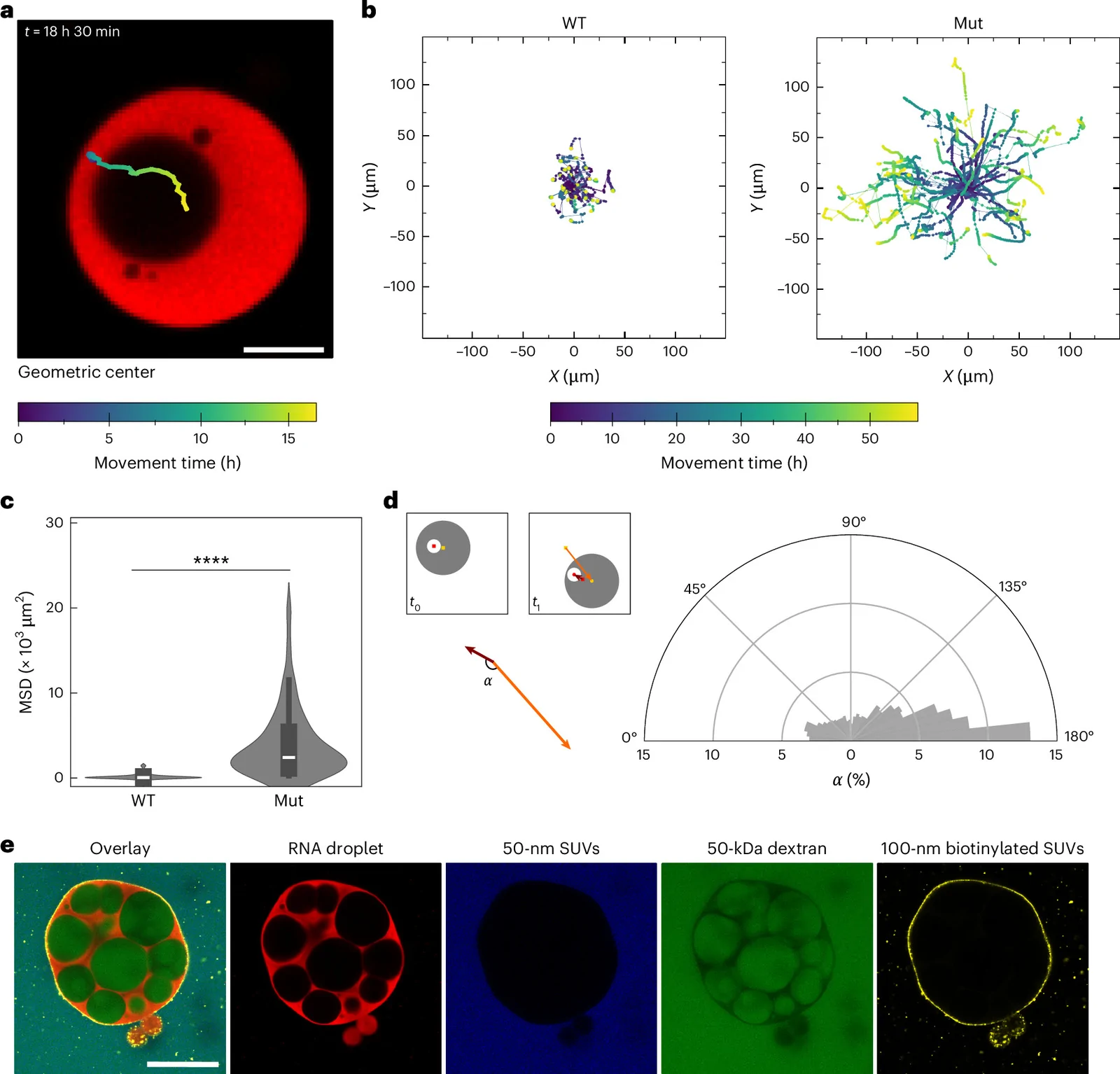
Gain of synthetic cell function through mutagenesis. **a**, Micrometer-scale movement of vacuolated RNA droplets. Representative confocal image and trajectory of a moving Mut RNA droplet (red) over time. Scale bar, 20 μm. **b**, Medusa plots of the movement of individual RNA droplets for WT (left) and Mut droplets (right), only the 50 most mobile droplets are shown for both RNA species for clarity. **c**, Quantification of the MSD for the 100 most mobile WT and Mut RNA droplets. Droplets were tracked for up to 56 h. Only the top 100 most mobile droplets are taken into account, across two independent replicates from *n* = 1,489 WT and *n* = 724 Mut tracked trajectories. Statistics: median test, significance with *P* values <0.001 are marked with **** (*P* = 2.8 × 10^−33^). Median is shown in white, box edges denote the lower and upper quartiles, and whiskers extend to the most extreme data points within 1.5× the interquartile range. **d**, Polar plot showing distribution of the angle *α*, defined as the angle between the droplet’s geometric center displacement vector (orange) and the displacement vector of the vacuole centers’ relative to the droplet’s geometric center (red, *n*_angles_ = 2,459). Data shown in **a**–**d** were acquired from two independent replicates. **e**, Confocal overlay showing one vacuolated Mut RNA droplet from a single experiment (red, droplet contains MGA and is labeled with malachite green dye *λ*_ex _= 640 nm), excluding DOPC SUVs (blue, 50-nm diameter, labeled with ATTO-390 PE, *λ*_ex _= 405 nm), uptake of 50-kDa FITC-dextran (green, *λ*_ex _= 488 nm) and selectively recruiting biotinylated SUVs (yellow, 100-nm diameter, labeled with Liss Rhod PE *λ*_ex_ = 561 nm). Scale bar, 100 μm. Source data

We find that mutations of WT to Mut RNA droplets lead not only to vacuole formation, but also to emergent functional behavior, namely droplet movement (Supplementary Videos 1 and 2). To quantify this behavior, we track the geometric center of individual WT and Mut droplets over time from confocal time-lapse microscopy (Fig. 5a,b, Supplementary Fig. 23 and Supplementary Video 6). The droplet trajectories are shown in a medusa plot for both RNA species in Fig. 5b (only 50 highest trajectories are shown in each condition for clarity). These plots reveal that Mut droplets travel further than WT droplets and exhibit irregular, burst-like movement characterized by sudden displacements (Supplementary Fig. 24). Motion persists for more than 50 h. We then calculate the mean squared displacement (MSD) of the 100 most mobile droplets for both WT and Mut droplets. Mut droplets show significantly enhanced motility compared with WT droplets (*P* < 0.001, Fig. 5c). This enhanced motility leads to an increase in fusion events, as moving droplets have a higher probability of encountering other droplets. As a result, Mut droplets are larger (Supplementary Fig. 4) and show a reduction in droplet number, consistent with our previous observations in Fig. 1f.

We hypothesize that droplet motion is driven by the internal dynamics of vacuole formation: as vacuoles nucleate and grow, they displace RNA, and because the vacuole-to-droplet volume ratio can be high, this redistribution shifts the droplet’s center of mass. To verify that vacuoles are causing droplet motion, we measure the angle *α* between the displacement of the vacuole center and that of the droplet geometric center (Fig. 5d; see Supplementary Information section 1.6 for details). On the basis of our hypothesis, we expect angle *α* > 90°, as this would indicate that the vacuole and droplet center move in opposite directions (Fig. 5d). Indeed, the histogram of *α* shows that the most frequent angles fall between 174° and 180° (Fig. 5d and Supplementary Figs. 23–25), suggesting that mass displacement by the vacuoles underlies Mut droplet motion. We frequently observe droplet fusion, followed by the formation of new vacuoles, which results in further movement (Fig. 5b and Supplementary Video 2). Importantly, this mechanism is distinct from other previously reported moving DNA droplets^35,52,53^: in our case, vacuoles remain stable throughout the trajectory and movement can occur repeatedly. Because vacuoles form at essentially random locations, there is no persistent polarity or propulsion axis as seen in self-propelled droplets^54^.

Beyond motility, we next want to explore if the vacuoles can serve as internal compartments and thus endow the mutated droplets with yet another functional advantage. The vacuoles establish an internal dilute phase within the droplets. To illustrate the functional consequences of the emergence of subcompartments, we study cargo recruitment to the RNA-dense phase as well as to the vacuoles (Fig. 5e). First, uptake into the droplets should be size selective.

We find that the dense RNA matrix excludes large nanoparticles such as 50-nm small unilamellar lipid vesicles (SUVs, blue), whereas smaller molecules such as 50-kDa dextran (green, ~10 nm)^55^ can diffuse through the RNA-dense phase into the vacuoles. Confocal imaging clearly shows that dextran is only present in the RNA dilute phase. A droplet without vacuoles would thus not have the capability to uptake and store this type of cargo. To demonstrate the sequence-specific addressability of the RNA droplets, we additionally make use of the biotin aptamer encoded in the RNA nanostar to specifically recruit biotinylated SUVs to the droplet interface, forming a shell-like structure around the condensate (Fig. 5e, right-most image). Together, these experiments demonstrate that vacuolated RNA droplets can be turned into complex cargo-loaded multicompartment assemblies.

## Discussion

Mutagenesis has not only led to the first primitive cells at the origins of life but also is the basis for the phenotypical and functional diversity of today’s life forms. Evolution of synthetic cells is thus one of the main goals of bottom-up synthetic biology. An evolvable system requires linking genotype to phenotype in a way that allows heritable variation and selection. In this study, we demonstrate that RNA droplets formed by cotranscriptional folding of RNA nanostars encapsulate their DNA template, thereby establishing a physical and functional link between genetic code and compartment phenotype. Furthermore, mutations in the DNA template reliably induce distinct phenotypic changes in the droplet architecture.

We find that PCR-induced mutations, either through nonspecific amplification or through targeted substitution mutageneses, result in dramatic changes to droplet morphology, most notably the formation of long-lived vacuoles within RNA droplets. Unlike previous reports of hollow condensates, we show that, rather than resulting from environmental perturbation^35,36,42,43^, the vacuolation is encoded in the genes encapsulated inside the RNA droplet.

With next-generation nanopore sequencing, we identify the DNA sequences which are responsible for the vacuolation in Mut droplets. On the basis of these sequences, we reverse-engineer a model mutant nanostar, which allows us to experimentally determine a phase diagram.

We theoretically describe the free energy density as a function of the mutant fraction and find that vacuoles arise from a local instability of the RNA droplet triggered by transcription of the RNA nanostar mutant, which perturbs intradroplet interactions. This demonstrates how mutations on a genetic level can modulate the thermodynamics of RNA droplets. Remarkably, the emergence of vacuoles is not merely a structural change. We show that vacuolated droplets exhibit micrometer-scale movement caused by internal mass redistribution during vacuole formation.

Such mutation-induced motility represents an emergent functional phenotype and highlights the capacity of RNA droplets to translate genetic variation into selectable functional traits and a step toward open-endedness in evolution. We show that the function of vacuoles in RNA droplets goes beyond movement and that they can be used in parallel with aptamers to recruit and contain different cargoes depending on their size and chemical properties.

The RNA droplets satisfy multiple prerequisites for Darwinian evolution: retention of genetic material, mutation-driven phenotypic variability, and the emergence of selectable functional traits. One such trait could be the selection for more hollow RNA droplets, toward evolving a vesicle-like RNA condensate. Evolvability is crucial toward the creation of synthetic life, where Darwinian evolution—leading to open-ended evolution—is a key characteristic. Evolution will also be a clear feat for biotechnological applications where structure–function relationships of increasingly complex systems become obscure and hard to engineer^4^. We anticipate that RNA droplets could be evolved for more complex desirable features such as distinct material properties, self-propelled microswimmers and drug delivery systems. Sequence programmable function can be added by including RNA aptamers to recruit specific molecules^29,30,56^ for computational purposes^57^ or by using the inherent catalytic abilities of ribozymes^58–60^. Further studies exploiting RNA droplets as synthetic cells could look into integrating ribozymes and aptamers into the RNA droplets to encode dynamic behavior of synthetic cells as a starting point for mutagenesis and evolution. Ultimately, RNA droplets and their mutability may contribute to our understanding of how life could have emerged from nonliving molecular assemblies in an RNA world, inform the design of synthetic life and open up opportunities for evolvable materials in biomedicine, biotechnology and beyond.

## Methods

### Materials

All reagents were ordered from Sigma-Aldrich and dissolved in RNase-free water unless stated otherwise.

### High-fidelity PCRs

DNA templates and primers were ordered from Integrated DNA Technologies, with templates provided as gBlocks and primers as oligos (purification: standard desalting). The initial sequences for the formation of RNA droplets were adapted from G. Fabrini et al.^29^ with modifications in the promoter and some of the KLs. All sequences can be found in Supplementary Tables 1 and 2 in the Supplementary Information and are additionally provided in machine-readable form as Supplementary Data 1. DNA templates were amplified by PCR using Phusion High-Fidelity DNA Polymerase in HF-buffer from New England Biolabs (NEB) (cat. no. M0530S) with an annealing temperature of 62 °C for 20 cycles, at a starting template concentration of 0.5 ng μl^−1^. For the vacuolated droplets in Figs. 1, 2 and 5, the DNA templates were amplified for 45 cycles instead. The resulting PCR products were systematically purified using a Qiagen PCR purification kit (cat. no. 28104) and analyzed by 3% native agarose gel electrophoresis (100-V run for 2 h in 1× TAE buffer). For the purified DNA sequences shown in Fig. 2, the QIAquick Gel Extraction Kit (cat. no. 28706) was used, according to the manufacturer’s instructions.

### Mutagenic PCR

For the mutagenic PCR with dNTP analogs, we used the Jena Bioscience dNTP-Mutagenesis Kit (cat. no. PP-101); 2 μl dPTP analog was added to the PCR mix to induce mismatches. Following the mutagenic PCR, a normal high-fidelity PCR was performed for 20 cycles. For the error-prone PCR, we used the normal *Taq* PCR Kit from NEB with the Standard *Taq* (Mg-free) Reaction Buffer Pack with 7 mM MgCl_2_ and 0.5 mM MnCl_2_ for 5 PCR cycles. Following purification, all DNA samples were stored at −20 °C until further use.

### RNA droplet transcription

Transcription was carried out in a homemade transcription buffer (100 mM HEPES, 20 mM Mg(CH_3_COO)_2_, 10 mM rNTPs (2.5 mM each), 1.5 mM DTT, 1.5 mM spermidine, 66.6 mM sucrose) and a 1:10 volumetric dilution of the T7 RNA Polymerase Mix (cat. no. B9015SVIAL) from NEB and final DNA concentration of 3ng μl^−1^. For droplets shown in red, 50 μM malachite green (cat. no. 38978) was added to the reaction mixture, for droplets shown in blue, 50 μM of DFHBI-1T (cat. no. SML2697-5MG) was used. In confocal time-lapse experiments, malachite green concentration was increased to 100 μM to compensate for photobleaching. RNA polymerase was always added to the reaction mix as a last step. After mixing 40 μl of the mixed sample was added to the microscopy observation chamber and stored at 37 °C.

### Observation chamber preparation

A microscopy cover slide attached to a six-channel Ibidi sticky slide VI 0.4 (cat. no. 80601) was passivated with 5% (W/V) polyvinyl alcohol (cat. no. 363170) rinsed with nuclease-free water and dried under nitrogen flow. Subsequently, the prepared sample was added to the channels of the Ibidi slide and the channels were immediately sealed using a two-component glue (Picodent cat. no. 1300 1000) to prevent evaporation. Slides were always prepared right before the imaging experiment.

### Confocal imaging

Before imaging, the incubation chamber of the confocal microscope was heated up to 37 °C for at least 30 min. The samples were visualized using a confocal laser scanning microscope (LSM 900, Carl Zeiss AG). A 20× objective (Plan-Apochromat 20×/0.8 M27, Carl Zeiss AG) was employed for image acquisition, and the internal magnification was set to 0.5×. To prevent any bias and capture RNA droplets of all sizes, images were consistently taken at different z-planes.

Images were acquired using the ZEN software (ZEN Blue, version 3.1; Carl Zeiss AG). Confocal micrographs shown in the figures of the main text were processed in Fiji (ImageJ2, Version: 2.3.0/1.54f). The brightness and contrast were adjusted linearly, and adjustments were always applied uniformly across the entire image; no local or region-specific adjustments were made.

### Statistics and Reproducibility

Images used for endpoint quantification (including droplet area statistics) were acquired roughly 48 h after the start of transcription. The time-lapse datasets shown in Fig. 1d,e and quantified in Fig. 1f are exceptions; for these datasets, the indicated time points represent the elapsed time since the start of transcription. As previous reports showed the induction of vacuoles in coacervates due to changing environmental conditions and/or nucleases, all slides contained at least one negative control (WT RNA droplets). To prevent bias, images were analyzed with a custom-written Python script, available on the group GitHub at https://github.com/Biophysical-Engineering-Group/RNAdroplets.

### Use of generative AI

During the preparation of this work, the authors used ChatGPT4-5 and Claude’s model Opus 5 to help with code generation and writing. After using this tool or service, the authors reviewed and edited the content as needed and take full responsibility for the content of the publication.

### Reporting summary

Further information on research design is available in the Nature Portfolio Reporting Summary linked to this article.

## Supplementary information

Supplementary Information Supplementary Figs. 1–25, materials and methods, Tables 1–7 and Video Descriptions 1–6. Additional notes on estimates of kinetic parameters for the theoretical model.

Reporting Summary

Peer Review File

Supplementary Data 1 DNA sequences in Excel format.

Supplementary Video 1 WT droplet formation.

Supplementary Video 2 Mut droplet formation.

Supplementary Video 3 Fusion of vacuoles.

Supplementary Video 4 Additional fusion of vacuoles.

Supplementary Video 5 Theory predicted dynamics.

Supplementary Video 6 Moving Mut droplet.

## Source data

Source Data Fig. 1 Data used for plotting graphs in Fig. 1.

Source Data Fig. 2 Data used for plotting graphs in Fig. 2 and uncropped gel shown in Fig. 2a.

Source Data Fig. 4 Data used for plotting graphs in Fig. 4.

Source Data Fig. 5 Data used for plotting graphs in Fig. 5.

## Data Availability

Source data are provided with this paper. Source data used in the Supplementary Information and other source data is available upon reasonable request to the corresponding authors.

## Code availability

All custom code used for image analysis, droplet segmentation and plotting and for the generation of the theory is publicly available via GitHub at https://github.com/Biophysical-Engineering-Group/RNAdroplets. The code used for the theory and numerical analysis is available via Zenodo at https://doi.org/10.5281/zenodo.21979218 (ref. ^61^).
