# Supplementary material for: Genetic encoding and mutagenesis of RNA droplet phenotypes
Source: Nat Chem Eng. 2026 Oct 7;3(10):573–83. doi: 10.1038/s44286-026-00454-4 (PMC13680278; doi:10.1038/s44286-026-00454-4)
Supplement: Supplementary file 1 [file 44286_2026_454_MOESM1_ESM.pdf]

nature chemical engineering

Supplementary information

https://doi.org/10.1038/s44286-026-00454-4

Genetic encoding and mutagenesis of RNA droplet phenotypes

In the format provided by the authors and unedited

Contents

1 Supplementary Materials & Methods 2
1.1 Fluorescent labelling of DNA templates . . . 2
1.2 Image analysis: quantification of droplet/vacuoles area and size . . . 3
1.3 Image analysis: line profile analysis . . . 3
1.4 FRAP measurements . . . 3
1.5 Tracking movement of vacuolated RNA droplets . . . 3
1.6 Tracking of vacuoles and movement direction analysis . . . 4
1.7 Droplet fusion and relaxation analysis . . . 5
1.8 DNA sequencing and analysis . . . 5
1.9 Annealing of RNA condensates in water-in-oil droplets . . . 6
1.10 Experimental phase diagram . . . 6
1.11 Transcription rate measurement on plate reader . . . 7
1.12 Thermodynamic model for the phase diagram . . . 7
1.13 Theoretical model for the onset of vacuole formation . . . 9
1.13.1 Transcription reaction, model parameters and constraints . . . 9
1.13.2 Kinetics in RNA-nanostar poor phase . . . 10
1.13.3 Droplet growth: Kinetics of droplet interface . . . 12
1.13.4 Kinetics in RNA-nanostar rich droplet phase . . . 13
1.13.5 Physical mechanism of vacuole formation . . . 14
1.14 Estimate of droplet volume fraction in the reaction . . . 15

2 Estimates of kinetic parameters 16
2.1 Transcription rate coefficient . . . 16
2.2 Time when the nutrient rNTP gets depleted . . . 18
2.3 Diffusivity inside . . . 18
2.4 Viscosity inside . . . 19
2.5 Capillary velocity, viscosity and surface tension . . . 19

3 Supplementary Tables 20
3.1 DNA sequences . . . 20
3.2 Phase separation experiments and model calibration . . . 23

4 Supplementary Figures 26
4.1 Agarose gel electrophoresis (AGE) of WT DNA and WT2 DNA before and after PCR amplification . . . 26
4.2 Mechanism for non-specific PCR product formation . . . 27
4.3 Confocal z-stack of WT RNA droplets . . . 28
4.4 Violin plots of the droplet area for Mut and WT droplets . . . 29
4.5 Percentage of vacuolated droplets from Mut DNA, WT DNA and PCR-amplified WT DNA without reverse primers . . . 30
4.6 Quantification of the material properties of RNA droplets . . . 31
4.7 Co-localisation of DNA templates with RNA droplets . . . 33
4.8 Transcription of Mut DNA with RNase inhibitors . . . 34

4.9 Mut and WT RNA droplets after 13 days of incubation at $37\,^{\circ}\mathrm{C}$ 35
4.10 RNA droplets transcribed from gel-purified long and short Mut DNA sequences . 36
4.11 PCR products and RNA droplets resulting from PCR-amplified WT DNA with and without different sets of primers 37
4.12 Agarose gel electrophoresis and RNA droplets of the WT2 DNA after mutagenesis 38
4.13 Percentage of vacuolated droplets for WT2 and its mutated variants 40
4.14 Length Distribution of Sequenced Mut DNA and Predicted RNA Secondary Structure 41
4.15 De novo mutant designs induce vacuole formation 42
4.16 Estimation of mass concentration of Mut and WT RNA in the dilute phase using calibration curve 43
4.17 Spinodal regions in WT-Mut RNA phase diagram 44
4.18 Mechanism for vacuole formation 45
4.19 Measurement of transcription rate and threshold nutrient concentration 46
4.20 Estimated onset radius of vacuolation 47
4.21 Experimental validation of the theoretical predictions for vacuole formation . . . 48
4.22 Annealing of WT and Mut nanostars to test whether Mut acts as a surfactant . 50
4.23 Analysis of vacuole movement in relation to droplet movement 51
4.24 Burst-like movement of Mut RNA droplets 53
4.25 Distribution of angle α across two different replicates 54

5 Supplementary Videos 55
5.1 Supplementary Video 1 55
5.2 Supplementary Video 2 55
5.3 Supplementary Video 3 55
5.4 Supplementary Video 4 55
5.5 Supplementary Video 5 55
5.6 Supplementary Video 6 56

6 References 57

7 Uncropped gel scans 58

1 Supplementary Materials & Methods

1.1 Fluorescent labelling of DNA templates

To examine the localisation of DNA templates following RNA droplet formation, we fluorescently labelled the DNA using Fluorescein-12-dUTP (Thermo Scientific, Cat. No. R0101). Labelling was achieved by performing PCR in which 50% of the dTTP was replaced with Fluorescein-12-dUTP, following the manufacturer's recommendations. The amplified DNA was subsequently purified, using a Qiagen PCR purification kit (Cat. No. 28104), and analysed by agarose gel electrophoresis (AGE). In short, a 3% (W/V) agarose gel was prepared by dissolving agarose in 1X TAE buffer. After loading, the gel was run at $100\,\mathrm{V}$ for $1\,\mathrm{h}$ to $2\,\mathrm{h}$ in 1X TAE buffer. Incorporation of fluorescein was verified using the Trans-blue, $450\,\mathrm{nm}$ to $490\,\mathrm{nm}$ illumination settings on a ChemiDoc MP Imaging System (Bio-Rad Cat. No. 12003154).

1.2 Image analysis: quantification of droplet/vacuoles area and size

For batch processing and to analyse the size of the RNA droplets and quantify the vacuole area regions without bias, we implemented a custom Python pipeline that processes both *.tif* and *.czi* image files acquired via multi-channel fluorescence microscopy. In brief, for each image, the RNA fluorescence channel was extracted and normalised to its maximum intensity. A Gaussian blur ($\sigma = 5$ pixels) was applied to reduce noise, and two-class Otsu thresholding was used to segment the RNA-rich regions. To eliminate noise and artefacts, small objects below a dynamic size threshold (proportional to image resolution) were removed. The resulting binary mask was then labelled. As a control, the labelled masks were overlaid onto the original RNA image and saved as separate images. The overlay images were systematically inspected to verify the segmentation quality. For each detected droplet, morphological features were computed using the *skimage* package, including area, perimeter, major and minor axis lengths, mean RNA intensity, and filled area. We then determined the hollow area by subtracting the segmented area from the filled area, and used this to compute the fraction of vacuole area (A_{hollow}/A_{filled}). Finally, all extracted data and associated metadata (time point, condition, replicate, image ID) were compiled into a single CSV file for further plotting. The code for detection and plotting is available on our GitHub. Note that confocal z-stacks show that droplets remain largely spherical with minimal wetting on glass, supporting the use of area as a proxy for droplet radius (Supplementary Fig. 3).

1.3 Image analysis: line profile analysis

Line profiles were extracted in Fiji (ImageJ version 2.3.0/1.54f) using the Line tool with a line width of 50. Prior to analysis, images were smoothed using a Gaussian blur with $\sigma = 3$ to reduce noise. Intensity values along each line were exported as CSV files and subsequently plotted in Python, without further processing.

1.4 FRAP measurements

WT RNA droplets were prepared as described in the main text Materials and Methods, except that 20% of the rUTP was replaced with Fluorescein-12-UTP (Jena Bioscience, Cat. No. NU-821-FAMX-L). FRAP experiments were performed on a confocal microscope (LSM 900, Carl Zeiss AG) by bleaching the centre of individual RNA droplets using the 488 nm laser at maximum power. Bleaching was applied to the selected region of interest (ROI) for 50 iterations, after which image acquisition was resumed. Post-bleach images were recorded every 0.7 s. In addition to the bleached droplet, a background region and an unbleached control droplet were imaged to correct for background fluorescence and free fluorescent rUTP in solution.

1.5 Tracking movement of vacuolated RNA droplets

Confocal images of Mut and WT RNA droplets were acquired from the onset of transcription for at least 63 hours with an interval of 20 minutes. Time points prior to minute 580 (9h40) were excluded to observe the movement of exclusively stabilised droplets. Prior to this time point, the droplets undergo nucleation, growth and fusion. The time point was chosen based on the time at which the non-vacuolated RNA droplets reduce in fusion events. Note that minute 580 was

then zeroed in the plots to facilitate graph visualisation.
The tiff images of the time-lapses were then processed using ImageJ2 (v. 2.14.0/1.54f).[1] In brief, the images were binarised using *Convert to Mask function with Huang method*. The images were then subjected to two filling functions: *Fill Holes* and *Fill Border Holes* (from *Morphology* Plugins). The droplets in the processed images were then tracked using the *TrackMate* plugins.[2] The Detector method was set to Thresholding detector using default settings and all spots with radius above 4.97 microns were selected. The Tracker method was set to Nearest-neighbor tracker with Maximal linking distance of 5 micron. The tracks were then cleaned up manually to link fragmented trajectories and remove unsuccessfully filled vacuolated droplets. Upon fusion event of multiple droplet, the trajectory of the droplet with the largest area, was kept while the trajectories of the smaller ones were terminated. The exported XML files from the tracking was then used for further analysis and plotting using a custom-made code (see Code availability). The tracking experiments were done in two independent replicates with 7 fields of view. The data was pooled for analysis and plotting.

1.6 Tracking of vacuoles and movement direction analysis

Binarised images (see Section 1.5) were used for vacuole detection. The images were first Gaussian blurred with a sigma of 1, binarised using Otsu's method, and then inverted prior to detection. Detected vacuoles were filtered and associated with the corresponding droplets in the trajectories, as derived from XML files (see Section 1.5), using custom code (see Code availability). For droplets containing multiple vacuoles, the vacuole centre position was determined by either averaging the centres (mean position) or by weighting the centres based on each vacuole's area (weighted mean position).

Regardless of the method used to calculate the mean position, the mean vacuole centre was then used to assess the directionality of the vacuole's movement relative to that of the droplet's geometric centre, as illustrated in the schematic below. The vector $\vec{x}$ represents the direction of movement of the droplet's geometric centre. The displacement of the vacuole centre relative to the droplet is denoted by $\vec{v}$, which is calculated as the difference between $\vec{v}_1$ and $\vec{v}_0$. Here, $\vec{v}_0$ and $\vec{v}_1$ represent the vectors from the geometric centre of the droplet to the vacuole centre at two consecutive time points, with $\vec{v}_0$ corresponding to the earlier time and $\vec{v}_1$ to the later time. Angle α represents the angle between $\vec{v}$ and $\vec{x}$, where $\alpha > 90°$ indicates opposite directions of movement. The angle α is calculated for each pair of subsequent time frames where vacuoles are detected. Only the angles of movement greater than $1\,\mu\mathrm{m}$ were used for the histograms shown here.

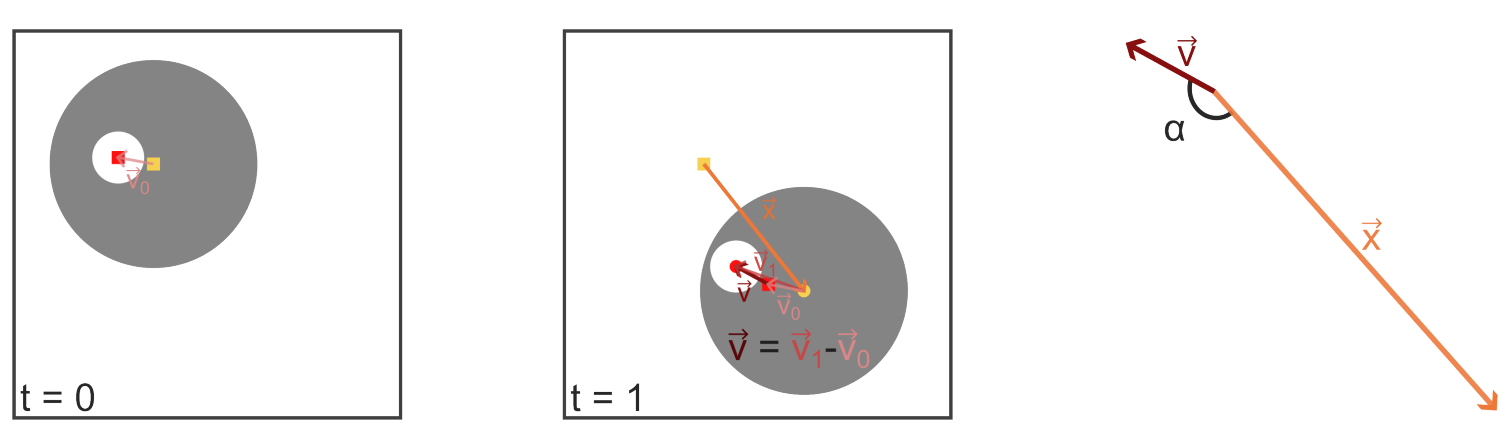

1.7 Droplet fusion and relaxation analysis

To analyse droplet fusion events, we used the time-lapse data from Section 1.5. Each frame was preprocessed and segmented using a custom Python pipeline. Briefly, a median filter (disk footprint, radius 2 pixels) was applied to reduce noise while preserving droplet boundaries. The filtered images were binarised using Otsu's thresholding method, and the resulting binary masks were refined by morphological opening followed by closing (disk footprint, radius 1 pixel) to remove spurious regions and fill minor gaps. The images were segmented using connected-component labelling (8-connected in 2D). Segmentation quality was verified visually using the Napari viewer.

To quantify the relaxation dynamics of individual condensates following a fusion event, ellipses were fitted to manually selected fusing droplets. The onset of each fusion event was identified manually, and an ellipse was fitted to the boundary of selected droplet using *skimage.measure.regionprops* at each of the 6 consecutive timeframes (120 minutes) following the onset. The aspect ratio q was computed at each time point as a major axis length divided by minor axis length. Its temporal evolution was fitted with an exponential decay:

$$q(t) = 1 + A\, e^{-t/\tau}, \quad (1)$$

where τ is the relaxation time.

The characteristic length L was defined as the mean of the half lengths of the major and minor axes at the onset of the fusion event. The relaxation time τ scales linearly with the characteristic length as $\tau \approx v_c^{-1} \cdot L$, where the slope corresponds to the inverse capillary velocity $v_c^{-1} = \eta/\gamma$, with η the viscosity and γ the surface tension of the condensate. A linear fit was applied to the pooled data of both replicates to extract v_c^{-1} for the conditions of WT and Mut separately. Note that for Mut droplets, only smaller non-vacuolated droplets were analysed.

1.8 DNA sequencing and analysis

For sequencing, 100fmol of the Mut DNA was barcoded and library prepped using the Native Barcoding kit (SQK-NBD114.24, Oxford Nanopore Technologies) and subsequently sequenced (for 24h) on a Flongle (R10.4.1) flowcell using the Mk1D sequencer. Basecalling was performed live during the run through Nanopore's MinKnow Software utilising the latest super accuracy model. Full-length reads were processed and error-corrected using the following bioinformatics pipeline:

1. Pychopper for read trimming and orientation (`https://github.com/epi2me-labs/pychopper?tab=readme-ov-file`)
2. isONclust[3] for clustering similar reads
3. isONcorrect[4] for error correction based on identified clusters

All tools were run with default parameters. The corrected sequences were then aligned to the original WT sequence using minimap2[5] with default parameters. Analysis of the alignment results revealed that 94.66% of reads aligned perfectly to the WT reference sequence. Additionally, approximately 3.1% of reads were identified as chimeric, with different segments aligning to distinct regions of the WT reference. These chimeric reads were detected using a custom script

that identified reads containing supplementary alignments in the BAM file. Further, we identified the four most prevalent types of chimeric alignments. The length of these chimeric reads corresponded to the non-specific bands previously observed on AGE of WT PCR products, confirming their presence in the sequenced DNA library. We constructed an expanded reference database that included the WT sequence plus the representative sequences for each of the four chimeric types (see Supplementary Table 3). All reads were then realigned to this expanded reference using minimap2, allowing us to accurately quantify the percentage of reads corresponding to each distinct DNA variant, as shown in Supplementary Fig. 14.

1.9 Annealing of RNA condensates in water-in-oil droplets

WT and Mut DNA templates were transcribed overnight using the HiScribe T7 transcription kit (NEB, Cat. No. E2040S) according to the manufacturer's instructions. The resulting RNA was purified by column purification using the RNA Clean & Concentrator kit (Zymo Research, Cat. No. R1019). A mixture of 63% WT and 37% Mut RNA by RNA mass was prepared in dilution buffer ($100\,\mathrm{mM}$ HEPES pH 7.5, $1.5\,\mathrm{mM}$ spermidine, $20\,\mathrm{mM}$ $Mg(OAc)_2$, $1.5\,\mathrm{mM}$ DTT (freshly prepared), $0.5\,\mathrm{mM}$ EDTA, $50\,\mathrm{\mu M}$ Malachite Green, and $50\,\mathrm{\mu M}$ DFHBI-1T) at a final RNA concentration of $1000\,\mathrm{ng\,\mu L^{-1}}$.

Water-in-oil droplets were formed by adding $40\,\mathrm{\mu L}$ of fluorinated oil (2% Pico-Surf in Novec™ 7500) to $20\,\mathrm{\mu L}$ of the RNA mixture. The emulsion was generated by repeated pipetting with a $200\,\mathrm{\mu L}$ tip until the suspension appeared uniformly opaque, and then centrifuged at 400 $\times g$ for 4 minutes.

The water-in-oil droplets were first equilibrated at $37\,°\mathrm{C}$ for 4 hours and subsequently transferred to PVA-coated glass slides for imaging. The microscope stage was heated to $50\,°\mathrm{C}$ and the droplets were equilibrated for 1 hour. The stage heating was then turned off, and the sample was allowed to cool passively to room temperature over the course of 1 hour until condensates size no longer increased. Images for analysis were acquired after this final equilibration step.

1.10 Experimental phase diagram

For calibration curves, shown in Supplementary Fig. 16, kissing loop motifs in WT with MGA and Mut RNA with BrA sequences were replaced with tetraloops to prevent phase separation (sequences listed in Supplementary Table 1).

All DNA templates were transcribed using the HiScribe™ T7 High Yield RNA Synthesis Kit (New England Biolabs) at $37\,°\mathrm{C}$ for $48\,\mathrm{h}$ in a $100\,\mathrm{\mu L}$ reaction volume. RNA was purified using the Zymo Research RNA Clean & Concentrator kit according to the manufacturer's protocol.

RNA samples were serially diluted two-fold from $200\,\mathrm{ng\,\mu L^{-1}}$ down to $3.125\,\mathrm{ng\,\mu L^{-1}}$ in dilution buffer ($100\,\mathrm{mM}$ HEPES (pH 7.5), $1.5\,\mathrm{mM}$ spermidine, $20\,\mathrm{mM}$ $Mg(OAc)_2$, $1.5\,\mathrm{mM}$ DTT (freshly prepared), $0.5\,\mathrm{mM}$ EDTA, $50\,\mathrm{\mu M}$ Malachite Green and $50\,\mathrm{\mu M}$ DFHBI-1T). Fluorescence was measured in a sealed 384-well clear-bottom plate (Greiner Bio-One 781906) with $30\,\mathrm{\mu L}$ per well using a microplate reader (Tecan Spark). Excitation and emission wavelengths were set as follows: Malachite Green aptamer, excitation $620\,\mathrm{nm}$ and emission $660\,\mathrm{nm}$; Broccoli aptamer, excitation $485\,\mathrm{nm}$ and emission $507\,\mathrm{nm}$. Each dilution point was measured in two replicates, along with one negative control (no RNA). Background fluorescence from the negative control was subtracted, and linear regression was fitted to the fluorescence–concentration data (see

Supplementary Fig. 16). The resulting calibration equations were later used to determine RNA concentrations in dilute phase samples.

Phase separation reactions were performed in $10\,\mu\mathrm{L}$ volumes under the same buffer conditions as above. Four representative conditions were used, initial concentrations of WT and Mut are listed in Supplementary Table 4. Reactions were incubated at $37\,^{\circ}\mathrm{C}$ for 1 h, then centrifuged at 400×g for 4 min to sediment the condensed phase. Each condition was measured in 3 technical replicates, from a total of 2 independent replicates. To measure RNA concentration in the dilute phase (C^{out}), $6\,\mu\mathrm{L}$ of the supernatant was withdrawn and diluted to $30\,\mu\mathrm{L}$ with dilution buffer for plate reader measurements. Concentrations were calculated from the calibration curves (Supplementary Fig. 16). The total volume of the supernatant was also measured to determine the volume of the dilute phase (V^{out}). The condensed phase volume was estimated as

$$V^{\text{in}} = V^{\text{total}} - V^{\text{out}}.$$

The RNA mass concentration in the condensed phase was calculated as

$$C^{\text{in}} = \frac{C^{\text{initial}} \cdot V^{\text{total}} - C^{\text{out}} \cdot V^{\text{out}}}{V^{\text{in}}}.$$

1.11 Transcription rate measurement on plate reader

To monitor the accumulation of transcribed RNA under our experimental conditions, WT2 DNA was transcribed as described in the Methods section of the main text, with the addition of RNase inhibitor at a final concentration of $1\,\mathrm{U}/\mu\mathrm{L}$ (NEB, Cat. No. M0314S). The WT2 nanostar contains an embedded Broccoli aptamer, enabling the accumulation of Broccoli-containing RNA to be monitored through fluorescence following binding of DFHBI-1T.[6] DFHBI-1T was added at a final concentration of $100\,\mu\mathrm{M}$. Reactions were carried out in a PVA-passivated, non-binding 384-well microplate with a transparent bottom (Greiner, Cat. No. 781906). The wells were passivated with PVA as described in the main text. Each $40\,\mu\mathrm{L}$ reaction was covered with $10\,\mu\mathrm{L}$ of mineral oil to prevent evaporation.

The microplate reader (Spark multimode microplate reader, Tecan) was preheated to $37\,^{\circ}\mathrm{C}$. Fluorescence was measured from the bottom of the plate using excitation and emission wavelengths of $485\,\mathrm{nm}$ (bandwidth of $7.5\,\mathrm{nm}$) and $507\,\mathrm{nm}$(bandwidth of $7.5\,\mathrm{nm}$), respectively, at intervals of $3\,\mathrm{min}$ for a total of $45\,\mathrm{h}$. The gain was manually set to 55. Monitoring was initiated immediately after mixing the reaction components, with the first measurement defined as (t=0).

1.12 Thermodynamic model for the phase diagram

We propose a thermodynamic description for RNA nanostar phase separation based on their mass concentrations c_i [units: ng/μL]. We consider the ternary mixture composed of solvent and two different labelled RNA nanostars discussed in Sect. 1.10: (i) wild-type RNA ("WT"), (ii) mutant RNA ("Mut"). Thus, the label $i =$WT,Mut. Experimentally, the wild-type and mutant RNA-nanostars are the most abundant macromolecules in the solution. Other components in the transcription experiments are, for example, the polymerase and the DNA. Due to their low abundance, their effect on the phase diagram can be neglected.

Moreover, two phases were observed experimentally: a RNA-nanostar-rich phase ("in") and a RNA-nanostar-poor phase ("out"). This phase separation can be captured by an effective free energy density $f(\{\phi_i\})$. The free energy depends on the RNA nanostars volume fractions

$$\phi_i = c_i/\rho_i \, , \tag{2}$$

where c_i is the mass concentrations. Moreover, $\rho_i = m_i/\nu_i$ is the molecular mass density of component i with m_i and ν_i as the RNA nanostar molecular mass and molecular volume, respectively. We fix the molecular mass density of the two nanostars to a typical value known for oligonucleotides, $\rho_{\mathsf{WT}} = \rho_{\mathsf{Mut}} \simeq 1.7\,kg/L$. The values of ν_i are obtained by fitting to experimental data (details on fitting the experimental data, see below), which determines together with the chosen value of ρ_i the respective molecular masses m_i.

For the ternary (incompressible) nanostar mixture, the effective free energy density $f(\phi_{\mathsf{WT}}, \phi_{\mathsf{Mut}})$ depends on the wild-type RNA volume fraction ϕ_{WT} and the mutant RNA volume fraction ϕ_{Mut}:

$$\begin{aligned} f(\phi_{\mathsf{WT}}, \phi_{\mathsf{Mut}}) = \frac{k_{\mathsf{B}}T}{\nu_{\mathsf{sol}}} \Bigg[& \frac{\phi_{\mathsf{WT}}}{\xi_{\mathsf{WT}}} \log(\phi_{\mathsf{WT}}) + \frac{\phi_{\mathsf{Mut}}}{\xi_{\mathsf{Mut}}} \log(\phi_{\mathsf{Mut}}) \\ & + (1 - \phi_{\mathsf{WT}} - \phi_{\mathsf{Mut}}) \log(1 - \phi_{\mathsf{WT}} - \phi_{\mathsf{Mut}}) \\ & + \chi_{\mathsf{WT,sol}}\, \phi_{\mathsf{WT}} (1 - \phi_{\mathsf{WT}} - \phi_{\mathsf{Mut}}) \\ & + \chi_{\mathsf{Mut,sol}}\, \phi_{\mathsf{Mut}} (1 - \phi_{\mathsf{WT}} - \phi_{\mathsf{Mut}}) \\ & + \chi_{\mathsf{WT,Mut}}\, \phi_{\mathsf{WT}} \phi_{\mathsf{Mut}} \Bigg] \, , \end{aligned} \tag{3}$$

where $k_{\mathsf{B}}T$ is thermal energy scale and the volume fraction of the solvent is given as $(1 - \phi_{\mathsf{WT}} - \phi_{\mathsf{Mut}})$. Moreover, $\xi_i = \nu_i/\nu_{\mathsf{sol}}$ is the relative fraction of molecular volumes compared to the solvent with i =WT,Mut. The interactions among the nanostars and with the solvent are described by interaction parameters

$$\chi_{ij} = 2e_{ij} - e_{ii} - e_{jj} \, , \tag{4}$$

where i and j are the respective interaction components. Here, e_{ij} is the mean interaction strength between i and j (units of $k_{\mathsf{B}}T$), while e_{ii} represents the mean self-interaction of a nanostar component i. The sign of e_{ij} determines whether the interaction is attractive ($e_{ij} < 0$) or repulsive ($e_{ij} > 0$).

The free energy (Eq. (3)) can be used to fit (least square fitting) the experimentally observed phase diagram with the physical parameters, particularly two interaction parameters $\chi_{\mathsf{WT,sol}}, \chi_{\mathsf{Mut,sol}}$ and $\chi_{\mathsf{WT,Mut}}$ and the two molecular volumes ν_{WT} and ν_{Mut} using least square fitting. Mathematically, the phase diagram is obtained from the free energy density (Eq. (3)) through solving the algebraic conditions for phase equilibrium that correspond to equal chemical potentials $\mu_i = \nu_i \partial f/\partial \phi_i$ and equal osmotic pressure $\Pi = -f + \sum_{i=\mathsf{Mut,WT}} \mu_i/\nu_i$ between the two coexisting phases labelled as "in" and "out", respectively:

$$\mu_i^{\mathsf{in}} = \mu_i^{\mathsf{out}} \, , \quad i = \text{Mut,WT} \, , \tag{5a}$$

$$\Pi^{\mathsf{in}} = \Pi^{\mathsf{out}} \, . \tag{5b}$$

Numerical solutions were obtained by shooting and initializing the system starting from dilute mutant concentrations using analytic expressions for dilute partitioning in multi-component mixtures.[7]

The conditions (5) give the saturation mass concentrations of both RNA nanostars above which phase separation ("out") can occur, as well as the mass concentrations in the nanostar droplet ("in") at phase equilibrium. We note that the mass concentrations can be computed from the volume fractions using Eq. (2). Fig. 4b in the main text shows such a phase diagram fit to the experimentally measured mass concentrations in each phase. Without mutant RNA, the wild-type RNA-rich phase has roughly 400-fold higher mass concentration than the RNA-poor phase. Upon adding mutant RNA, the phase separation propensity is almost unaffected, which is indicated by an approximately vertical slope of the RNA-poor branch of the phase diagram.

From the fits, we extract the nanostar molecular volumes and the respective interaction parameters (see Table 5). Specifically, we find that the molecular volumes of both RNA nanostars are very similar ($\nu_{\text{WT}} \simeq 10^2\,\mathrm{L\,mol^{-1}}$), and ($\nu_{\text{Mut}} \simeq 1.5\,\nu_{\text{WT}}$), which is consistent with the mutant having only one arm that is slightly longer than the wild-type (see main text, Fig. 4a). Moreover, the fitted molecular volumes are consistent with the following simple estimate: for a WT-RNA chain composed of roughly 265 nucleotides, $\nu_{\text{WT}} \simeq 265\,\nu_{\text{rNTP}}$, where $\nu_{\text{rNTP}} \simeq 0.3 - 0.5\,\mathrm{L\,mol^{-1}}$ are common value of the molecular volume of a single nucleotide.[8,9] Moreover, we find that nanostar phase separation is driven by adhesive interactions among the nanostars of the same type, while interactions with the solvent are weak compared to the adhesive interaction between WT-nanostars. Finally, the Mut-Mut interactions turn out to be less adhesive ($e_{\text{Mut,Mut}} > e_{\text{WT,WT}}$) than the WT-WT interactions, which is consistent with the longer arm exhibiting a weaker kissing loop interaction. We note an apparent discrepancy in the RNA-poor branch between the model fit and the experimental data: the model has a negative slope, whereas the experimental data indicate a positive slope. However, the slope for both the model and experiment is approximately vertical, and thus represents a (in the log-plot) visible but minor quantitative discrepancy of in matching compositions between model and experiments. Most importantly, the model fit and the experiment agree very well in terms of the tie lines, which will turn out to dictate one of the key thermodynamic properties for the vacuole instability. Thus, among fits with comparable error, we preferred the ones that had a better agreement with the experimental tie line.

1.13 Theoretical model for the onset of vacuole formation

Here, we discuss a kinetic model for the growth of the RNA nanostar droplets and the kinetics of the nanostar concentration fields to predict the onset of vacuole formation. We will see that kinetic parameters of the system, such as diffusivities and transcription rate, are crucial determinants, in addition to the nanostar interactions that were obtained from fitting the experimental phase diagram (see discussions in Sections 1.10 and 1.12). We will consistently use volume fraction fields $\phi_i(\boldsymbol{x}, t)$ for all components i in the following, where $\boldsymbol{x}$ is position and t time. Please note that the corresponding mass concentrations can be converted from the volume fractions via Eq. (2).

1.13.1 Transcription reaction, model parameters and constraints

Transcription of RNA from DNA through the T7 polymerase can be described as chemical reactions with the following reaction scheme:

$$\text{rNTP} + \text{WT-DNA} \longrightarrow \text{WT-RNA}\,, \quad \text{rNTP} + \text{Mut-DNA} \longrightarrow \text{Mut-RNA}\,. \tag{6}$$

The two different DNAs can transcribe two different RNAs, namely: (i) wild-type nanostars (WT-RNA) and (ii) mutant nanostars (Mut-RNA). The fraction of mass concentrations between mutant Mut-DNA and WT-DNA

$$\lambda := \frac{\phi_{\text{Mut-DNA}}}{\phi_{\text{WT-DNA}}} \tag{7}$$

is an important parameter in the model and in the experiments. When initializing the system without RNA-nanostars at $t = 0$, the production of WT-RNA and Mut-RNA from the "nutrient" rNTP (ribonucleoside triphosphate) being at excess fixes the fraction of transcribed RNA nanostars to the mutant DNA fraction when the system is homogeneous:

$$\frac{\phi_{\text{Mut-RNA}}}{\phi_{\text{WT-RNA}}} = \lambda \quad \text{for} \quad 0 < t < \tilde{t}\,. \tag{8}$$

In other words, as long as nutrients rNTP do not get depleted before, the RNA mass concentrations increase in time, keeping their fraction constant until at $t = \tilde{t}$ phase separation occurs. In this case, the fraction of the mutant DNA λ determines at which saturation concentrations the system crosses the dilute phase branch of the phase diagram (main text, Fig. 4b). In other words, the freshly nucleated RNA-nanostar droplets have a composition determined by the value of λ, which is illustrated in Supplementary Fig. 18b showing the selected tie-line for a particular λ. Note that the later paragraphs explain how droplets behave after they have formed.

To keep the model minimal in terms of the number of components, we introduce an effective solvent component that consists of the rNTP nutrients, and the solvent components (e.g., water, buffer, etc.). The latter do not participate in the chemical reaction. This minimal setting can be modeled as a ternary mixture with components WT-RNA, Mut-RNA and the effective solvent labelled as "sol" with the corresponding volume fraction fields ϕ_{WT}, ϕ_{Mut}, and ϕ_{sol}. Transcription and diffusion give rise to spatial heterogeneities, and interactions in between these three components can lead to phase separation into a RNA-nanostar-rich phase ("in") and a RNA nanostar-poor phase ("out"). From the phase diagram fit, we identified the interaction parameters between the different RNA nanostars ($e_{\text{WT,WT}}$, $e_{\text{Mut,Mut}}$, $e_{\text{WT,Mut}}$) as attractive (see previous section). In other words, both RNA nanostars (WT and Mut) colocalise and jointly form a condensed droplet of radius R.

We characterize the phase-separation kinetics in the nanostar mixture using a narrow interface model. In this model, the interface is considered to be narrow compared to the size of the droplets. Moreover, the two phases (droplet "in", and RNA nanostar poor phase "out") are connected by a moving boundary, the interface. The typical condition for liquid droplets that holds at the interface is phase equilibrium, through which the thermodynamic properties discussed in the last section enter the theoretical model. In the following, we discuss the model equations starting from the kinetics in the RNA-nanostar-poor phase (Sect. 1.13.2), over the kinetics of and at the interface (Sect. 1.13.3), to the RNA-nanostar-rich phase (Sect. 1.13.4).

1.13.2 Kinetics in RNA-nanostar poor phase

Since diffusion is fast in the RNA-nanostar-poor phase, mass concentrations and volume fraction fields are considered to be approximately homogeneous outside ("out") the droplets. This approximation is justified since reaction-diffusion length scales for all components outside the droplets are in the order of 10 μm, which is in the order of the inter-droplet distances. For example, the

reaction-diffusion length scale outside the droplets, $\sqrt{D^{\text{out}}/k} \simeq 20\mu m$ for a diffusion coefficient of $D = 100\mu m^2/s$ (see Ref.[10] for DNA-nanostars) and the rate of transcription for an RNA of length $L_{\mathrm{WT-RNA}} = 265\,\mathrm{nt}$ was estimated in our studies as $k \simeq 0.187s^{-1}$ (see sect. 2.1 for a detailed calculation).

In the model, we assume that transcription occurs dominantly in the RNA-poor phase while transcription inside the droplet can be neglected. This assumption is justified since the poor-phase volume exceeds the total droplet volume by 2 orders in magnitude ($V^{\text{out}} \gg V^{\text{in}}$). During the transcription reaction described in Eq. (6), the amount of available DNA sets a limit on the overall reaction rate. Each DNA-polymerase complex can only bind to a fraction of nutrient molecule from the total available nutrient at a given time t when the nutrients are in excess. In this regime, the reactions are only dictated by the i-DNA, and the concentration of nutrients has no effect on reaction rate; in other words, the T7 polymerase performs in the nutrient excess regime. In contrast, when nutrients get depleted, only a small fraction of DNA-polymerase complex is active, therefore the rate of transcription is no longer just DNA-limiting but also scale linearly with the amount of nutrients, meaning that the T7 polymerase performs in the nutrient depleted regime. To capture both of these limits, we use a Hill-form for the polymerase kinetics and write the rate for depletion of rNTP as follows:

$$\frac{\mathrm{d}\phi^{\text{out}}_{\text{rNTP}}(t)}{\mathrm{d}t} = -k \frac{\frac{\nu_{\text{WT-RNA}}}{\nu_{\text{WT-DNA}}}\phi^{\text{out}}_{\text{WT-DNA}} + \frac{\nu_{\text{Mut-RNA}}}{\nu_{\text{Mut-DNA}}}\phi^{\text{out}}_{\text{Mut-DNA}}}{1 + \dfrac{\alpha}{\phi^{\text{out}}_{\text{rNTP}}(t)}} - \frac{\phi^{\text{out}}_{\text{rNTP}}}{V^{\text{out}}}\frac{\mathrm{d}V^{\text{out}}}{\mathrm{d}t}\,. \tag{9}$$

Here, α denotes the threshold rNTP volume fraction when the T7 polymerase enter the rNTP limited regime. For $\phi^{\text{out}}_{\text{rNTP}}(t) \gg \alpha$ the rates saturates to the maximum in WT-RNA volume fraction and becomes independent of the nutrients. For $\phi^{\text{out}}_{\text{rNTP}}(t) \ll \alpha$, the rate scales linearly with the available nutrients. The initial nutrient volume fraction $\phi^{\text{out}}_{\text{rNTP}}(t=0)$ is obtained from the experimental rNTP concentration ($n_{\text{rNTP}} = 10\,\mathrm{mM}$; see Methods section in the main text) using the relation $\phi_{\text{rNTP}} = \nu_{\text{rNTP}}\, n_{\text{rNTP}}$. In Eq. (9), k [unit: 1/time] is the effective transcription-rate for a DNA template of length $L_{\text{WT-DNA}}$. Both α and k were estimated from independent transcription experiments by fitting the measured RNA concentration dynamics to the kinetic model (see Sect. 2.1 for details). Since the model is written in terms of volume fractions, the fitted threshold nutrient concentration K_α is converted into the corresponding rNTP volume fraction using $\alpha = \nu_{\text{rNTP}} K_\alpha$. In summary, Eq. (9) captures that transcription slows down once nutrients get depleted in the system. We note that the volume fraction of DNA is considered to be constant.

Now we can write the production of both RNA-nanostars through transcription. The average volume fraction of RNA-nanostars ϕ_i ($i \in$ WT, Mut) in the RNA-nanostar-poor phase follows the following kinetic equation:

$$\frac{\mathrm{d}\phi^{\text{out}}_{\text{i-RNA}}(t)}{\mathrm{d}t} = \Gamma\, k \frac{\frac{\nu_{\text{i-RNA}}}{\nu_{\text{i-DNA}}}\phi^{\text{out}}_{\text{i-DNA}}}{1 + \dfrac{\alpha}{\phi^{\text{out}}_{\text{rNTP}}(t)}} - j_i^{\text{out}} - \frac{\phi^{\text{out}}_{\text{i-RNA}}}{V^{\text{out}}}\frac{\mathrm{d}V^{\text{out}}}{\mathrm{d}t}\,. \tag{10}$$

In general, the transcription reactions can have other by-products; therefore, the production of WT and Mut-RNA should decrease by a factor Γ, which we call as yield-factor. If $\Gamma = 1$, the production of i-RNA is 100% efficient, i.e., the rNTP mass is converted to RNA by 100%. In

this work, we consider equal yield $\Gamma = 0.5$ for both WT and Mut RNA-nanostars. In Eq. (10), the term j_i^{out} is the diffusive exchange flux density between the phases, which maintains phase coexistence at the phase boundary (i.e, Eqs. (11)). Details are given in the following section.

1.13.3 Droplet growth: Kinetics of droplet interface

We continue with the equations governing the dynamics of the moving interface corresponding to droplet growth and the transport through the interface. To this end, we consider a single spherical droplet of radius $R(t)$ and the radial position r. The radius $R(t)$ increases in time due to transcription (droplet growth). The droplet is nucleated with radius $R(t = \tilde{t}) = R_0$ at time $\tilde{t}$ in a system (Supplementary Fig. 18a). At that time, the RNA-nanostar composition of the nanostar-poor phase is set by fraction of the mutant DNA λ (Eq. (8)), while the droplet composition is determined by the phase coexistence conditions (see Supplementary Fig. 18b). In the model, this initial radius is chosen to be much smaller than the final radius reached once all the rNTPs are depleted. When $R_0 \ll \sqrt{D^{\text{in}}/k}$, the results for the formation of vacuoles are independent of the choice of the initial radius R_0.

We assume that the RNA-nanostar-poor phase is at phase equilibrium with the droplet interface. To write the condition of local phase equilibrium in a spatially heterogeneous system, we introduce the radial position R_+ and R_- as the interface position when approaching the interface from the outside (out) and inside (in) phase, respectively. The condition of phase equilibrium at the interface then reads:

$$\bar{\mu}_i^{\text{out}}(R_+, t) = \bar{\mu}_i^{\text{in}}(R_-, t)\,, \tag{11a}$$

$$\Pi^{\text{out}}(R_+, t) = \Pi^{\text{in}}(R_-, t)\,. \tag{11b}$$

We can calculate the diffusive exchange fluxes densities at the interface j_i^{out}, as introduced in Eq. (10), from the condition that the change in chemical potentials and osmotic pressure obeys:

$$\partial_t \bar{\mu}_i^{\text{out}}(R_+, t) = \partial_t \bar{\mu}_i^{\text{in}}(R_-, t)\,, \tag{12a}$$

$$\partial_t \Pi^{\text{out}}(R_+, t) = \partial_t \Pi^{\text{in}}(R_-, t)\,. \tag{12b}$$

See Ref.[11] for details regarding the derivation of diffusive exchange flux.

Now we discuss the kinetic equations governing the interface movement. The influx volume of WT-RNA, $J_{\text{WT}}^{\text{in}}$, Mut-RNA, $J_{\text{Mut}}^{\text{in}}$, and solvent $J_{\text{sol}}^{\text{in}}$ from nanostar-poor phase causes a change in droplet volume $V^{\text{in}}(t)$ corresponding to a moving interface position $R(t)$. Since the total volume of the system $V = V^{\text{in}} + V^{\text{out}}$ remains constant, the volume changes in the RNA-nanostar-rich and -poor phase are coupled:

$$\frac{\mathrm{d}V^{\text{in}}(t)}{\mathrm{d}t} = -\frac{\mathrm{d}V^{\text{out}}(t)}{\mathrm{d}t}\,, \tag{13}$$

where the volume change of the RNA-nanostar-poor phase can be expressed by the fluxes of WT, Mut and solvent through the interface:

$$\frac{\mathrm{d}V^{\text{out}}}{\mathrm{d}t} = -(j_{\text{WT}}^{\text{out}} + j_{\text{Mut}}^{\text{out}} + j_{\text{sol}}^{\text{out}})V^{\text{out}}\,, \tag{14}$$

where

$$J_i^{\text{out}} = j_i^{\text{out}} V^{\text{out}} \tag{15}$$

is the net volume of particles leaving the RNA poor phase. We can write the movement of the interface position $R(t)$ as follows:

$$\frac{\mathrm{d}R(t)}{\mathrm{d}t} = \frac{1}{4\pi R(t)^2}\frac{\mathrm{d}V^{\text{in}}(t)}{\mathrm{d}t}\,, \tag{16}$$

where material conservation dictates that the net exchange of volumes between phases obeys $J_i^{\text{in}} = -J_i^{\text{out}}$.

Conditions given in Eq. (12), together with the assumption that diffusion in the RNA-nanostar-poor phase is fast leading to homogeneous profiles, gives a simple picture. When a RNA-nanostar is transcribed outside the droplets, it diffuses quickly towards the interface such that the outside phase and the interface remain at phase equilibrium. Most importantly, this volume flux makes the droplet grow and changes its composition inside, in general, leading to gradients from the droplet interface to the centre. In the next section, we derive the kinetic equations governing this concentration gradient that will turn out to be crucial for the formation of the vacuole.

1.13.4 Kinetics in RNA-nanostar rich droplet phase

In this section, we discuss the kinetic equation and conditions inside the RNA-nanostar-rich phase. In our model, we neglect the transcription inside the droplets relative to the RNA-nanostar-poor phase. However, the transcribed RNA-nanostars in the poor phase enter the droplet with an influx of WT-RNA, $j_{\text{WT}}^{\text{out}}$, and of Mut-RNA, $j_{\text{Mut}}^{\text{out}}$. Due to the strong attractive interactions among the nanostars, the diffusion coefficients inside the droplet are much lower than in the RNA-nanostar-poor phase:

$$D_i^{\text{in}}/D_i^{\text{out}} \ll 1 \tag{17a}$$

with i =WT,Mut and D_i^{in} and D_i^{out} denoting the diffusion coefficients in the respective phases. Such very different diffusivities between the phases are a general kinetic prerequisite for the formation of concentration gradients and thereby for the formation of vacuoles.

Since we neglected transcription inside the droplet, the dynamic equation is simply a linear diffusion equation within the narrow interface model:

$$\partial_t \phi_i^{\text{in}}(r,t) = D_i^{\text{in}} \nabla^2 \phi_i^{\text{in}}(r,t)\,. \tag{17b}$$

To determine the volume fraction profile in time $\phi^{\text{in}}(r,t)$ for $r < R(t)$, we solve Eq. (17b) numerically with the following boundary conditions: a no-flux condition at the droplet centre ($r = 0$) and a time-dependent Dirichlet condition at the interface ($r = R(t)$),

$$\boldsymbol{e}_r \cdot \nabla \phi_i^{\text{in}}\big|_{r=0} = 0\,, \tag{17c}$$

$$\phi_i^{\text{in}}(r = R_-, t) = \phi_i^{\text{in}}(t)\,, \tag{17d}$$

for i =WT,Mut and $\boldsymbol{e}_r$ is the normal vector in the radial direction. The latter condition is obtained from the phase equilibrium relations in Eq. (12). This means that the RNA-nanostar-rich phase composition, in general, can deviate from phase equilibrium due to slow diffusion. However, the composition of the moving interface is always maintained at phase equilibrium. This creates a gradient in the RNA-nanostar-rich phase, i.e, $\nabla \phi_i^{\text{in}}(r,t) \neq 0$. On longer times, these gradients then propagate toward the centre of the droplet. This kinetics can trigger a spinodal instability locally, forming a new interface; a vacuole is formed.

1.13.5 Physical mechanism of vacuole formation

In this section, we summarize the kinetics prior to vacuole formation and discuss the mechanism in more detail than in the main text. Overall, we note that the instability is of kinetic origin, i.e., the non-equilibrium transcription process gives rise to a spinodal instability of the droplets forming vacuoles. However, there are thermodynamic prerequisites for instability to occur that are essentially governed by the phase diagram with the binodal and the spinodal, as well as the tie lines as key properties. When nutrients get depleted on very long times, the system will relax to thermodynamic equilibrium corresponding to a phase-separated state with phases devoid of vacuoles.

Gradient formation: Right after nucleation, the initially small droplets grow by the influx of transcribed nanostars from the outside phase. However, due to their small size, diffusion inside is fast compared to the influx, leading to flat concentration profiles inside the droplet right after nucleation (see Supplementary Fig. 18a,b, blue profile which is flat inside with a composition set by the initial tie-line indicated by the left blue dot in b in the phase-diagram). When droplets grew bigger fed by the continuous influx of transcribed nanostars, the fast molecular exchange through the droplet interface can change the composition inside close to the interface. The thermodynamic requirement for such a change is that the angle between the tie line of the interface deviates from the line corresponding to the constant Mutant-WT-DNA fraction λ (highlighted by a grey region in Supplementary Fig. 18b). This change can build up a gradient from the droplet interface to the centre, where the composition at the droplet centre can lag behind, particularly when droplets grew big enough (see Supplementary Fig. 18a for the profile plotted in violet where the centre composition at $r = 0$ remained almost unchanged during growth composition). The gradient is more pronounced when the influx of transcribed material is fast compared to diffusion inside the droplet. Specifically, the reaction-diffusion length scale $\sqrt{D_i^{\text{in}}/k}$ has to be lower than the droplet radius R, which also highlights that bigger droplets in general give rise to more pronounced concentration gradients. We also note that when the tie-line is parallel to the λ-line, droplets just grow without altering the composition inside close to the interface. For that case, no gradients can form, independent of the diffusivity inside the droplet. In other words, the thermodynamic prerequisite of the vacuole instability, corresponding to the λ-line deviating from the tie line, is not fulfilled.

Vacuole formation: A vacuole forms when the concentration gradient is strong enough such that the compositions inside the droplet (e.g., at the centre) approach or even cross the spinodal line in the phase diagram (marked by a star symbol in Supplementary Fig. 18c). The spinodal line describes a linear and local diffusive instability through which a new interface emerges, i.e., a vacuole is formed. We find that a vacuole cannot form when droplets are too small since diffusion homogenises concentration gradients inside the droplets (Supplementary Fig. 18a). Moreover, the instability is favoured when the diffusivity inside of the mutant is higher than the one of the WT ($D_{\text{Mut}}^{\text{in}} > D_{\text{WT}}^{\text{in}}$; see Table 7 for the used values) since this leads to more pronounced relative gradients of WT to Mut, favoring intersection with the spinodal line. Since we have not included fluctuations in our model, we construct an effective spinodal lying between the binodal and the actual spinodal (Supplementary Fig. 17) to avoid discarding cases that closely approach the actual spinodal but do not cross it. By this effective spinodal, we effectively model the existence of the many other components, such as the T7 polymerase and the DNA that even condenses in small puncta, with the possibility to support triggering the spinodal instability.

The model describes the system as a liquid mixture composed of RNA nanostars as the most abundant molecular components, where vacuoles emerge from concentration gradients inside the dense phase that arise from transcription of such nanostars. For simplicity, the DNA templates and RNA polymerase are not included as explicit droplet-forming components (see Sect. 1.12 for the phase diagram). The reason is that DNA is diluted relative to relative to RNA (DNA in the ng μL^{-1} range) and also the polymerase operates at the diluted concentration. We thus expect that their effect on RNA nanostar phase separation and the viscoelastic properties of the RNA-rich phase is negligible. Any residual influence of such minor components on the onset of the instability is thus absorbed into the effective spinodal criterion rather than treated as a dominant material contribution.

1.14 Estimate of droplet volume fraction in the reaction

To assess whether the majority of transcription can plausibly occur in the dilute phase outside of the RNA droplets, we estimated the volume fraction of the sample occupied by the condensed (droplet) phase under standard reaction conditions. The estimate is based on the droplet size distribution and the droplet number density measured from confocal images acquired in the bulk solution (away from the glass surface) after transcription for WT droplets.

For each field of view, we recorded confocal images of the droplet population and determined (i) the number of droplets N in the field of view and (ii) their radii R_i. Droplet radii were obtained from the measured droplet areas A_i in the segmentation masks using

$$R_i = \sqrt{\frac{A_i}{\pi}}. \tag{18}$$

This conversion assumes approximately spherical droplets, consistent with the orthogonal views shown in the z-stack control (Supplementary Fig. 3). Assuming a spherical geometry, the condensed-phase volume contained in one field of view is

$$V_{\mathrm{drop}} = \sum_{i=1}^{N} \frac{4}{3}\pi R_i^3. \tag{19}$$

The total volume corresponding to the same field of view is given by

$$V_{\mathrm{tot}} = A_{\mathrm{FOV}}\, h, \tag{20}$$

where A_{FOV} is the imaged area in the xy ($640\,\mu\mathrm{m}$ x $640\,\mu\mathrm{m}$) plane and h is the effective channel height of $400\,\mu\mathrm{m}$.

The droplet volume fraction in a field of view is then

$$\phi = \frac{V_{\mathrm{drop}}}{V_{\mathrm{tot}}} = \frac{\sum_{i=1}^{N} \frac{4}{3}\pi R_i^3}{A_{\mathrm{FOV}}\, h}. \tag{21}$$

Applying this procedure to representative fields of view under standard conditions yields $\phi \approx 0.02$ (roughly 2% of the total volume is occupied by the dense phase).

Because the dilute phase constitutes the vast majority of the reaction volume (∼98%), it can dominate total RNA production even if a fraction of DNA templates is retained within droplets.

In addition, the RNA-rich dense phase is highly viscous, and transport of rNTPs and transcription components into the droplet interior is expected to be diffusion-limited, which can further reduce the effective transcription rate inside droplets compared to the dilute phase. Together, these considerations support the assumption used in the model that the dominant contribution to RNA production occurs outside droplets, while allowing that some transcription may also take place from templates retained within the condensed phase.

2 Estimates of kinetic parameters

In this section, we provide estimates for important kinetic parameters of the phase-separating nanostar system. These parameters are important for a quantitative comparison of experimental measurements to the theoretical model for vacuoles formation (see previous section), and thus confirm the underlying mechanism also by the numbers.

2.1 Transcription rate coefficient

We consider transcription of WT2 RNA nanostars composed of $L_{\text{WT2-RNA}}$ nucleotides. Experimentally, we recorded the fluorescence intensity F of WT2 RNA nanostars over time t, as shown in Supplementary Fig. 19a. Fluorescence intensity $F(t)$ is related to RNA number concentration $n_{\text{WT2-RNA}}$:

$$F(t) = \beta n_{\text{WT2-RNA}}(t)\,, \quad \text{with} \quad \beta = \frac{F(t\to\infty) L_{\text{WT2-RNA}}}{n_{\text{rNTP}}(t=0)}\,. \tag{22}$$

Here, β is the effective calibration constant estimated from the plateau fluorescence by assuming complete turnover and integration of the initially available nucleotides into WT2-RNA nanostars. At early times, the concentration of WT2 nanostars increases rapidly, while at later time, transcription slows down and eventually the WT2-RNA concentrations $n_{\text{WT2-RNA}}$ saturates; see Supplementary Fig 19b.

In the absence of phase separation, the transcription of nanostars (see also Eq. (6)) is governed by

$$\frac{\mathrm{d}n_{\text{WT2-RNA}}(t)}{\mathrm{d}t} = \tilde{k}\; n_{\text{WT2-DNA}} \frac{1}{\dfrac{K_\alpha}{n_{\text{rNTP}}(t)} + 1}\,, \tag{23}$$

leading to a decrease of rNTPs according to

$$\frac{\mathrm{d}n_{\text{rNTP}}(t)}{\mathrm{d}t} = -L_{\text{WT2-RNA}} \frac{\mathrm{d}n_{\text{WT2-RNA}}(t)}{\mathrm{d}t}. \tag{24}$$

Here, $\tilde{k}$ is the transcription rate constant corresponding to WT2-DNA. Moreover, K_α is the threshold nutrient concentration below which the T7 polymerase enters the nutrient depleted and thus transcription slows down. The goal in the following is to obtain the transcription rate $\tilde{k}$ and the threshold concentration K_α from fitting to experimental data. To this end, we follow a two-step procedure and consider the system at early times (i) and later times (ii).

(i) At early times, the nucleotide concentration is large compared to the threshold nutrient concentration $n_{\mathsf{rNTP}}(t) \gg K_\alpha$. In this limit, Eq. (23) reduces to

$$\frac{d\,n_{\mathsf{WT2\text{-}RNA}}(t)}{dt} \simeq \tilde{k}\,n_{\mathsf{WT2\text{-}DNA}} =: v_0\,, \tag{25}$$

where v_0 is the initial rate of WT2-RNA nanostar production. To obtain the transcription rate constant $\tilde{k}$, we fit the initial linear regime of the experimental data, corresponding to the first approximately two hours (see Supplementary Fig. 19b, inlet), and obtain $v_0 = 3.3 \times 10^{-3} \mu\mathrm{M/s}$. Following Eq. (25) and dividing v_0 by the number concentration of the DNA $n_{\mathsf{WT2\text{-}DNA}} = 17\,\mathrm{nM}$ used in the experiment, we get the transcription rate:

$$\tilde{k} = 0.193\,\mathrm{s}^{-1}\,. \tag{26}$$

(ii) To estimate K_α, we determine nucleotide concentration $n_{\mathsf{rNTP}}(t)$ from the measured WT2-RNA concentration $n_{\mathsf{WT2\text{-}RNA}}(t)$ using the conservation of the total amount of nucleotides:

$$n_{\mathsf{rNTP}}(t) = n_{\mathsf{rNTP}}(0) - L_{\mathsf{WT2\text{-}RNA}}\,n_{\mathsf{WT2\text{-}RNA}}(t)\,. \tag{27}$$

Using $n_{\mathsf{rNTP}}(t)$ from the above relation, we determine the production rate $d\,n_{\mathsf{WT2\text{-}RNA}}(t)/dt$ from the experimental time trace and plot it as a function of $n_{\mathsf{rNTP}}(t)$; see green dots in Supplementary Fig. 19d. Since the numerical derivative is sensitive to noise in the experimental data, we smooth the curve using a Savitzky–Golay filter, which locally fits the data with a low-order polynomial while approximately preserving the slope. From this analysis, we obtain the threshold nutrient rNTPs concentration when the T7 polymerase crosses over to the nutrient limited regime:

$$K_\alpha = 4.0\,\mathrm{mM}\,. \tag{28}$$

Finally, using the fitted values of $\tilde{k}$ and K_α, we solve Eqs. (23) and (24) to calculate $n_{\mathrm{WT2-RNA}}(t)$. The resulting theoretical curve is shown in Fig. 19b,c as the black dashed line, agreeing well with the experimental data.

We note that the transcription rate depends on the length of the specifically considered DNA template as the transcription machinery has to run over the DNA template to produce a complete RNA molecule. In other words, a longer template composed of more nucleotides thus leads to a smaller effective rate of producing full transcripts. For example, the rate of transcription for a WT RNA of length $L_{\mathsf{WT\text{-}RNA}}$, was determined using a slightly different DNA template of length $L_{\mathsf{WT\text{-}DNA}}$. If the speed of the transcription machinery per nucleotide is assumed to be unchanged, then the rate of producing complete transcripts scales inversely with the DNA-template length. Thus, the corresponding effective transcription rate for WT nanostar becomes

$$k = \tilde{k}\frac{L_{\mathsf{WT2\text{-}DNA}} - L_{\mathsf{prom}}}{L_{\mathsf{WT\text{-}DNA}} - L_{\mathsf{prom}}}\,. \tag{29}$$

Here, $L_{\mathsf{WT\text{-}DNA}}$ and $L_{\mathsf{WT2\text{-}DNA}}$ denote the total lengths of the WT and WT2 DNA templates, respectively, and L_{prom} is the promoter length. We subtract the promoter length because as it mainly facilitates the binding of T7 polymerase to initiate transcription reaction. It does not contribute to the length-dependent elongation of the RNA transcript.

2.2 Time when the nutrient rNTP gets depleted

To estimate the timescale over which the nucleotide pool is depleted, we combine Eqs. (23) and (24), and integrate it from the initial nucleotide concentration $n_{\mathrm{rNTP}}(0)$ to a final concentration $n_{\mathrm{rNTP}}(t)$, which results in time required for the nucleotide concentration to reach $n_{\mathrm{rNTP}}(t)$ as

$$t = \frac{1}{L_{\text{WT2-RNA}}\tilde{k}\, n_{\text{WT2-DNA}}} \left[n_{\text{rNTP}}(0) - n_{\text{rNTP}}(t) + K_\alpha \ln\left(\frac{n_{\text{rNTP}}(0)}{n_{\text{rNTP}}(t)} \right) \right] . \tag{30}$$

In the experiment, the initial rNTP concentration is $10\,\mathrm{mM}$, and the DNA concentration is $17\,\mathrm{nM}$. WT2 RNA nanostar is produced with a transcription rate $\tilde{k} = 0.195\,\mathrm{s}^{-1}$, and consists of $L_{\mathrm{WT2-RNA}} = 257$ nucleotides. The transcription reactions become strongly nutrient-limited when the number concentration of nutrients n_{rNTP} in the system approaches K_α (value see Eq. (28)). We therefore estimate the time when the T7 polymerase enters the nutrient rNTP limited regime of nucleotide limitation by setting $n_{\text{rNTP}}(t) = K_\alpha$ in Eq. (30):

$$t \approx 1.14 \times 10^4 \text{ s} \approx 3.17 \text{ h} . \tag{31}$$

For large time where $n_{\text{rNTP}}(t) \ll K_\alpha$, the nutrient decay scales linearly with nutrient concentration n_{rNTP}. The corresponding characteristic time scale is given as:

$$\tau = \frac{K_\alpha}{L_{\text{WT2-RNA}}\tilde{k}\, n_{\text{WT2-DNA}}} , \tag{32}$$

and the half-time is given as $t_{1/2} = \tau \ln(2)$. Inserting the DNA concentration, and $K_\alpha = 4.0\,\mathrm{mM}$ (Eq. (28)), we find for the characteristic nutrient decay time in the nutrient limited regime of the T7 polymerase:

$$\tau \approx 4.75 \times 10^3 \text{ s} \approx 1.32 \text{ h} . \tag{33}$$

2.3 Diffusivity inside

The slowest relaxation time as typically probed in a FRAP experiments is given as

$$\tau = \frac{R^2}{\pi D^{\text{in}}} + P\frac{R^2}{3D^{\text{out}}} , \tag{34}$$

where P is the partitioning coefficient of nanostars between the nanostar-rich and poor phase, and R is the radius of the considered nanostar-rich droplet in the FRAP experiment. Moreover, $D^{\text{in/out}}$ is the diffusivity in the nanostar-rich/poor phase, respectively. For slow diffusion inside, $D^{\text{in}} < D^{\text{out}}/P$ ($P \sim 10^2$, see phase diagram, main text figure 4), we can determine the diffusivity inside from the slowest FRAP recovery time τ:

$$D^{\text{in}} \simeq \frac{R^2}{\pi\tau} . \tag{35}$$

We performed FRAP experiments of nanostar-rich droplets with a radius of about $R \simeq 25\mu m$. Similar to previous works, we find that there is no significant recovery, even 10 minutes after bleaching (Supplementary Fig. 6).[6,12] Based on these estimates and previous work on RNA droplets[13] we estimate that recovery occurs roughly on a time-scale of half a day ($\tau \simeq 12\cdot 3600 s$). Thus, we find the diffusion coefficient inside the nanostar-rich droplet is $D^{\text{in}} \simeq 5 \cdot 10^{-3} \mu m^2/s$. This value is more 10^3 lower than the expected diffusion coefficient of the nanostar in the nanostar-poor phase, which is consistent with neglecting the second term in Eq. (35) for estimating the recovery time.

2.4 Viscosity inside

From the diffusivity inside, we can estimate the viscosity of the nanostar-rich droplet using the Sutherland-Einstein-Stokes relationship:

$$\eta = \frac{k_B T}{6\pi D^{\text{in}} a/2}\,, \tag{36}$$

where k_B is the Boltzmann constant, and a the nanostar diameter. Using the diffusion coefficient estimate obtained in the last section and $a \simeq 10nm$, we find a nanostar-droplet viscosity $\eta \simeq 1\,\mathrm{Pa\,s}$. This value is 10^3 times higher than the viscosity of water.

2.5 Capillary velocity, viscosity and surface tension

We experimentally estimated the capillary velocity from droplet fusion events: $v \simeq 10^{-2}\mu m/s$ (see Section 1.7). Using that the surface tension scales $\gamma \simeq v\,\eta$, we can estimate it from the viscosity obtained in the last section. We find that the nanostar droplet surface tension is about $\gamma \simeq 10^{-2}\mu N/m$, which is more 10^6 times lower than of the surface tension of the air-water interface.

3 Supplementary Tables

3.1 DNA sequences

Sample	DNA sequence
WT	**GGG CAT TCT AAT ACG ACT CAC TAT A**| GGA CCG TCA GAG GAC ACG GTT AAA AA G TCC TCA TTC GTG GCC GCA TCG CGA AAG CGG CCA CGG GAT TCT GTG CTG CAC ATT AGA GTC GCT GTA TGA CCC ATC GCG AAA GGG TCG TAC AGC GGC TCT AGT GTG CTC GCG TGG ATC CCG ACT GGC ACC ATC GCG AAA GGT GCC AGG TAA CGA ATG GAT CCA CGC GTC ACT CGT AGC ATT GTG CCT GTC TCC ATC GCG AAA GGA GAT AGG CAC AGT G CTA TGA GTG TGC ACG GAA TCC
WT2	**GGG CAT TCT AAT ACG ACT CAC TAT A**| GGA CAG TGC TAT GAG TGT CGC GAC GGA GAC GGT CGG GTC CAG ATA GGC CAT CGC GAA AGG TCT ATC TGT CGA GTA GAG TGT GGG CTC CGT CGC GTG CAC ATT AGA GTC GCT GTA TGC CAC ATC GCG AAA GTG GCG TAC AGC GGC TCT AGT GTG CTG CAC AGT GTC TGT GCG ACT GCA CCC ATC GCG AAA GGG TGT AGT CGC ATA GAC ATT GTG CTC ACT CGT AGC ATT GTC CCT GTC TCC ATC GCG AAA GGA GAT AG
2KL mutant	**GGG CAT TCT AAT ACG ACT CAC TAT A**| GGG CCG TCA GAG GAC ACG GTT GAG AGG TCC TCG TTC GTG GTC GCG TCG CGA AAG CGG TCA CGG GAT TTT GTG CTG CAC ATT AGA GTC GCT GTA TGA CCC ATC GCG AAA GGG TCG TAC AGC GGC TCT AGT GTG CTC GCG TGG ATC CCG ACT GGC ACC ATC GCG AAA GGT GCC AGG TAA CGA ATG GAT CCA CGC GTT GCA CGG AAT CCC GTG GTC GCT TTC GCG GTG CGG CCA CGA ATG AGG ACT TTT TAA CTG TGT TCT CTG GTG GTT C
1KL mutant	**GGG CAT TCT AAT ACG ACT CAC TAT A**| GGG CCG TCA GAG GAC ACG GTT GAG AGG TCC TCG TTC GTG GTC GCG TCG CGA AAG CGG TCA CGG GAT TTT GTG CTG GAT CCC GAC TGG CAC CAT CGC GAA AGG TGC CAG GTA ACG GGA TCC AGC ACG GAA TCC CGT GGT CGC TTT CGC GGT GCG GCC ACG AAT GAG GAC TTT TTA ACT GTG TTC TCT GGT GGT TC
Mut RNA with BrA	**GGG CAT TCT AAT ACG ACT CAC TAT A**| GGG CCG TCA GAG GAC ACG GTT GAG AGG TCC TCG TTC GTG GTC GCG TCG CGA AAG CGG TCA CGG GAT TTT GTG CTG CAC ATT AGA GTC GCT GTA TGA CCC ATC GCG AAA GGG TCG TAC AGC GGC TCT AGT GTG CTC GCG TGG GGA GAC GGT CGG GTC CAG GCA CCA TCG CGA AAG GTG CCT GTC GAG TAG AGT GTG GGC TCC CCA CGC GTC ACT CGT AGC ATT GTG CCT GTC TCC ATC GCG AAA GGA GAT AGG CAC AGT GCT ATG AGT GTG CAC GGA ATC CCG TGG TCG CTT TCG CGG TGC GGC CAC GAA TGA GGA CTT TTT AAC TGT GTT CTC TGG TGG TTC

WT with tetraloops	**GGG CAT TCT AAT ACG ACT CAC TAT A**| GGA CCG TCA GAG GAC ACG GTT AAA AAG TCC TCA TTC GTG GCC GCG CTT GCG GCC ACG GGA TTC TGT GCT GCA CAT TAG AGT CGC TGT ATG ACC CGC TTG GGT CGT ACA GCG GCT CTA GTG TGC TCG CGT GGA TCC CGA CTG GCA CCG CTT GGT GCC AGG TAA CGA ATG GAT CCA CGC GTC ACT CGT AGC ATT GTG CCT GTC TCC GCT TGG AGA TAG GCA CAG TGC TAT GAG TGT GCA CGG AAT CC
Mut RNA with BrA and tetraloops	**GGG CAT TCT AAT ACG ACT CAC TAT A**| GGG CCG TCA GAG GAC ACG GTT GAG AGG TCC TCG TTC GTG GTC GCG TCG CGA AAG CGG TCA CGG GAT TTT GTG CTG CAC ATT AGA GTC GCT GTA TGA CCC GCT TGG GTC GTA CAG CGG CTC TAG TGT GCT CGC GTG GGG AGA CGG TCG GGT CCA GGC ACC GCT TGG TGC CTG TCG AGT AGA GTG TGG GCT CCC CAC GCG TCA CTC GTA GCA TTG TGC CTG TCT CCG CTT GGA GAT AGG CAC AGT GCT ATG AGT GTG CAC GGA ATC CCG TGG TCG CTT TCG CGG TGC GGC CAC GAA TGA GGA CTT TTT AAC TGT GTT CTC TGG TGG TTC

Supplementary Table 1: **Sequences of all DNA templates used in this study**. All sequences are written in 5'-3' direction. Bold letters denote the promoter regions. Vertical lines denote where transcription starts.

Direction	DNA sequence	Used for
forward	GGG CAT TCT AAT ACG ACT CAC TAT A	all templates
reverse	GGA TTC CGT GCA CAC TCA TA	WT
reverse	CTA TCT CCT TTC GCG ATG GA	WT2

Supplementary Table 2: **Sequences of the PCR primers**. All sequences are written in 5'-3' direction.

Sample	DNA sequence	% of reads
Second most abundant sequence in Mut DNA (mutant 1)	**GGG CAT TCT AAT ACG ACT CAC TATA** | GGA CCG TCA GAG GAC ACG GTT AAA AAG TCC TCA TTC GTG GCC GCA TCG CGA AAG CGG CCA CGG GAT TCT GTG CTG CAC ATT AGA GTC GCT GTA TGA CCC ATC GCG AAA GGG TCG TAC AGC GGC TCT AGT GTG CTC GCG TGG ATC CCG ACT GGC ACC ATC GCG AAA GGT GCC AGG TAA CGA ATG GAT CCA CGC GTC ACT CGT AGC ATT GTG CCT GTC TCC ATC GCG AAA GGA GAT AGG CAC AGT GCT ATG AGT GTG CAC GGA ATC CCG TGG CCG CTT TCG CGA TGC GGC CAC GAA TGA GGA CTT TTT AAC CGT GTC CTC TGA CGG TCC TAT AGT GAG TCG TAT TAG AAT GCC CA	2.65% (378bp long)
Third most abundant sequence in Mut DNA (mutant 2)	**GGG CAT TCT AAT ACG ACT CAC TATA** | GGA CCG TCA GAGG ACA CGG TTA AAA AGT CCT CAT TCG TGG CCG CAT CGC GAA AGC GGC CAC GGG ATT CCG TGC ACA CTC ATA GCA CTG TGC CTA TCT CCT TTC GCG ATG GAG ACA GGC ACA ATG CTA CGA GTG ACG CGT GGA TCC ATT CGT TAC CTG GCA CCT TTC GCG ATG GTG CCA GT	1.25% (205bp long)
Fourth most abundant sequence in Mut DNA	**GGG CAT TCT AAT ACG ACT CAC TATA** | GGA CCG TCA GAG GAC ACG GTT AAA AAG TCC TCA TTC GTG GCC GCA TCG CGA AAG CGG CCA CGG GAT TCT GTG CTG CAC ATT AGA GTC GCT GTA TGA CCC ATC GCG AAA GGG TCG TAC AGC GGC TCT AGT GTG CTC GCG TGG ATC CCG ACT GGC ACC ATC GCG AAA GGT GCC AGG TAA CGA ATG GAT CCA CGC GTC ACT CGT AGC ATT GTG CCT GTC TCC ATC GCG AAA GGA GAT AGG CAC AGT GCT ATG AGT GAC GCG TGG ATC CAT TCG TTA CCT GGC ACC TTT CGC GAT GGT GCC AGT CGG GAT CCA CGC GAG CAC ACT AGA GCC GCT GTA CGA CCC TTT CGC GAT GGG TCA TAC AGC GAC TCT AAT GTG CAG CAC AGA ATC CCG TGG CCG CTT TCG CGA TGC GGC CAC GAA TGA GGA CTT TTT AAC CGT GTC CTC TGA CGG TCC TAT AGT GAG TCG TAT TAG AAT GCC CA	0.75% (498bp long)

Fifth most abundant sequence in Mut DNA	**GGG CAT TCT AAT ACG ACT CAC TATA** | GGA CCG TCA GAG GAC ACG GTT AAA AAG TCC TCA TTC GTG GCC GCA TCG CGA AAG CGG CCA CGG GAT TCT GTG CTG CAC ATT AGA GTC GCT GTA TGA CCC ATC GCG AAA GGG TCG TAC AGC GGC TCT AGT GTG CTC GCG TGG ATC CCG ACT GGC ACC ATC GCG AAA GGT GCC AGG TAA CGA ATG GAT CCA CGC GTC ACT CGT AGC ATT GTG CCT GTC TCC ATC GCG AAA GGA GAT AGG CAC AGT GCT ATG AGT GTG CAC GGA ATC CCG TGG CCG CTT TCG CGA TGC GGC CAC GGG ATT CCG TGC ACA CTC ATC ACG CTG TGC CTA TCT CCT TTC GCG ATG GAG ACA GGC ACA ATG CTA CGA GTG ACG CGT GGA TCC ATT CGT TAC CTG GCA CCT TTC GCG ATG GTG CCA GTC GGG ATC CAC GCG AGC ACA CTA GAG CCG CTG TAC GAC CCT TTC GCG ATG GGT CAT ACA GCG ACT CTA ATG TGC AGC ACA GAA TCC CGT GGC CGC TTT CGC GAT GCG GCC ACG AAT GAG GAC TTT TTA ACC GTG TCC TCT GAC GGT CCT ATA GTG AGT CGT ATT AGA ATG CCC A	0.35% (608bp long)

Supplementary Table 3: **Most common sequences in the Mut sequencing data**. All sequences are written in 5'-3' direction. The most common sequence representing 94.66% of all reads is the WT sequences shown in Supplementary Table 1. Bold letters denote the promoter regions. Vertical lines denote where transcription starts. The predicted RNA structures for Mutant 1 and Mutant 2 sequences are shown in Supplementary Fig. 14b.

3.2 Phase separation experiments and model calibration

Condition	Starting conc.		Estimated conc. (WT)		Estimated conc. (Mut)	
	WT	Mut	Dilute	Condensed	Dilute	Condensed
1	542.08	0.00	27.48	12 893		
2	0.00	382.18			206	
3	1 339.47	483.17	53.68	12 805	0.53*	4 787
4	1 301.24	617.35	92.98	48 826	115.63	20 352

Supplementary Table 4: Starting mass concentrations and estimated post-partition mass concentrations in the dilute and condensed phases for wild-type (WT) and mutant (Mut) RNA nanostars (Fig. 4b). Estimates were obtained from the calibration curve in Supplementary Fig. 16. Values marked with an asterisk (*) fall outside the calibration range. All values are in $\mathrm{ng}/\mu\mathrm{L}$ units.

Parameter	Symbol	value	Unit
Mol. vol. (WT)	ν_{WT}	257.69	$\mathrm{L\,mol^{-1}}$
Mol. vol. (Mut)	ν_{Mut}	386.53	$\mathrm{L\,mol^{-1}}$
Interaction (WT–WT)	$e_{\text{WT,WT}}$	-0.5145	$k_{\text{B}}T$
Interaction (Mut–Mut)	$e_{\text{Mut,Mut}}$	-0.4888	$k_{\text{B}}T$
Interaction (WT–Mut)	$e_{\text{WT,Mut}}$	-0.5242	$k_{\text{B}}T$

Supplementary Table 5: From the fitting of the experimental phase diagram (Fig. 4b in the main text), using Eq. (5), we obtained the parameters listed above. Specifically, the fits give the molecular volumes of the WT and Mut nanostars, and the interaction parameters among all nanostars components. We neglect all the interactions with solvent, i.e, ($e_{\text{WT,sol}} = e_{\text{Mut,sol}} = e_{\text{sol, sol}} \simeq 0$). All energy values in the table are given in units of k_BT where k_{B} is the Boltzmann constant and T is the temperature.

Parameter	Symbol	value	units
Initial rNTPs conc.	$n_{\text{rNTP}}(0)$	10	mM
WT2 DNA conc.	$n_{\text{WT2-DNA}}$	17	nM
WT2 DNA length	$L_{\text{WT2-DNA}}$	282	bp
WT2 RNA length	$L_{\text{WT2-RNA}}$	257	nts
Maximum production rate	$v_{\max}$	3.3×10^{-3}	$\mu\mathrm{M\,s^{-1}}$
Transcription rate	$\tilde{k}$	0.193	$\mathrm{s^{-1}}$
threshold nutrient conc.	K_α	4.01	mM
Fluorescence calibration constant	β	185.4	$\mathrm{A.U.\,nt}\,\mu\mathrm{M^{-1}}$

Supplementary Table 6: Summary of the parameters used and quantities estimated from fitting the experimental data obtained from WT2 transcription reactions described in Sec 2.1 and illustrated in Supplementary Fig. 19.

Parameter	Symbol	value	units
Diffusion (Mut-Rich phase)	$D^{\text{in}}_{\text{Mut}}$	10^{-2}	$\mu\text{m}^2\,\text{s}^{-1}$
Diffusion (WT-Rich phase)	$D^{\text{in}}_{\text{WT}}$	10^{-4}	$\mu\text{m}^2\,\text{s}^{-1}$
Transcription rate	k	0.187	s^{-1}
Initial radius	$R_0(t=\tilde{t}\,)$	1	μm
Initial rNTPs conc.	$n^{\text{out}}_{\text{rNTPs}}(t=\tilde{t}\,)$	10	mM
threshold nutrient conc.	K_α	4.01	mM
mol. vol. rNTPs	ν_{rNTP}	0.5	$\text{L}\,\text{mol}^{-1}$
WT DNA conc.	$n^{\text{out}}_{\text{WT-DNA}}$	15.4	nM
WT DNA length	$L_{\text{WT-DNA}}$	290	bp
Mut DNA length	$L_{\text{Mut-DNA}}$	367	bp
WT RNA length	$L_{\text{WT-RNA}}$	265	nt
Mut RNA length	$L_{\text{Mut-RNA}}$	342	nt

Supplementary Table 7: The parameters used for the kinetics on the Narrow interface model for the vacuole formation.

4 Supplementary Figures

4.1 Agarose gel electrophoresis (AGE) of WT DNA and WT2 DNA before and after PCR amplification

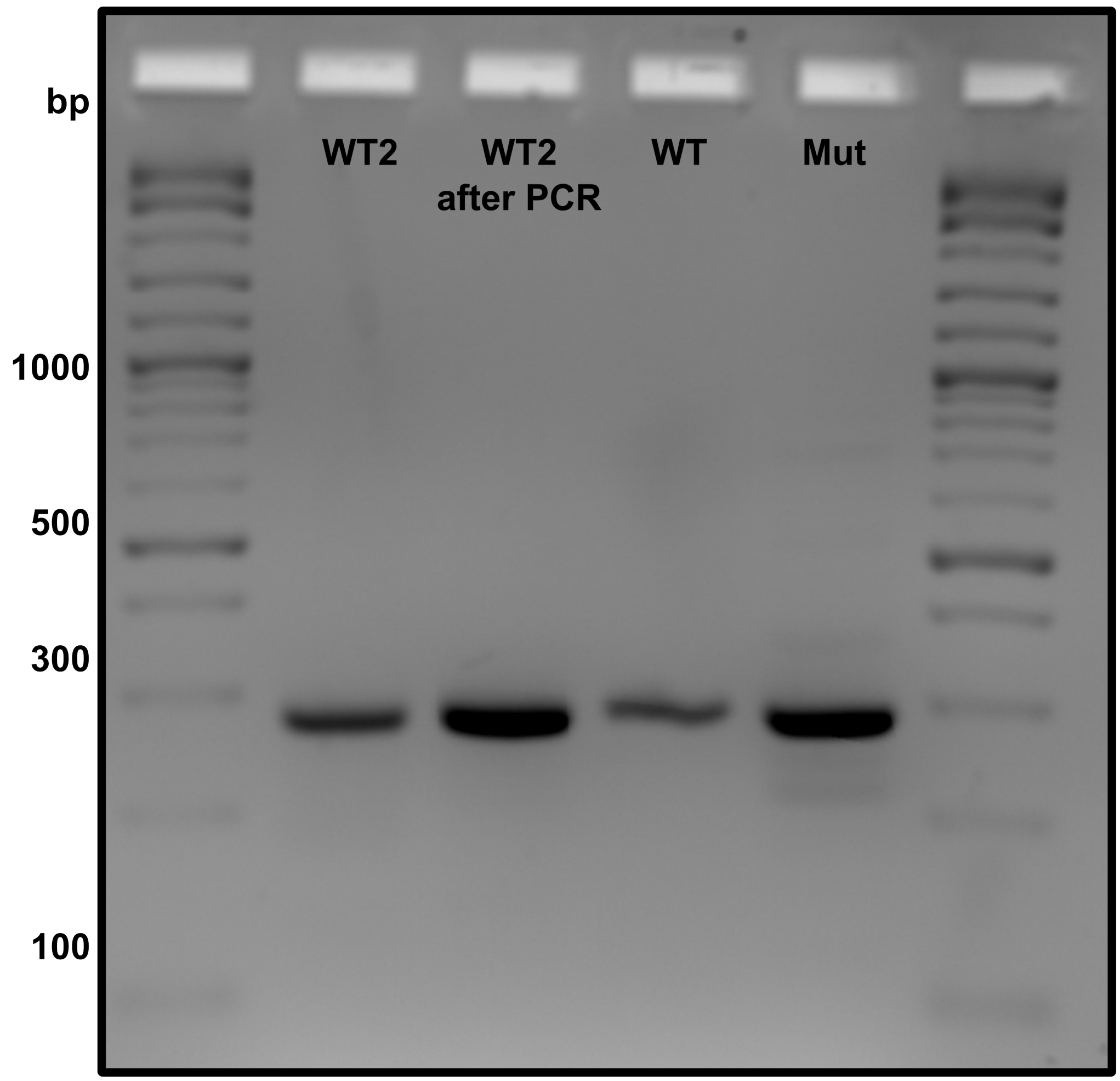

Supplementary Fig. 1: **3% AGE of the WT DNA and WT2 DNA before and after PCR amplification (Mut is WT DNA after PCR amplification).** The length of the DNAs present in the ladder (NEB, TriDye™ 1 kb Plus DNA Ladder, Cat No. N3270S) on both sides of the gel is reported on left side in base pair (bp). Visual inspection of the gel confirms that amplification of WT DNA results in multiple unspecific bands whereas amplification of WT2 DNA does not.

4.2 Mechanism for non-specific PCR product formation

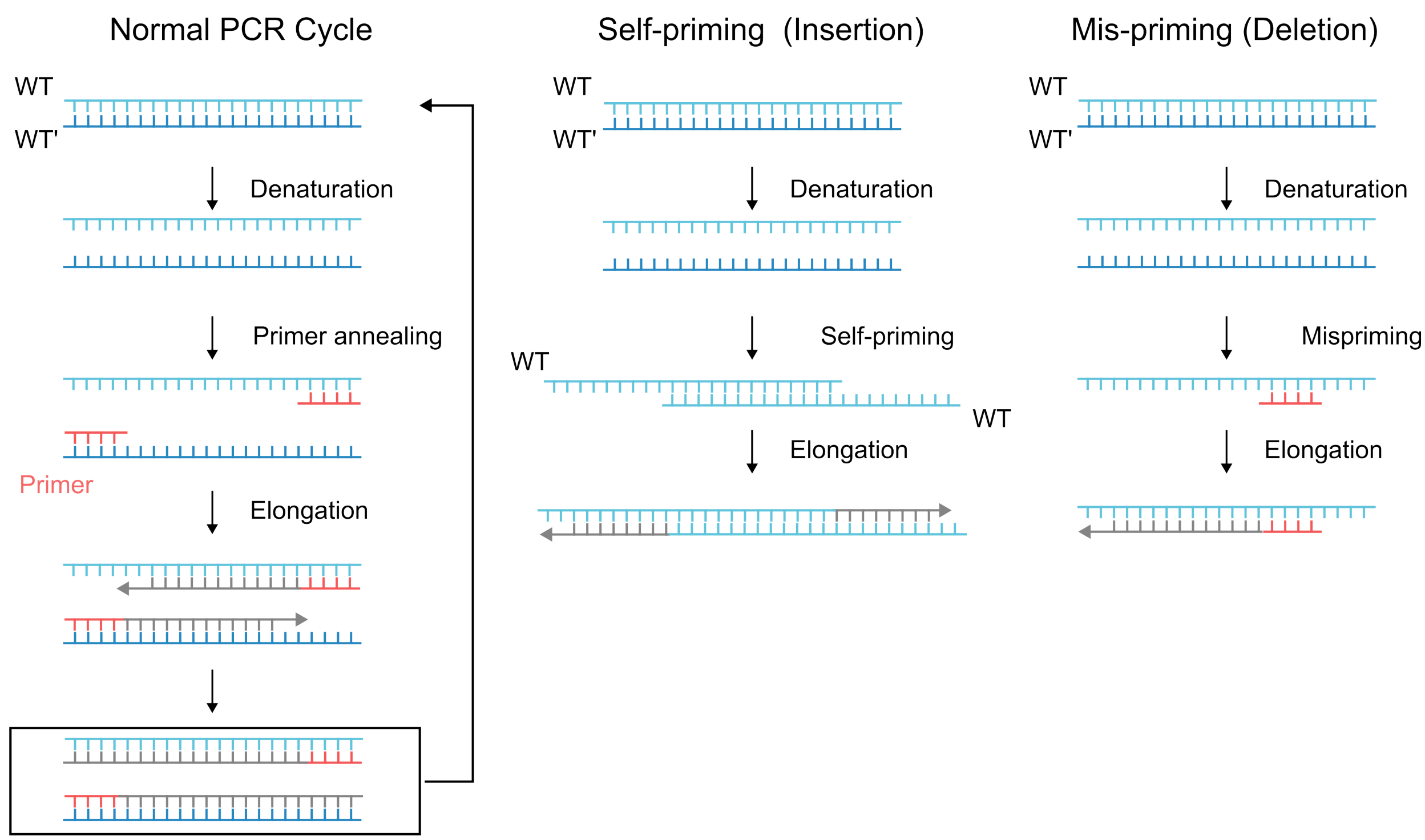

Supplementary Fig. 2: **Schematic showing how non-specific PCR products can arise during amplification of the WT DNA template.** PCR proceeds through repeated cycles of denaturation (strand separation), annealing, and extension. After denaturation, the annealing step can occur via the intended binding of the forward and reverse primers to the template ends (left), followed by polymerase extension to yield the expected full-length amplicon. Alternatively, because the WT template contains self-complementary regions, partially annealed template strands can act as primers for one another (intermolecular self-priming; middle), and subsequent extension generates longer, duplicated/extended products. In addition, primers can anneal non-specifically to the DNA with partial complementarity (mis-priming; right), leading to extension from an incorrect start site and thus truncated (shorter) amplicons. Red segments indicate primers; grey arrows indicate polymerase extension; dotted regions indicate imperfect/partial base pairing.

4.3 Confocal z-stack of WT RNA droplets

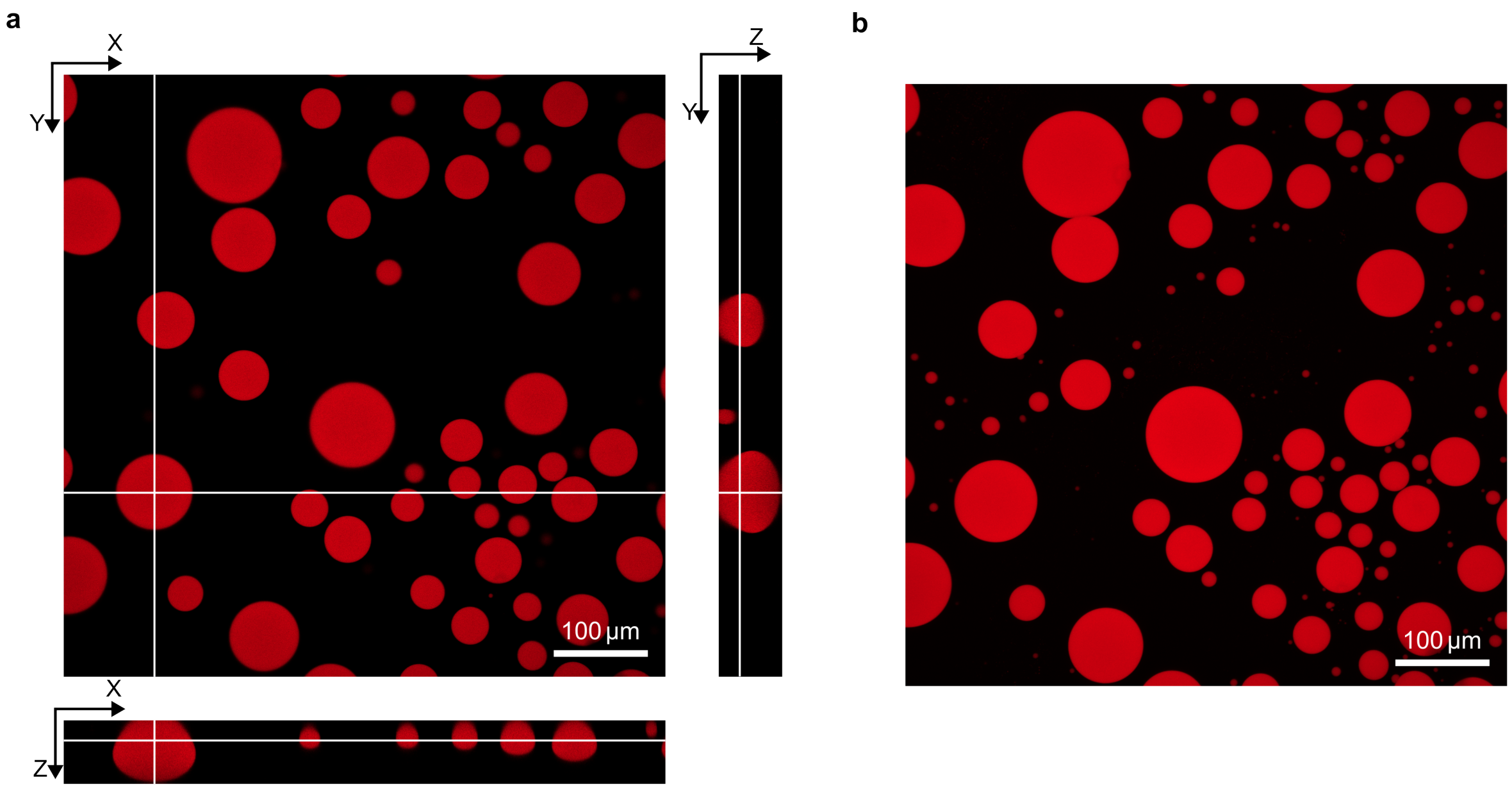

Supplementary Fig. 3: **Confocal z-stack to assess droplet wetting.** **a** Representative confocal z-stack of WT RNA droplets (red), droplets contain malachite green aptamer and are labelled with malachite green dye (λ_{ex}= $640\,\mathrm{nm}$). The orthogonal views XZ (bottom) and YZ (right) are shown to visualise the droplet shape relative to the glass surface. The first frame was taken on the glass surface (z=0). The droplets are predominantly spherical, with only mild flattening consistent with gravitational settling rather than strong surface wetting/spreading. White cross-hairs indicate the position of the orthogonal slices. The stack spans a total height of $67.54\,\mu\mathrm{m}$ and contains 98 slices; the representative XY view shown corresponds to slice 33/98. **b** Maximum-intensity projection of the confocal z-stack shown in **a**. Images are representative of two independent experiments with similar results. Scale bars, $100\,\mu\mathrm{m}$.

4.4 Violin plots of the droplet area for Mut and WT droplets

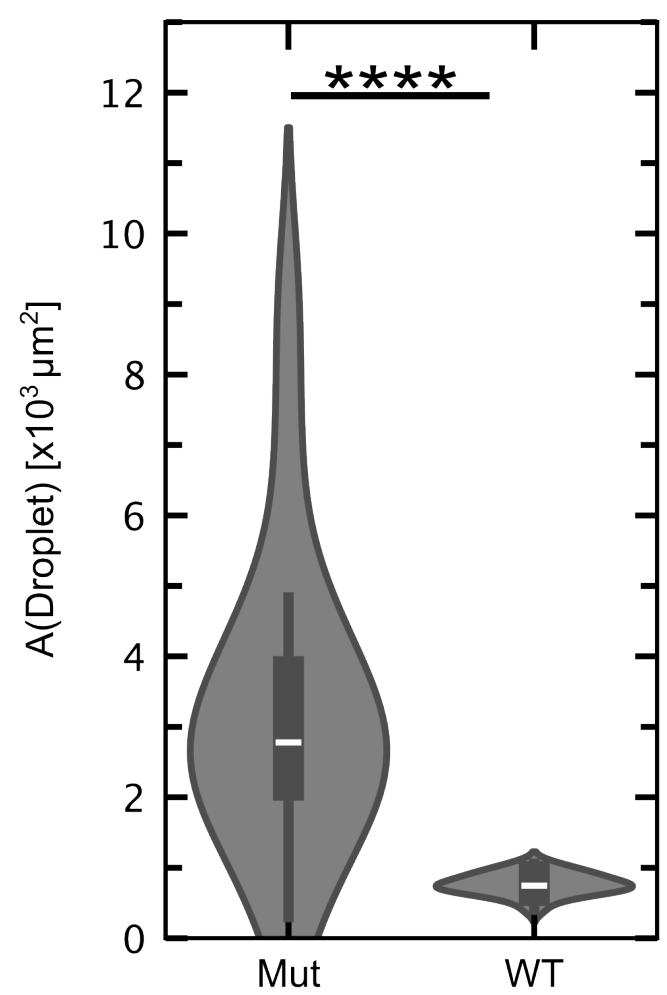

Supplementary Fig. 4: **Violin plots of the droplet area for Mut and WT droplets as measured from confocal snapshots.** Statistics: median test: significance with p-values $<$ 0.001 are marked with **** ($P = 2.3 \times 10^{-5}$). Median is shown in white, box edges denote the lower and upper quartiles, and whiskers extend to the most extreme data points within 1.5× the interquartile range. Droplets are grouped per field of view ($n_{Mut} = 18$, $n_{WT} = 23$), from 3 independent replicates.

4.5 Percentage of vacuolated droplets from Mut DNA, WT DNA and PCR-amplified WT DNA without reverse primers

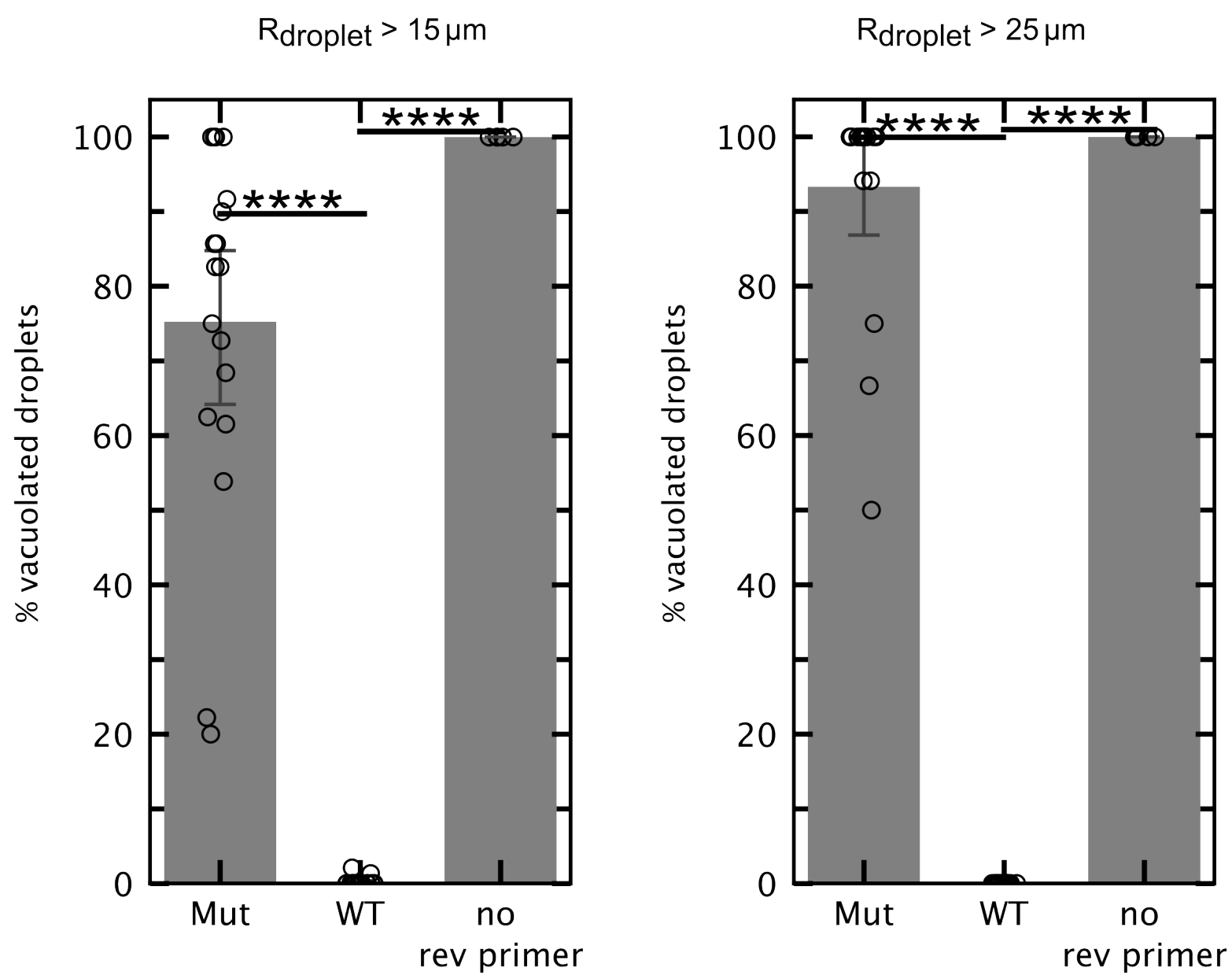

Supplementary Fig. 5: **Quantification of the percentage of vacuolated droplets from Mut DNA, WT DNA and PCR-amplified WT DNA without reverse primers.** We find that the larger droplets are more prone to vacuolation. A droplet is vacuolated if at least 5% of its area is hollow (see Data and code availability for more details). Statistics: median test: significance with p-values < 0.001 are marked with ****. Droplets are grouped per field of view (left graph: $n_1 = 18, n_2 = 23, n_3 = 5$ and right graph: $n_{Mut} = 18, n_{WT} = 17, n_{-revprimer} = 5$), from at least 2 independent replicates. The error bars denote the 95% confidence interval. For the left graph the p-values are: $P = 4.0 \times 10^{-7}$ (Mut VS WT) and $P = 0.0002$ (WT VS No reverse primer) and for the right graph the p-values are: $P = 1.5 \times 10^{-7}$ (Mut VS WT) and $P = 4.4 \times 10^{-5}$ (WT VS No reverse primer).

4.6 Quantification of the material properties of RNA droplets

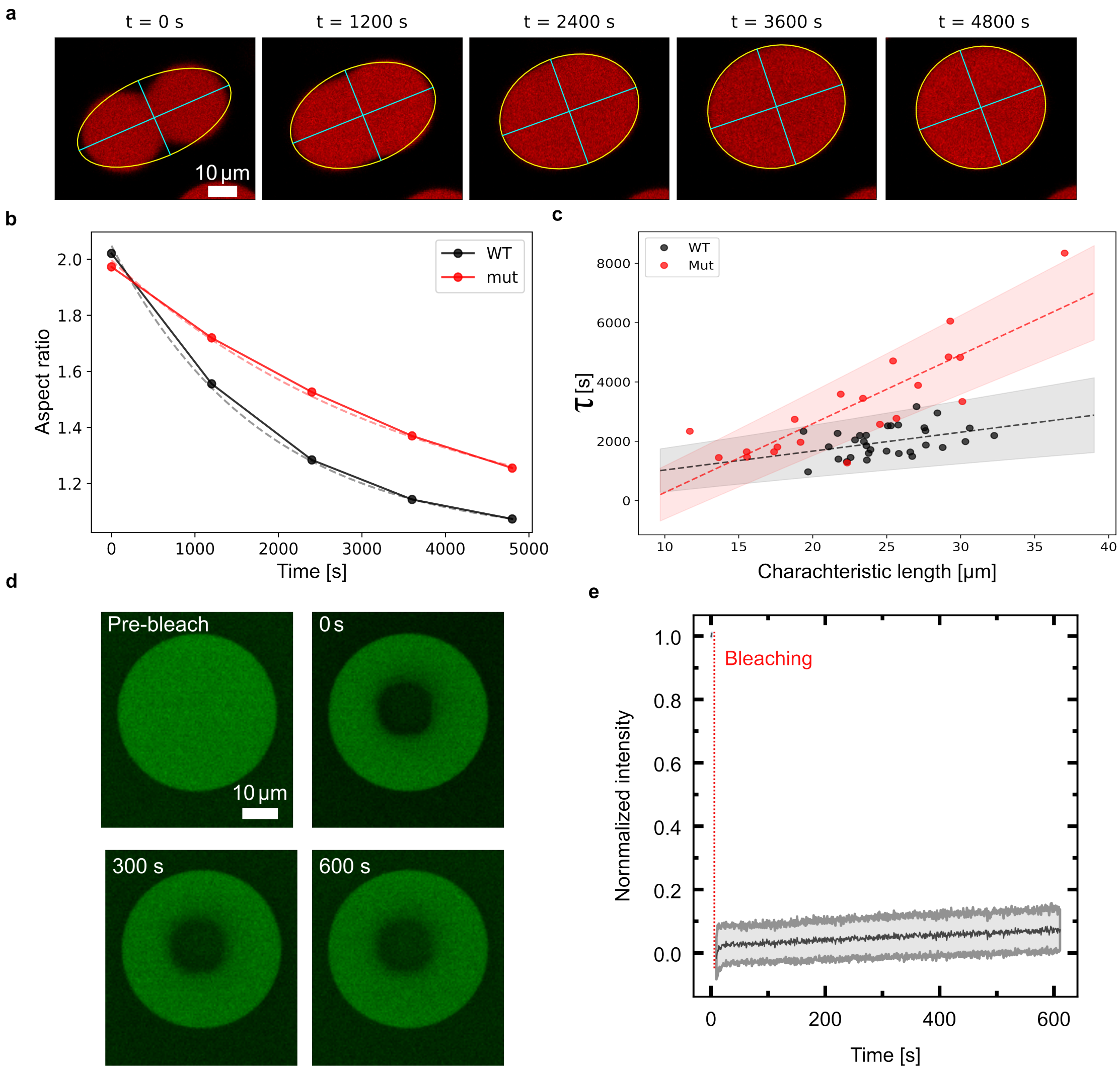

Supplementary Fig. 6: **Quantification of the material properties of RNA droplets. a** Schematic illustration of the relaxation dynamics of RNA droplets used to quantify the capillary velocity. $v = \frac{\gamma}{\eta}$. **b** Example of the measured aspect ratio over time for a WT (black) and Mut (red) droplet. **c** Fitted inverse capillary velocity for Mut (red) and WT (black) droplets. Inverse capillary velocity for WT (2 replicates, N=31), $v^{-1} = 63.70 \pm 27.12\,\mathrm{s}\,\mu\mathrm{m}^{-1}$ and for Mut (2 replicates, N = 20), $v^{-1} = 231.96 \pm 34.85\,\mathrm{s}\,\mu\mathrm{m}^{-1}$ (± s.d.). Hence for both condensates, $v \sim 10^{-2}\,\mu\mathrm{m}\,\mathrm{s}^{-1}$ **d** Example of WT RNA droplet before and after bleaching, showing negligible recovery even after 10 minutes. Scale bar, $10\,\mu\mathrm{m}$. **e** Quantification of the fluorescence recovery for WT droplets (n = 2 from a single experiment, T = $37\,^{\circ}\mathrm{C}$). Data was normalised to intensity before bleaching and background to account for background fluorescein signal. The black line denotes the mean and the light gray area represents the standard deviation.

4.7 Co-localisation of DNA templates with RNA droplets

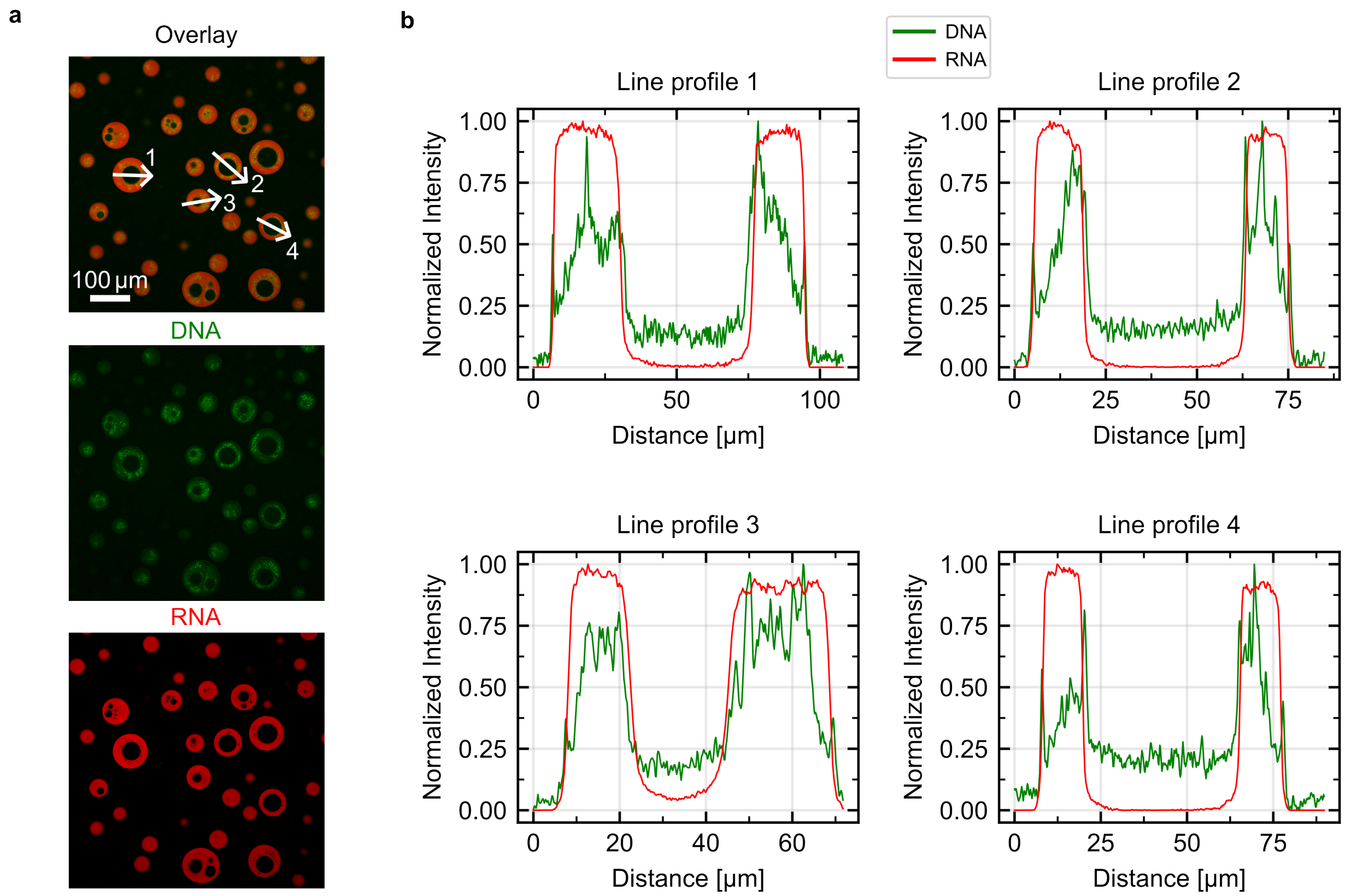

Supplementary Fig. 7: **Co-localisation of DNA templates with RNA droplets.** **a** Representative confocal images of RNA droplets (red) formed by in vitro transcription from fluorescently labelled DNA templates (green). RNA droplets contain the malachite green aptamer and are labelled with malachite green dye (λ_{ex}=$640\,\mathrm{nm}$); DNA templates are labelled with fluorescein-dUTP (λ_{ex}=$488\,\mathrm{nm}$). Overlay shows co-localisation of DNA with the RNA-rich condensates, consistent with encapsulation/retention of the DNA template during co-transcriptional droplet formation. Scale bar, $100\,\mu\mathrm{m}$. **b** Line profile analysis of DNA fluorescence relative to the RNA fluorescence signal across droplets confirms that the DNA signal is localised within the condensate interior and does not show systematic enrichment at the interface of vacuolated droplets. We also cannot observe DNA templates inside of the vacuoles (although this may be due to low signal when the aggregates are dissolved in bulk solution). Experiment was performed twice independently with similar results.

4.8 Transcription of Mut DNA with RNase inhibitors

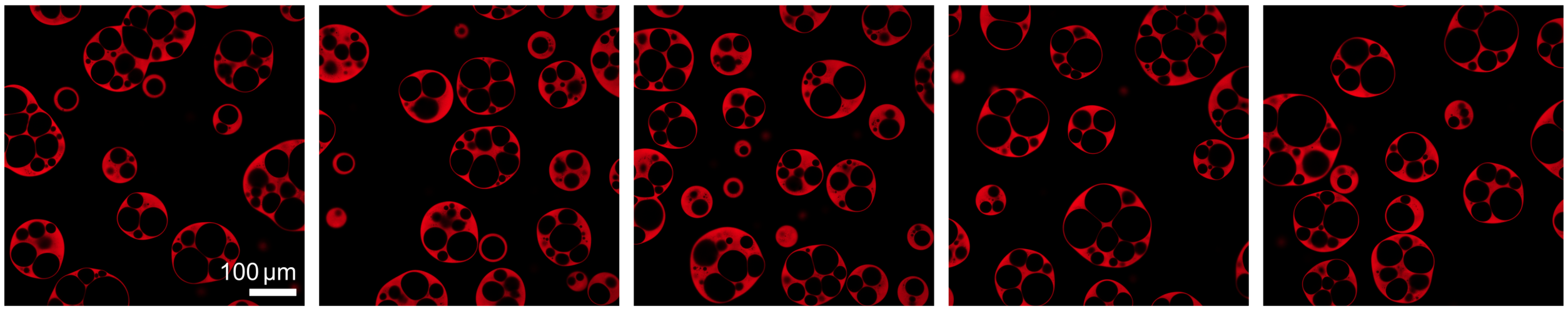

Supplementary Fig. 8: **Confocal snapshots of Mut RNA droplets transcribed with RNase inhibitors.** Mut RNA was transcribed and imaged after 48 hours of transcription at $37\,^{\circ}\mathrm{C}$. RNA droplets are shown in red, with MGA labelled with malachite green dye, $\lambda_{ex} = 640\,\mathrm{nm}$. Droplets are transcribed and imaged as described in Methods section, with $1\,\mu\mathrm{L}$ of RNase inhibitor (ScriptGuard™, Cat. No. C-SRI6310K) in $40\,\mu\mathrm{L}$ sample. Images represent 5 fields of view from a single experiment. Scale bar represents $100\,\mu\mathrm{m}$.

4.9 Mut and WT RNA droplets after 13 days of incubation at $37\,^{\circ}\mathrm{C}$

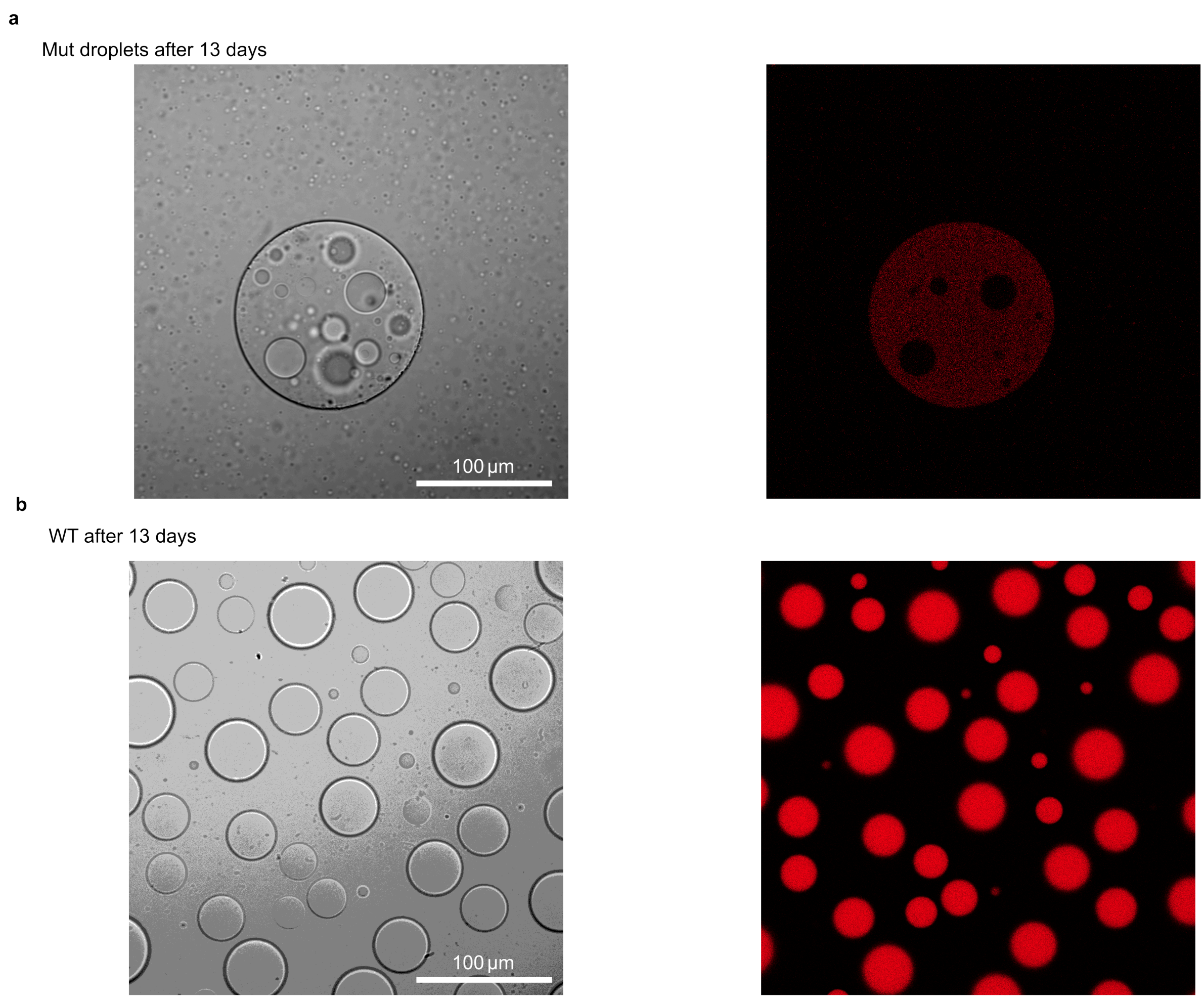

Supplementary Fig. 9: **Confocal snapshots of Mut and WT RNA droplets after 13 days of incubation at $37\,^{\circ}\mathrm{C}$.** Both samples are incubated on the same slide at $37\,^{\circ}\mathrm{C}$ for 13 days. RNA droplets are shown in red (right), labelled with the malachite green aptamer (MGA) and malachite green dye, $\lambda_{ex} = 640\,\mathrm{nm}$. Bright-field images are also shown (left) since both samples are heavily photo-bleached after 13 days. Scale bars, $100\,\mathrm{\mu m}$. Images show representative confocal micrographs from a single experiment.

4.10 RNA droplets transcribed from gel-purified long and short Mut DNA sequences

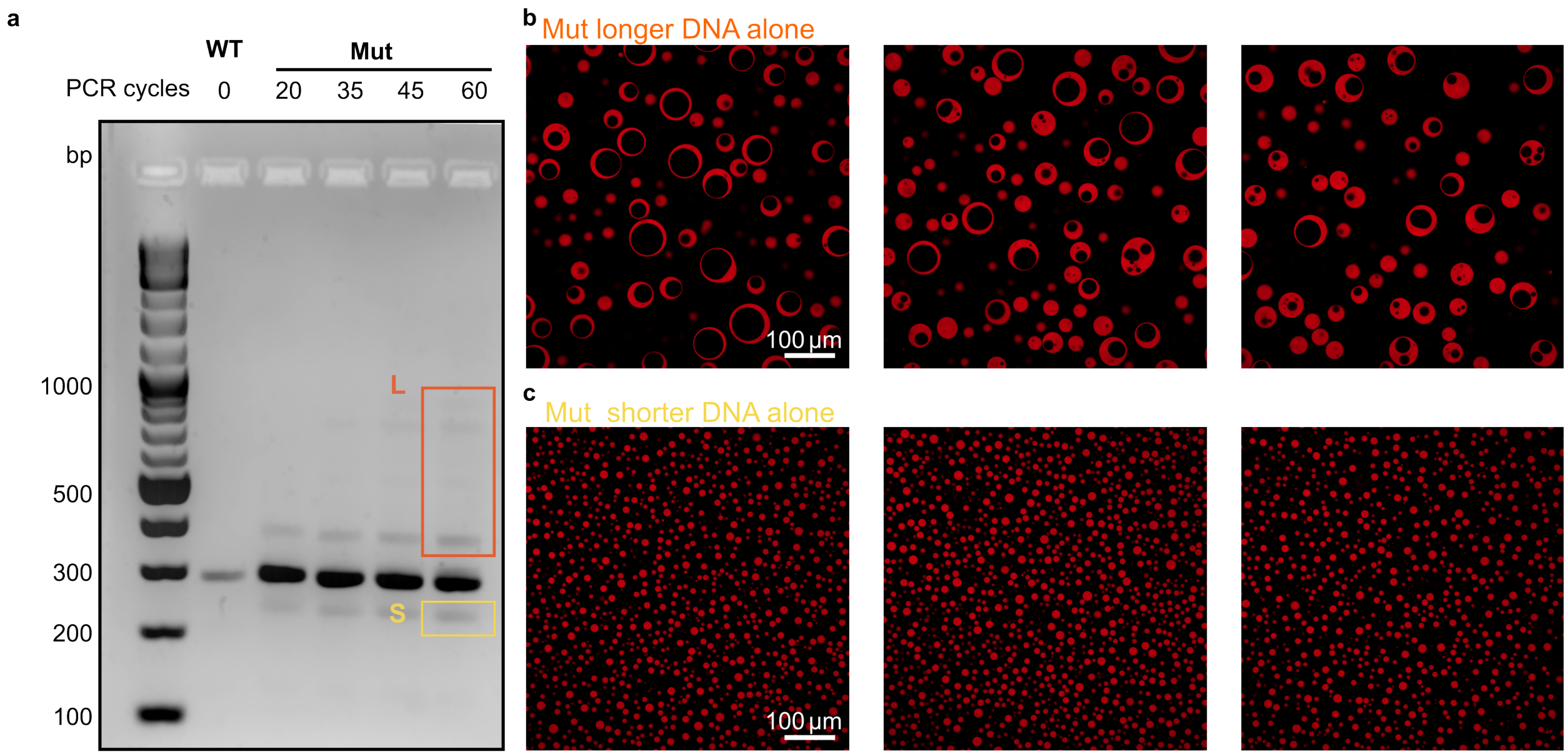

Supplementary Fig. 10: **RNA droplets transcribed from gel-purified long (L) and short (S) Mut DNA sequences. a** 3 % AGE gel showing which lanes are purified and then transcribed. DNA is purified in the L (**b**) and S (**c**) regions with no main band. The same gel is shown in Fig. 2a in the main text. **b** The purified DNA is transcribed and imaged after 48 hours of transcription at $37\,°\mathrm{C}$. RNA droplets are shown in red, with MGA labelled with malachite green dye, $\lambda_{ex} = 640\,\mathrm{nm}$. Note that in Fig. 2 in the main text, droplets come from the RNA transcribed when purifying the main band together with the longer DNA bands (M+L), or the main band together with the shorter DNA bands (M+S) (Fig. 2b). Here only the longer or only the shorter DNA bands are transcribed. For **b**-**c** experiments were reproduced at least twice independently with similar results. Scale bars represent $100\,\mathrm{\mu m}$.

4.11 PCR products and RNA droplets resulting from PCR-amplified WT DNA with and without different sets of primers

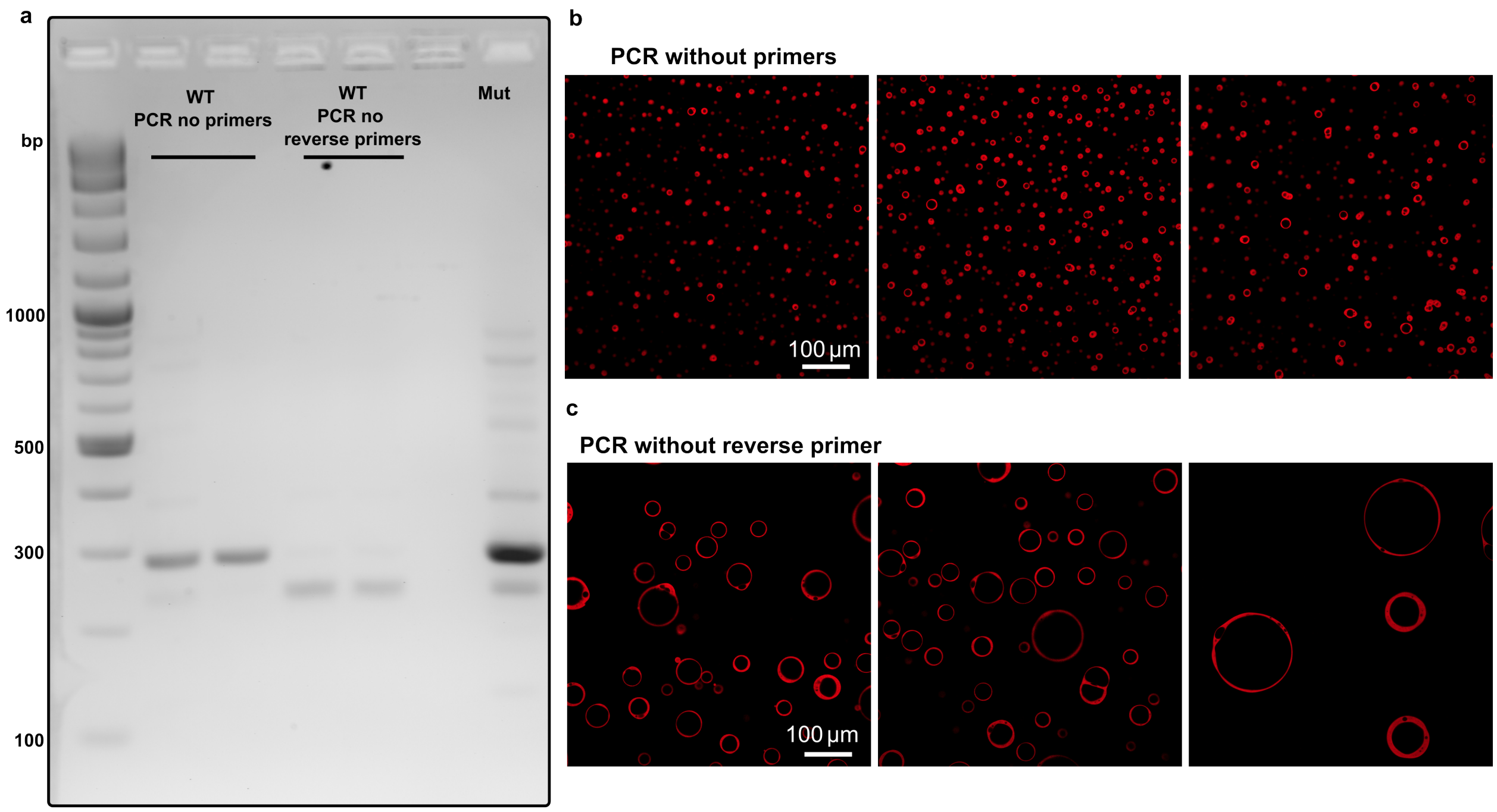

Supplementary Fig. 11: **PCR products and RNA droplets resulting from PCR-amplified WT DNA with and without different sets of primers. a** 3% AGE of the PCR-amplified WT DNA with and without different sets of primers. The length of the DNAs present in the ladder (NEB, 1 kb Plus DNA Ladder, Cat No. N3200S) on left side of the gel is reported. Visual inspection of the gel confirms that amplification of WT DNA results in multiple unspecific bands in absence of the reverse primers or both forward and reverse primers. Mut is the PCR-amplified WT DNA in presence of all primers. **b**-**c** RNA droplets transcribed from PCR-amplified WT DNA in absence of all primers (**b**) or of just the reverse primers (**c**) after 48 hours of transcription. RNA droplets are shown in red, with MGA labelled with malachite green dye, $\lambda_{ex} = 640\,\mathrm{nm}$. For **b**-**c** experiments were reproduced at least twice independently with similar results. Scale bars represent $100\,\mu\mathrm{m}$.

4.12 Agarose gel electrophoresis and RNA droplets of the WT2 DNA after mutagenesis

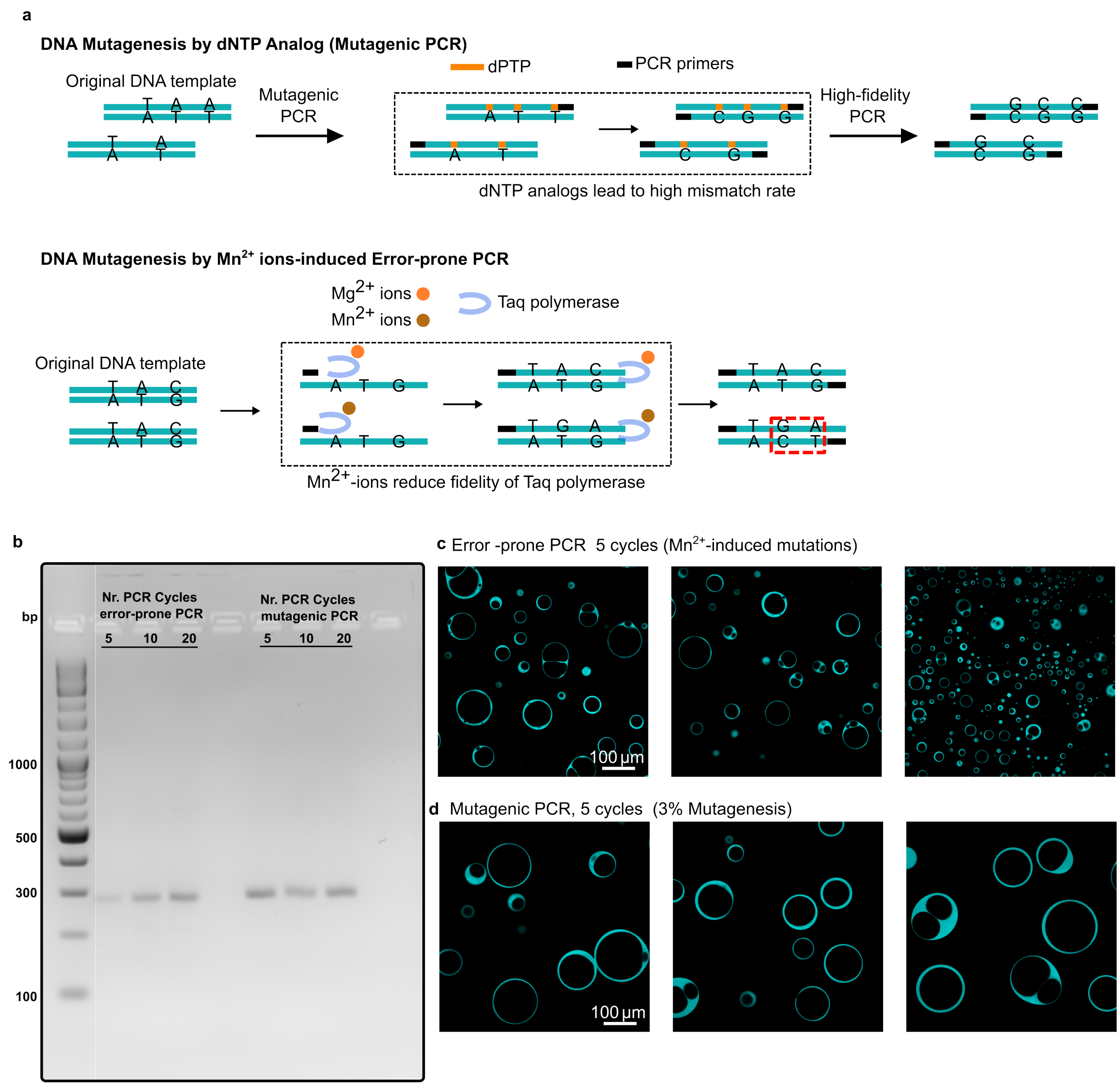

Supplementary Fig. 12: **3% AGE and RNA droplets of the WT2 DNA after being mutated through ion-induced error-prone PCR and dNTP-induced mutagenesis.** **a** Schematic of the mutation strategies used. **b** 3% AGE of mutated WT2 DNAs. For the ion-induced error-prone PCR, Nr. PCR cycles describes the number of PCR cycles performed in the presence of manganese using *Taq*-polymerase (**a**, bottom). For the mutagenic PCR, Nr. PCR cycles describes the number of cycles performed in the presence of dPTP.[14] Following the mutagenic PCR, a high-fidelity PCR is performed, to remove dPTP nucleotides, as described in Methods in the main text. The number of cycles controls the mutation rate. For mutagenic PCR, 5, 10, 20 cycles correspond to a mutation rate of $\sim 3\,\%$, $\sim 6\,\%$, $\sim 10\,\%$, respectively. Since it is substitution mutagenesis, the length of the mutated DNAs remain unchanged. The length of the DNAs present in the ladder (NEB, 1 kb Plus DNA Ladder, Cat No. N3200S) is reported on left side. First lane after the ladder was removed for visualisation, as indicated by the thin white line on the right side of the ladder, for the complete uncropped gel see the figure at the end of the SI. **c-d** Confocal snapshots were taken after 48 hours of transcription. RNA droplets are shown in blue, with the broccoli aptamer labelled with DFHBI-1T, $\lambda_{ex} = 488\,\mathrm{nm}$. Scale bars, $100\,\mu\mathrm{m}$. The corresponding data is shown in Fig. 3b,d of the main text. For **c** and **d** experiments were reproduced twice independently with similar results.

4.13 Percentage of vacuolated droplets for WT2 and its mutated variants

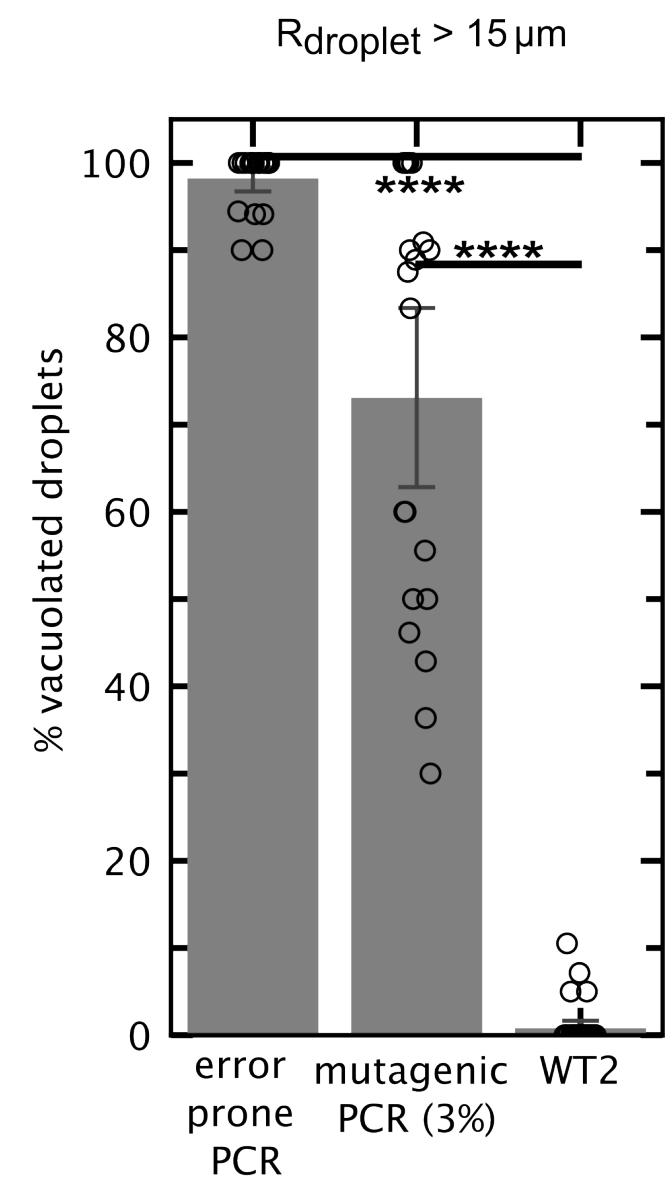

Supplementary Fig. 13: **Quantification of the percentage of vacuolated droplets for WT2 and its derived mutated variants.** Error-prone PCR refers to DNA amplified with *Taq*-polymerase in the presence of manganese after 5 cycles. Mutagenic PCR refers to DNA amplified with the dNTP analog, dPTP. Statistics: median test: significance with p-values < 0.001 are marked with ****. $P = 6.6 \times 10^{-10}$ (error prone PCR VS WT2) and $P = 1.1 \times 10^{-9}$ (mutagenic PCR VS WT2). Droplets are grouped per field of view ($n_{errorprone} = 21, n_{mutagenicPCR} = 20, n_{WT2} = 35$, from 2 independent replicates). The error bars denote the 95% confidence interval. Note that occasional small vacuoles were present in large WT2 RNA droplets. However, these were negligible as shown in the data presented in this graph.

4.14 Length Distribution of Sequenced Mut DNA and Predicted RNA Secondary Structure

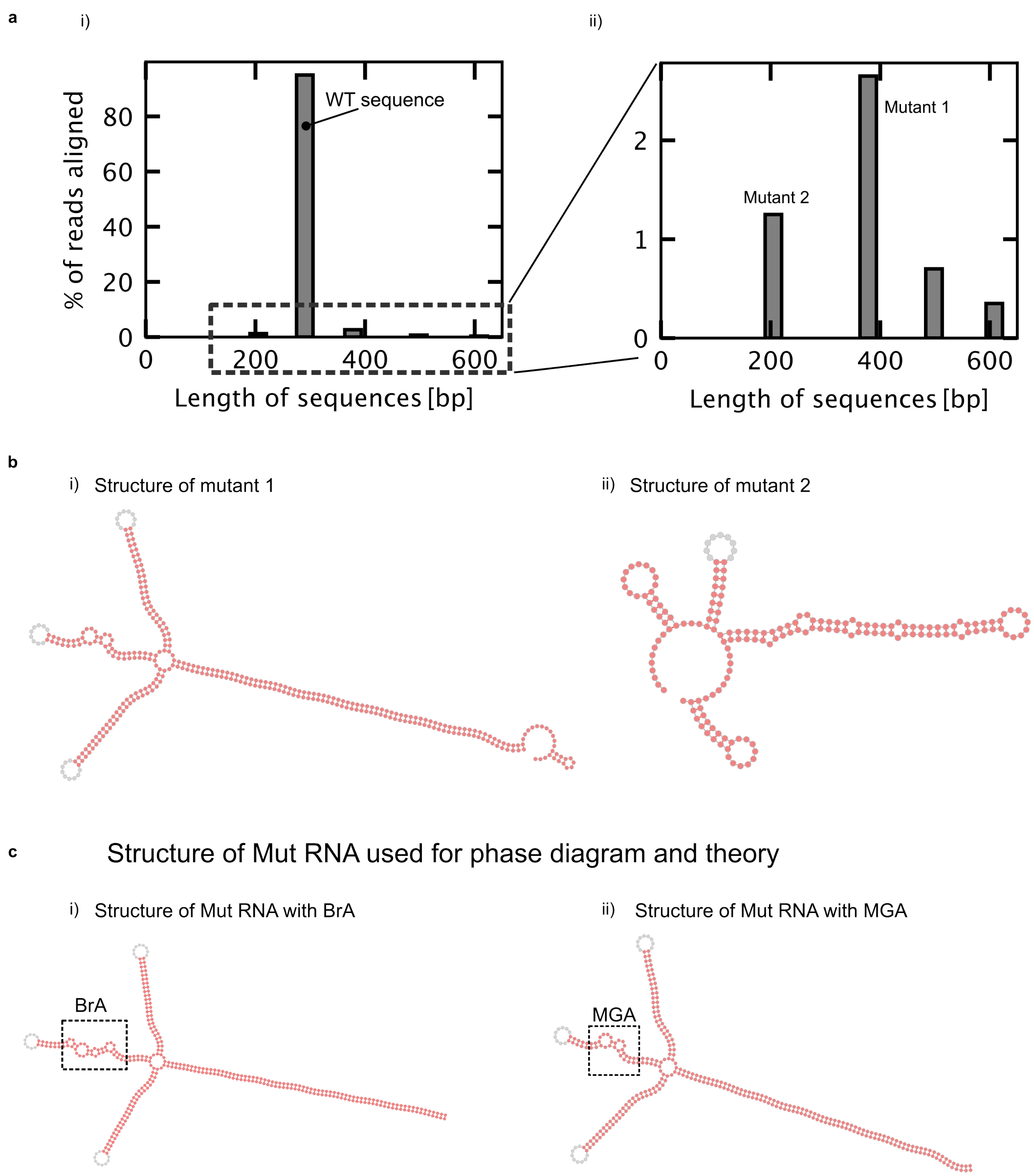

Supplementary Fig. 14: **Length distribution of the aligned reads.** **a** Percentage of aligned reads (**i**) and zoom into the less abundant sequences (**ii**), WT sequence is omitted for clarity. Exact sequences are given in Supplementary Table 3. **b** Predicted structures of the most (annotated mutant 1, **i**) and second (annotated mutant 2, **ii**) most abundant reads from the mutant sequences. **c** Predicted structure of the Mut RNA with BrA aptamer used to build the phase separation diagram shown in Fig. 4b. The same structure with MGA instead of BrA is shown on the right for comparison. Dotted regions delineate the aptamer regions.

4.15 De novo mutant designs induce vacuole formation

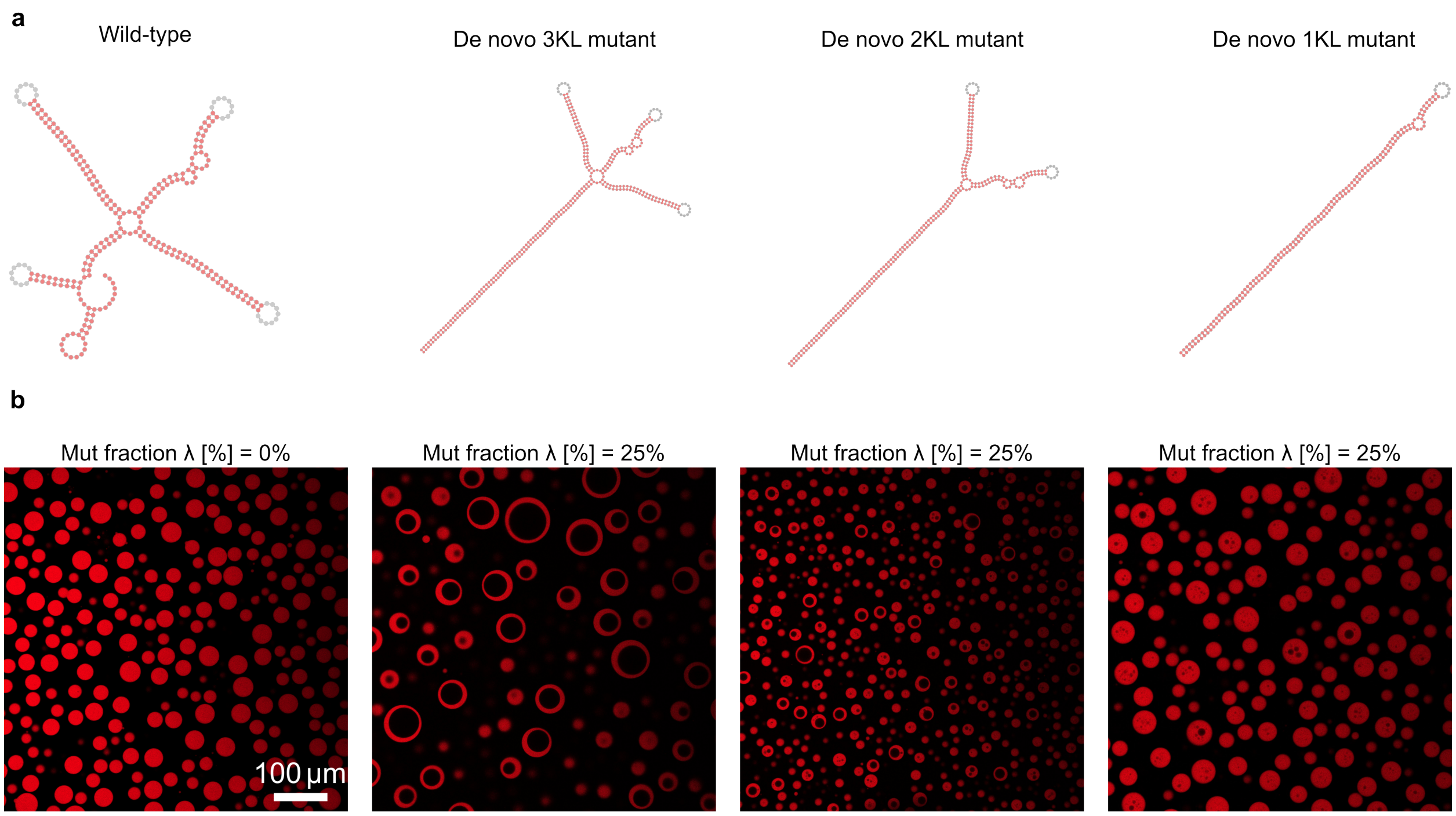

Supplementary Fig. 15: **De novo mutant designs induce vacuole formation.** **a** Predicted secondary structures of the wild-type (WT) RNA nanostar (left) and three de novo designed mutant nanostars (3KL-1KL Mut). The mutants were designed to preserve the characteristic secondary-structure motif identified for the most abundant mutants in the sequencing data (Fig. 4a and Supplementary Fig. 14). The secondary structure of the mutant used for the mechanistic model, shown in Fig. 4a of the main text, corresponds to the 3KL mutant. **b** Representative confocal images of RNA condensates formed upon co-transcription of the main WT band with each de novo mutant template (at mutant fraction λ of 25%), showing robust vacuole formation for all three *de novo* designs. All nanostars (red) contain the malachite green aptamer and droplets are labelled with malachite green dye ($\lambda_{ex} = 640\,\text{nm}$). Scale bar, $100\,\mu\text{m}$. Experiments were repeated twice independently with similar results.

4.16 Estimation of mass concentration of Mut and WT RNA in the dilute phase using calibration curve

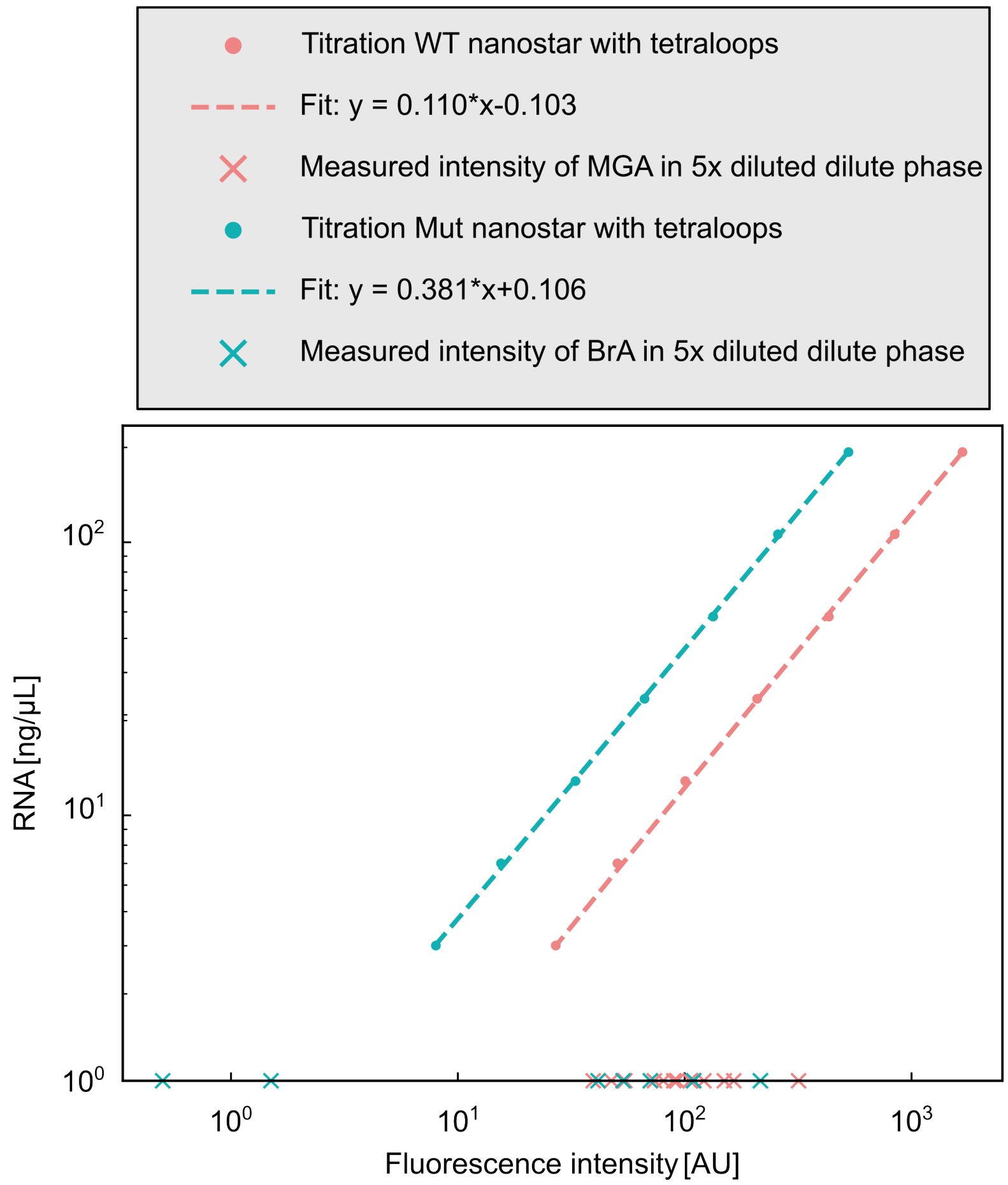

Supplementary Fig. 16: **Estimation of nanostar concentration in dilute phase.** Two-fold serial dilutions ($200\,\mathrm{ng\,\mu L^{-1}}$ to $3.125\,\mathrm{ng\,\mu L^{-1}}$) of tetraloop variants of the WT (Malachite Green aptamer, MGA, red colour) and Mutant (Broccoli aptamer, BrA, blue colour) RNAs. The RNAs are designed without kissing loops to prevent phase separation (see Supplementary Table 1). The fluorescence is measured at 37 °C in the assay buffer. RNA concentrations are plotted against their background-subtracted fluorescence. Circles denote experimental read-outs and dashed lines are linear fits (equations shown in figure legend) used for conversion of fluorescence to concentration. The WT series and its fit are shown in red. The Mut series and its fit are shown in blue. Cross marks are fluorescent signals from the corresponding $5\times$-diluted dilute-phase supernatant (corresponding concentrations reported in Supplementary Table 4). Excitation/emission settings: MGA, $620\,\mathrm{nm}$ to $660\,\mathrm{nm}$; BrA, $485\,\mathrm{nm}$ to $507\,\mathrm{nm}$. Each dilution point is measured in two technical replicates and their mean is plotted and used for fitting (see section 1.10 for details). The plot is in log-log scale for visualisation.

4.17 Spinodal regions in WT-Mut RNA phase diagram

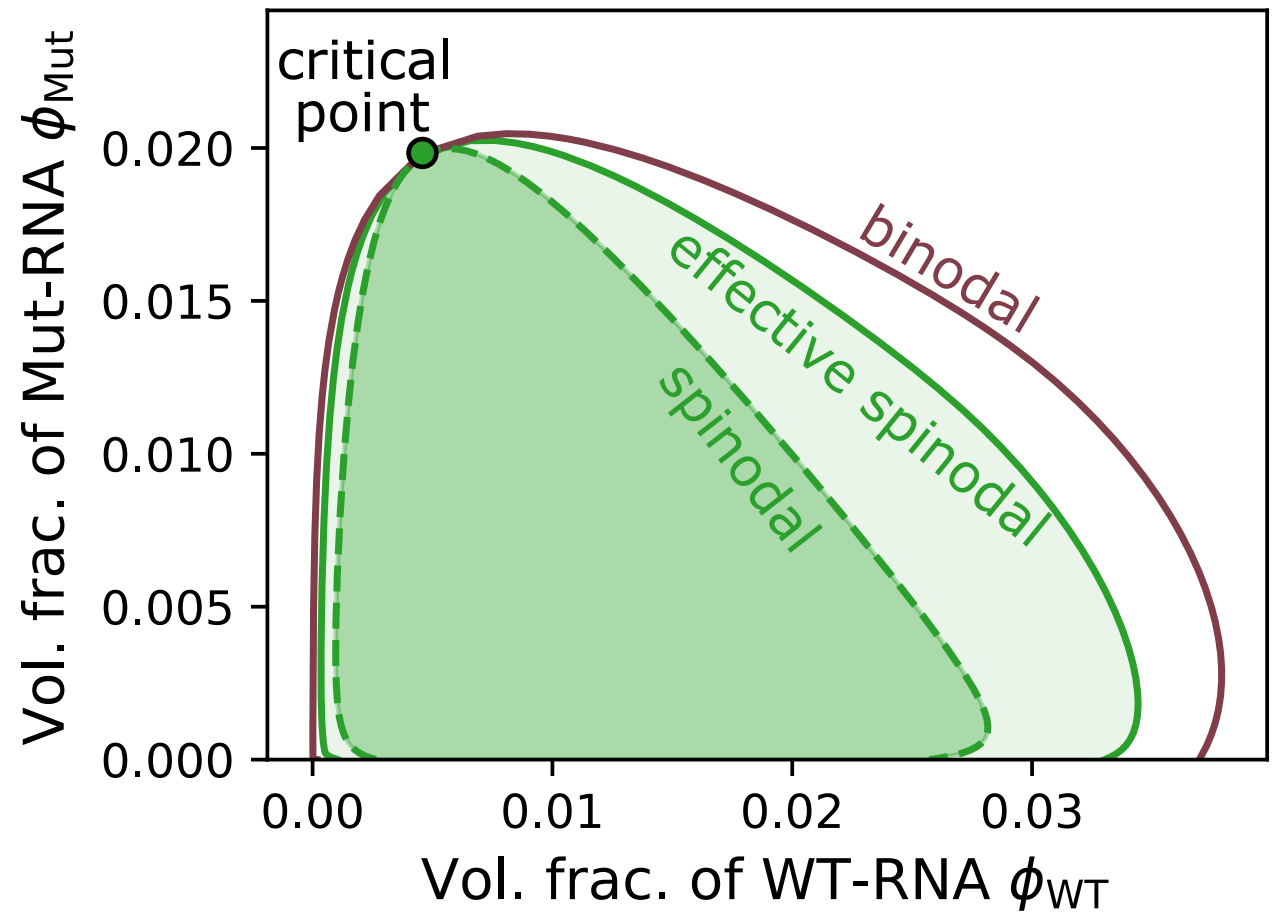

Supplementary Fig. 17: **Spinodal regions in phase diagram.** We introduced an effective solvent component to keep the model simple. These many different components can in general shift the spinodal line such that weak fluctuations can trigger the spinodal instability and thereby the formation of vacuoles. To account for this multi-component effect, we introduce an effective spinodal, which lies between the binodal and the actual spinodal.

4.18 Mechanism for vacuole formation

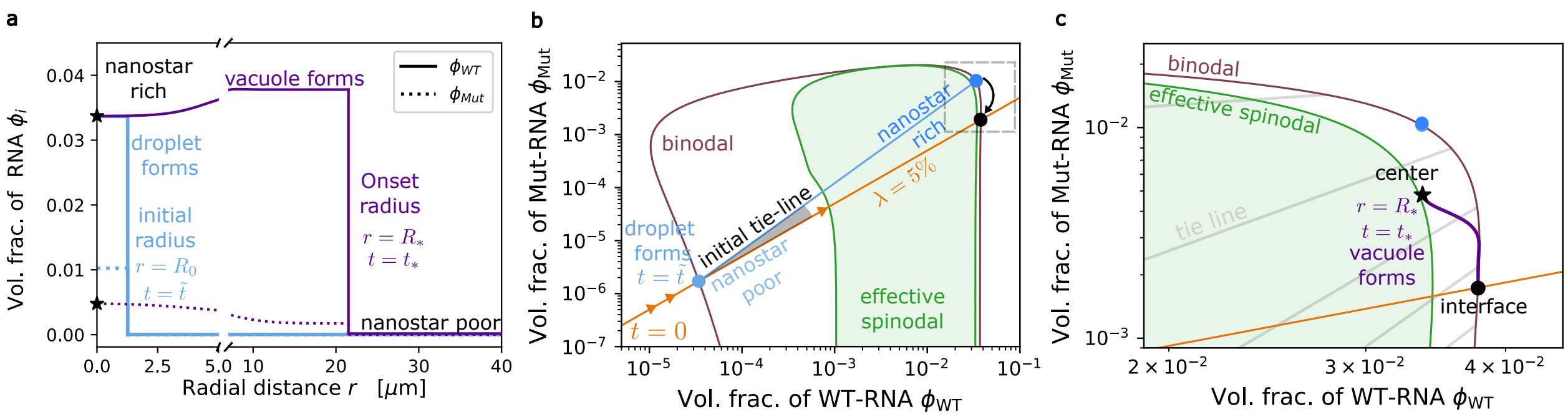

Supplementary Fig. 18: **Mechanism for vacuole formation. a** Interactions between the nanostars can lead to the formation of an RNA-rich droplet of radius R_0, which is in phase coexistence with the RNA-poor phase (shown in blue). This tiny droplet grows in time as transcribed RNA nanostars diffuse from the poor phase into the droplet from the outside phase. During growth, concentration gradients can emerge within the RNA-rich phase, reflected by the non-uniform profile shown in violet. **b-c** Transcription reaction produces RNA material while maintaining the fixed ratio of Mut to WT, denoted by λ (line in orange), see Eq. (7). At a later time $t = \tilde{t}$, an RNA-rich droplet coexists with an RNA-poor phase. The corresponding coexisting compositions are indicated by blue dots and are connected by the initial tie line. The deviation of the initial tie-line to the reaction constraint line λ, highlighted in grey marks a key thermodynamic parameter. The non-deviating initial tie-lines just lead to the growth of droplets without any formation of gradients in the RNA-rich phase. However, for tie-lines which deviate from the reaction constraint line upon transcribing further the dynamics of the RNA-rich phase need to walk down as indicated by the black arrow. The interface is always at phase-coexistence therefore, the interfacial composition evolves along the binodal. In contrast, away from the interface, in the RNA-rich phase composition starts to deviate from phase coexistence due to slow diffusion inside, giving rise to spatial gradients. This can be seen in the zoom-in panel **c**, where we plot the composition along the RNA-rich phase in violet. The system kinetics evolve over time such that it triggers a vacuole formation at a droplet with radius $r = R_*$ (see panel **a**), once the composition profile touches the spinodal (see star symbol)

4.19 Measurement of transcription rate and threshold nutrient concentration

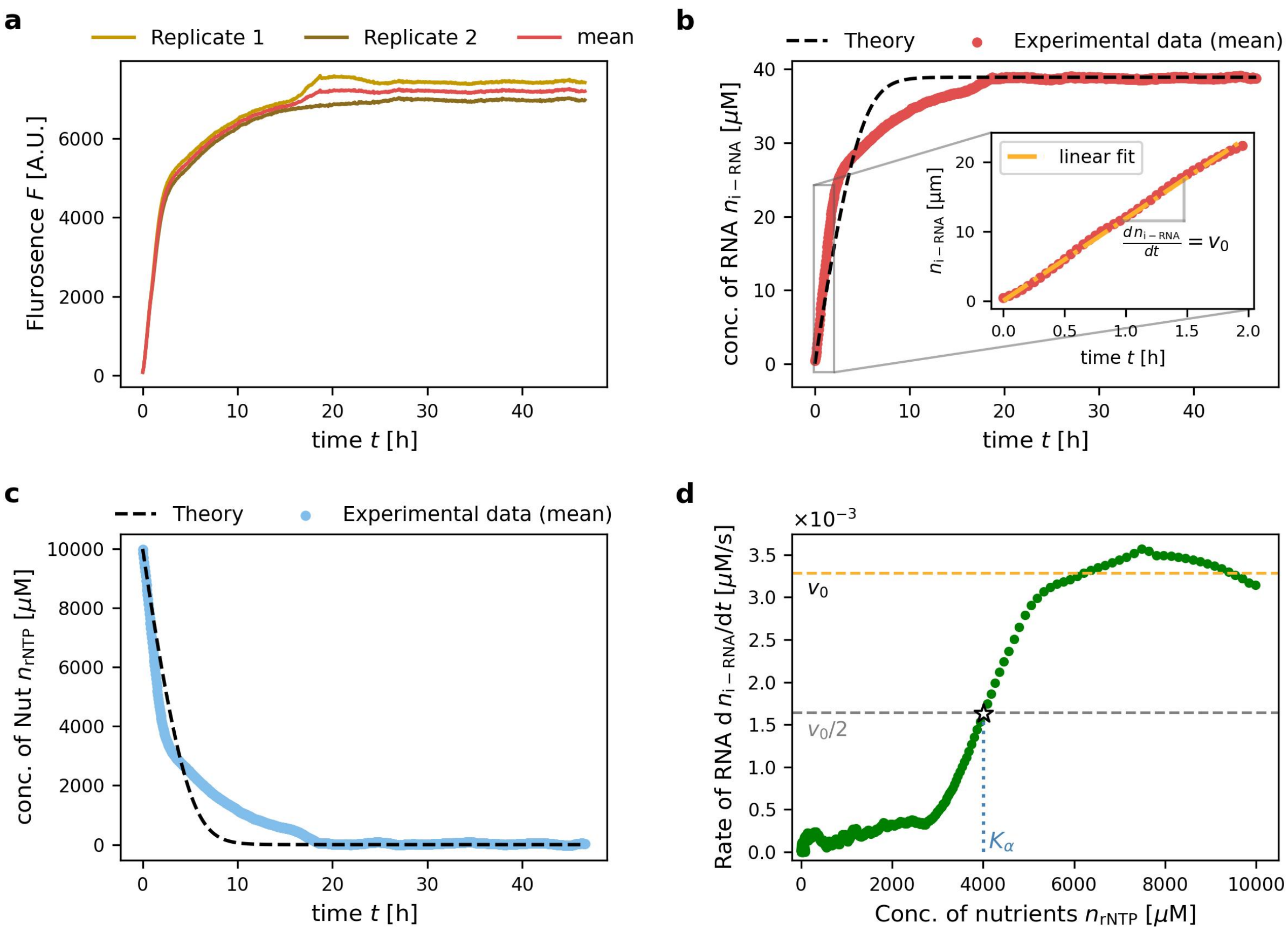

Supplementary Fig. 19: **Measurement of transcription rate and threshold nutrient concentration a** Raw fluorescence traces from two transcription reactions (n=2) and their mean, showing the accumulation of WT2 RNA over time. Fluorescence arises from DFHBI-1T binding to the Broccoli aptamer embedded within the WT2 nanostar, hence fluorescence increases with transcription. The plateau fluorescence is used to calibrate the signal into RNA concentration by assuming complete consumption of the initially supplied nucleotides. **b** Time evolution of WT2-RNA concentration $n_{\text{i-RNA}}$ (where i=WT2): the theoretical prediction (black dashed line) closely follows the experimental data (red) across the full reaction. The inset highlights the early-time linear regime, where nucleotides are abundant and the production rate is approximately maximal. **c** Time-dependent nucleotide concentration inferred from the stoichiometric consumption of nucleotides (given in Eq. (27)) during RNA production. **d** RNA production rate as a function of nucleotide concentration. The transcription rate approaches zero at low nutrient concentration and saturates at high nutrient concentration. We obtain threshold nutrient concentration K_α when production rate is $v_0/2$.

4.20 Estimated onset radius of vacuolation

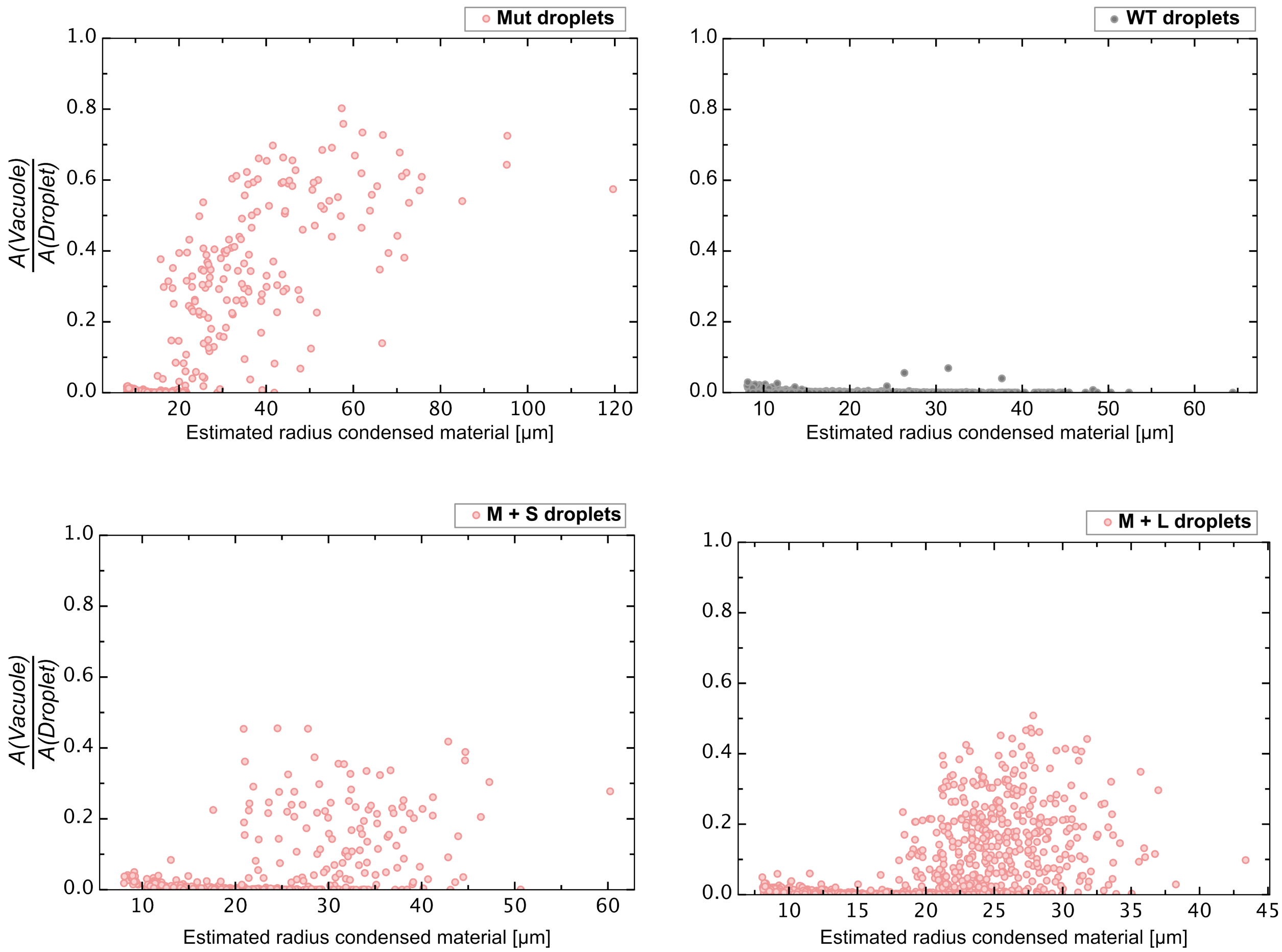

Supplementary Fig. 20: **Estimated radius of condensed RNA at the onset of vacuolation.** We estimate the minimal droplet size at which vacuolation occurs by measuring, in confocal images, the fluorescent area corresponding to the RNA-rich phase (i.e., excluding the dark vacuole; see SI Sect. 1.2 for details). Assuming that the RNA content is conserved during vacuole formation and that droplets are approximately spherical, the RNA-positive area after vacuolation equals the cross-sectional area of an equivalent full sphere without a vacuole. We therefore convert the detected RNA area A_{RNA} to an effective radius $R_{\mathrm{eq}} = \sqrt{A_{\mathrm{RNA}}/\pi}$ (and diameter $D_{\mathrm{eq}} = 2R_{\mathrm{eq}}$). In accordance with the theory, we find that only droplets above a certain size form vacuoles; for Mut droplets and droplets transcribed with M+L and M+S, vacuolation starts at an effective radius of roughly $15\,\mu\mathrm{m}$ to $20\,\mu\mathrm{m}$. This is close to the value predicted by our theoretical model for WT with $\lambda = 5\%$ Mut-RNA, shown in Fig. 4d of the main text. Datapoints represent individual droplets imaged from at least 2 independent repeats for each condition.

4.21 Experimental validation of the theoretical predictions for vacuole formation

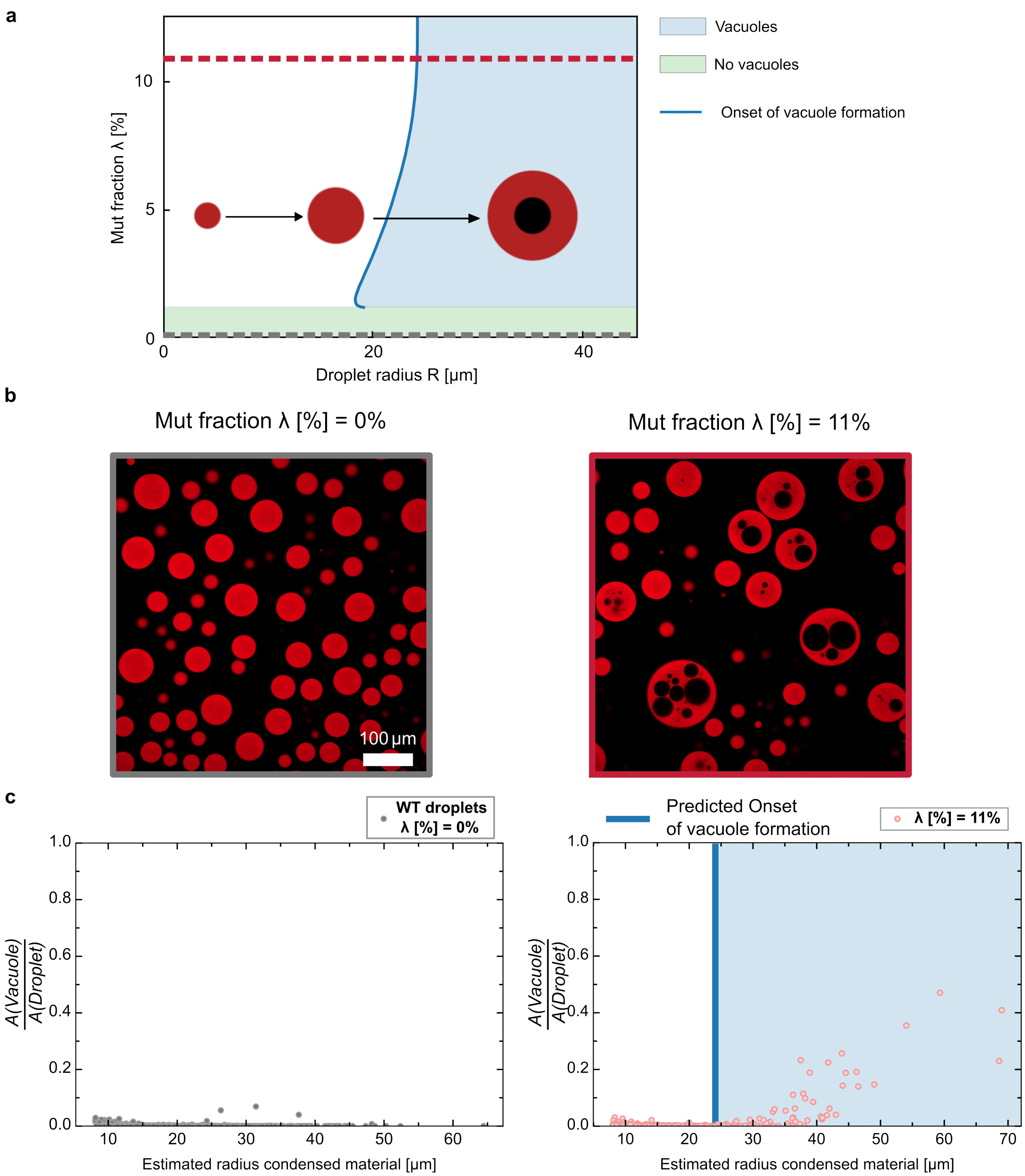

Supplementary Fig. 21: **Experimental validation of the theoretical predictions for vacuole formation. a** Schematic of the model prediction: within our experimental conditions, vacuoles are expected only above a characteristic droplet size and only when a finite fraction of mutant species is present (blue region indicates the predicted vacuole-forming regime; dashed lines mark the mutant fraction used below). **b** Representative confocal images of droplets formed by transcription of WT only ($\lambda = 0\%$, left) and of WT with the reconstructed mutant template at $\lambda = 11\%$ (right). Scale bars, $100\,\mu\mathrm{m}$. **c** Quantification of the droplet radius at which vacuoles first appear ("onset radius") for WT droplets ($\lambda = 0\%$, left) and for WT with $\lambda = 11\%$ droplets (right). For $\lambda = 11\%$ the measured onset radius ($\sim 26\,\mu\mathrm{m}$ to $30\,\mu\mathrm{m}$) is in good agreement with the theoretical prediction ($\sim 24.1\,\mu\mathrm{m}$). Datapoints represent individual droplets imaged from at least 2 independent repeats for each condition.

4.22 Annealing of WT and Mut nanostars to test whether Mut acts as a surfactant

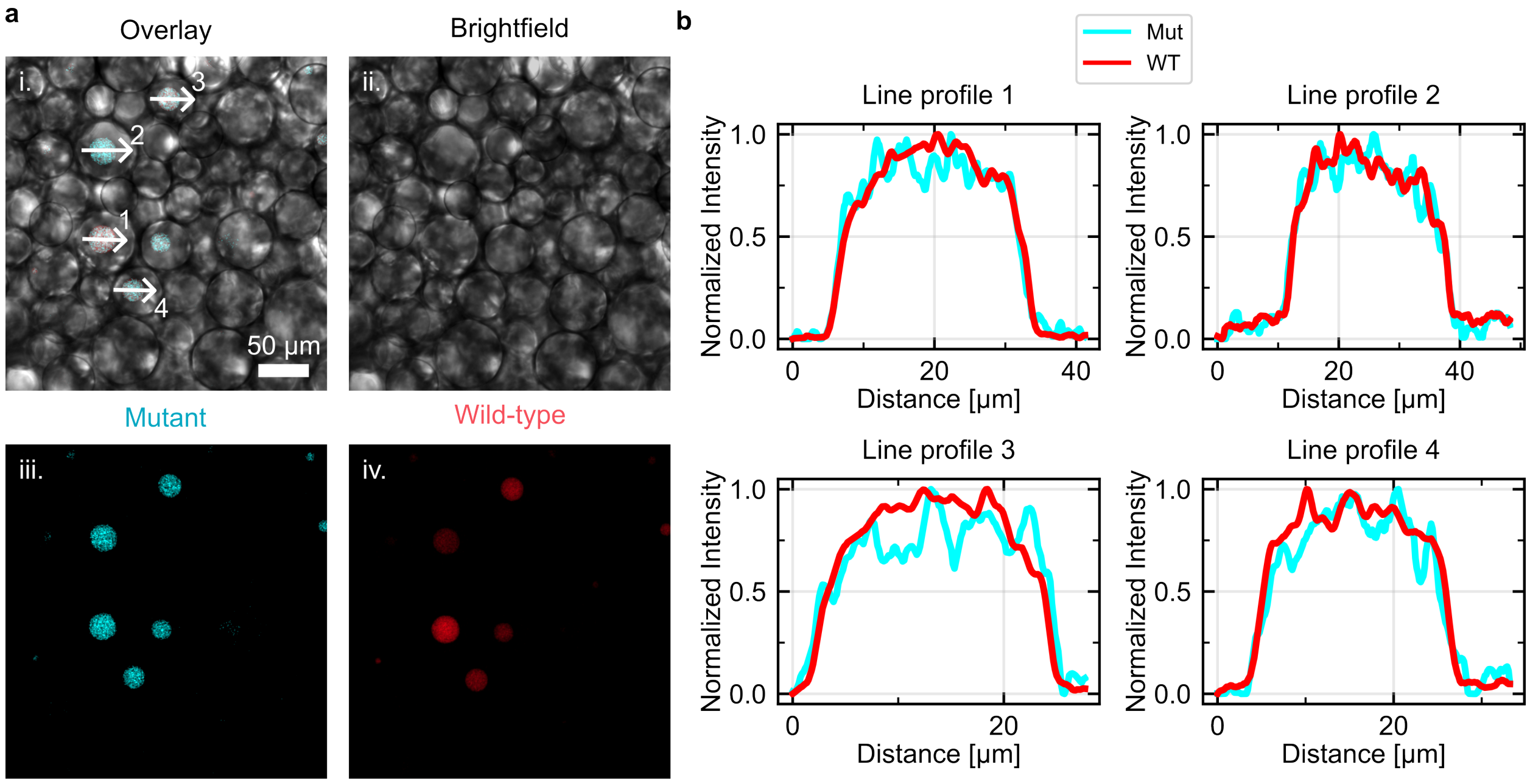

Supplementary Fig. 22: **Confocal imaging of WT and Mut nanostars to test whether Mut acts as a surfactant.** **a** Representative confocal image of condensates formed by annealing purified WT and Mut RNA nanostars inside water-in-oil droplets. WT nanostars (red) contain the malachite green aptamer and are labelled with malachite green dye (λ_{ex}=640 nm), Mut nanostars (cyan) contain the Broccoli aptamer and are labelled with DFHBI-1T (λ_{ex}=488 nm). Arrows indicate lines used for line profiles. **b** Normalised intensity profiles extracted from the droplets marked in **a**, comparing WT (red) and Mut (cyan) fluorescence across the droplet. Both signals are broadly homogeneous across the droplet cross-section and no systematic enrichment of Mut at the droplet interface is observed. Scale bar, 50 μm. All droplets shown are from a single field of view from a single experiment. Mut nanostars do not accumulate at the interfaces, which suggests that they do not act as surfactants.

4.23 Analysis of vacuole movement in relation to droplet movement

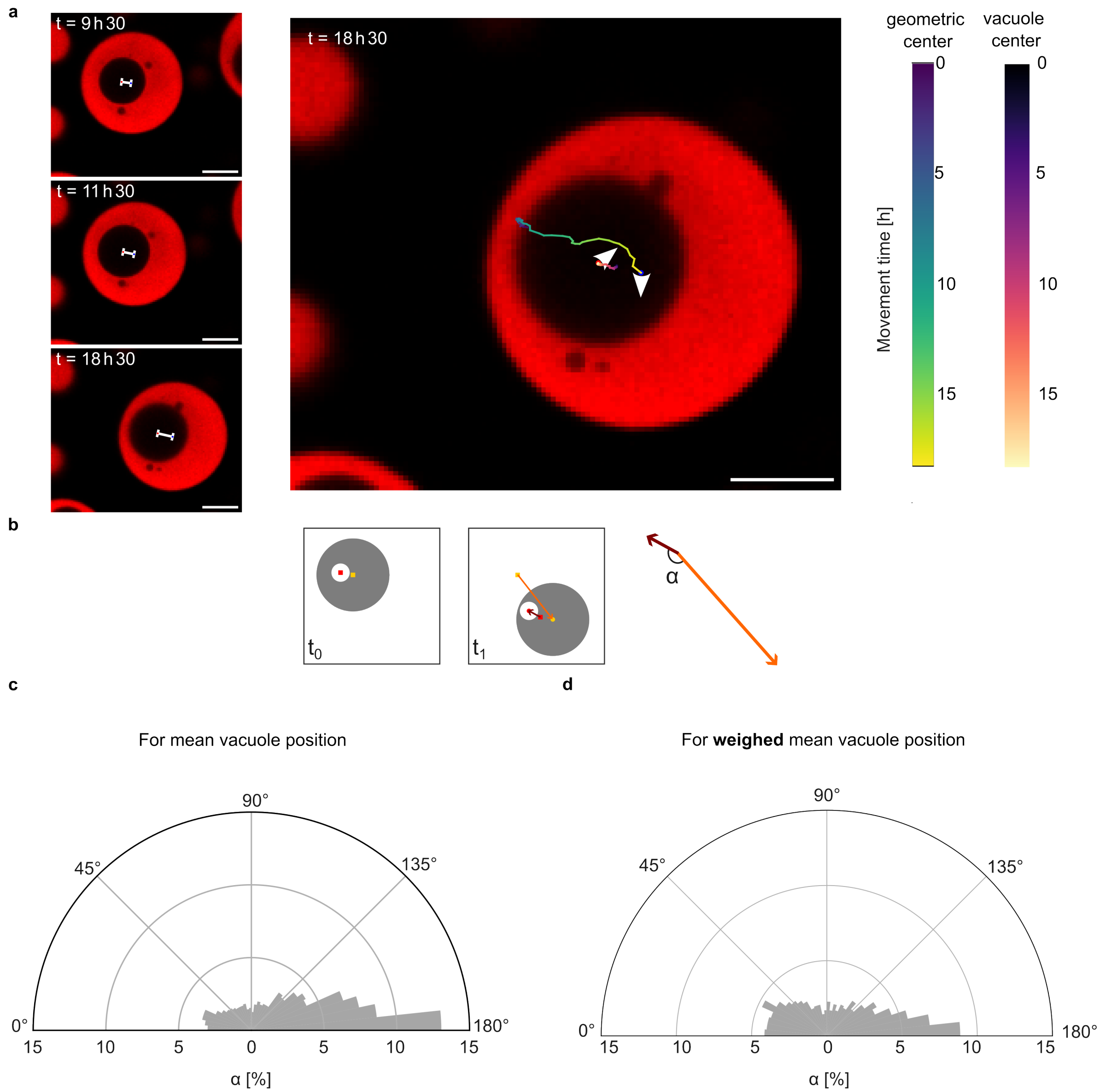

Supplementary Fig. 23: **Analysis of vacuole movement in relation to droplet movement.** **a** Confocal images of a moving vacuolated RNA droplet over time. Left panel: The increasing distance between the vacuole centre and the droplet's geometric centre indicates movement in opposite directions. Right panel: Movement of the droplet's geometric centre (green-blue) and the vacuole centre (orange-red) over time. Time is represented by colourbars on the right. Tracking time starts at $9.67\,\mathrm{h}$ after transcription initiation. White arrows indicate the direction of movement in the subsequent time frame. Scale bars, $20\,\mu\mathrm{m}$. Droplet contains MGA and is labelled with malachite green dye $\lambda_{ex}= 640\,\mathrm{nm}$ (shown in red). **b** Diagram showing the definition of angle α between the vacuole's direction of movement and that of the droplet's geometric centre. **c** Polar plot showing distribution of angle α calculated with mean of the vacuole centres. **d** Polar plot showing distribution of angle α calculated with weighted mean of the vacuole centres. The data is pooled from two independent replicates (see Supplementary Fig. 25 for individual replicate data). Although the highest distribution in both plots (**c-d**) remains in the same bin between $174°$ and $180°$, the angle distribution has become more dispersed in the distribution using weighted mean, with an increased frequency of angles in the other angle bins. Note that the weighted mean of the vacuole centres is calculated using the vacuole area as the weight (see Section 1.6). Since image acquisition occurs in a single confocal plane, not all vacuoles lie in the central plane, which can result in incorrect weighting and contribute to the spreading observed in the histogram (**d**). Therefore, the non-weighted averaged vacuole position is used for the data presented throughout the paper. $n_{angles} = 2458$

4.24 Burst-like movement of Mut RNA droplets

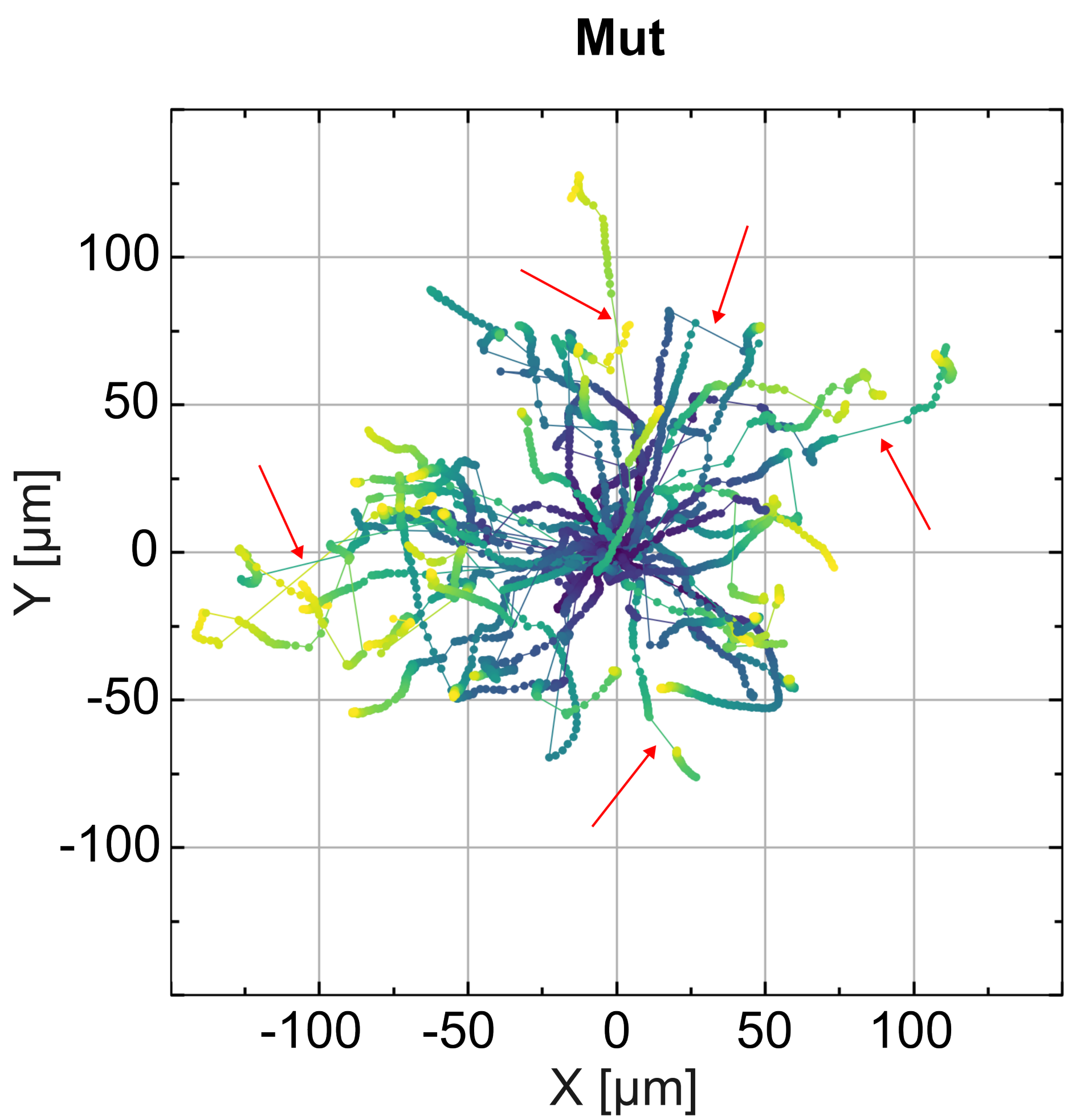

Supplementary Fig. 24: **Medusa plots of the movement of individual Mut RNA droplets.** We tracked droplet movement for over $50\,\mathrm{h}$ and only the 50 most mobile trajectories are shown. This plot is the same as main text Figure 5b right in the main text, but enlarged to highlight the burst-like movement of the RNA droplets, highlighted with red arrows. $n = 775$ Mut tracked trajectories.

4.25 Distribution of angle α across two different replicates

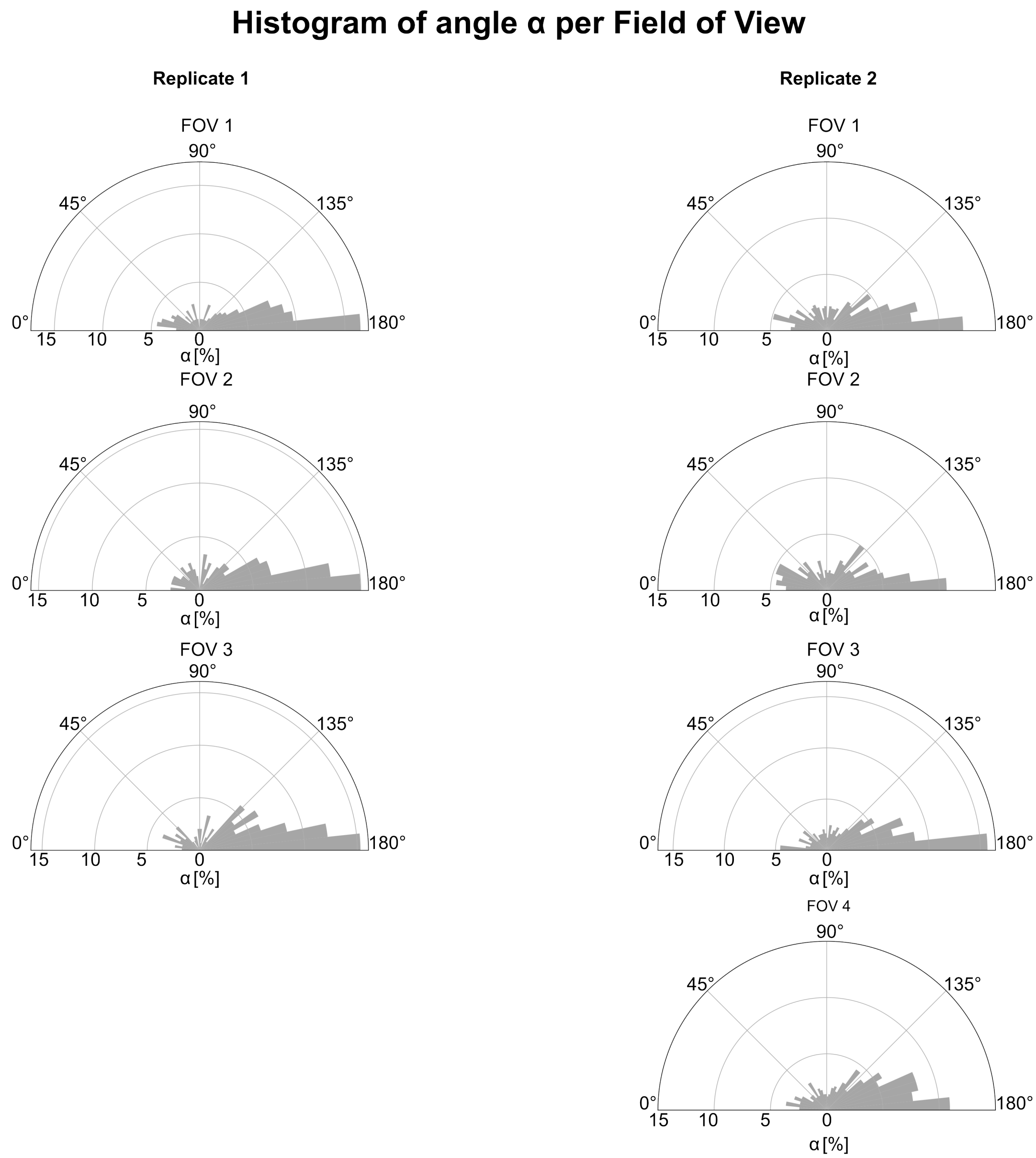

Supplementary Fig. 25: **Polar plots showing the distribution of angle α across two different replicates.** The angle α is calculated using the mean of the vacuole centres. We observe seven fields of view across two replicates, and the histograms of angle α from these seven fields of view are presented. The histogram in Supplementary Fig. 23c and Fig. 5d of the main text, represents the pooled data from these observations $n_{angles} = 2458$.

5 Supplementary Videos

5.1 Supplementary Video 1

Confocal time series showing the formation of WT RNA droplets (red, MGA is labelled with malachite green dye, $\lambda_{ex} = 640\,\mathrm{nm}$). Scale bar, $100\,\mathrm{\mu m}$. From the starting point up to 63 hours, every frame is separated by a 20 minute time interval. Timestamps are shown on the top left.

5.2 Supplementary Video 2

Confocal time series showing the formation of Mut RNA droplets (red, MGA is labelled with malachite green dye, $\lambda_{ex} = 640\,\mathrm{nm}$) and the appearance of vacuoles as well as droplet movement. Scale bar, $100\,\mathrm{\mu m}$. From the starting point up to 63 hours, every frame is separated by a 20 minute time interval. Timestamps are shown on the top left.

5.3 Supplementary Video 3

Bright-field time-lapse video of a vacuolated Mut RNA droplet showing the fusion of two small vacuoles. The fusion event is marked using a yellow arrow. Bright-field imaging provides a large depth of field, allowing vacuole dynamics to be followed more easily.

5.4 Supplementary Video 4

Additional bright-field time-lapse of a vacuolated Mut RNA droplet capturing a fusion event involving a large vacuole.

5.5 Supplementary Video 5

We present a movie of the dynamics obtained from the narrow-interface model described in Sec. 1.13, for a system where RNA transcription proceeds at a mutant fraction $\lambda = 5\%$. The movie consists of two panels. The left panel shows the spatial composition profile of the RNA-rich and RNA-poor phases and its temporal evolution. The right panel displays a cropped region of the RNA nanostar phase diagram from Supplementary Fig. 18b, where we track the time-dependent composition of the RNA-rich phase. The movie begins at $t = 0$ with a newly nucleated RNA droplet, corresponding to the state shown in Supplementary Fig. 18a. At this initial time, the RNA-rich phase is homogeneous and exhibits no internal gradients; its composition is marked by a green dot on the phase diagram (right panel). Over time, the droplet grows due to ongoing transcription. During this growth, composition gradients develop within the RNA-rich phase. The trajectory of the RNA-rich composition on the phase diagram reveals that the interfacial composition moves along the binodal, while the composition at the droplet centre gradually shifts away from the binodal due to slow diffusion. Eventually, the central composition enters the spinodal region, triggering the vacuole instability. The movie terminates at the onset radius $r = R_*$ and time $t = t_*$, marking the emergence of the instability. This final state is also shown in Supplementary Fig. 18a,c.

5.6 Supplementary Video 6

Confocal time series of a moving Mut RNA droplet. The positions of the droplet's geometric centre (blue) and the vacuole centre (red) are traced over time. The backtracks are colour-coded to indicate different time points. Tracking time starts at $9.67\,\mathrm{h}$ after transcription initiation and is set to zero. Droplet contains MGA and is labelled with malachite green dye $\lambda_{ex} = 640\,\mathrm{nm}$ (droplet shown in red).

6 References

[1] Rueden, C. T.; Schindelin, J.; Hiner, M. C.; DeZonia, B. E.; Walter, A. E.; Arena, E. T.; Eliceiri, K. W. *BMC bioinformatics* **2017**, *18*, 1–26.

[2] Ershov, D.; Phan, M.-S.; Pylvänäinen, J. W.; Rigaud, S. U.; Le Blanc, L.; Charles-Orszag, A.; Conway, J. R.; Laine, R. F.; Roy, N. H.; Bonazzi, D.; others *Nature methods* **2022**, *19*, 829–832.

[3] Sahlin, K.; Medvedev, P. *Journal of Computational Biology* **2020**, *27*, 472–484, PMID: 32181688.

[4] Sahlin, K.; Medvedev, P. *Nature communications* **2021**, *12*, 2.

[5] Li, H. *Bioinformatics* **2018**, *34*, 3094–3100.

[6] Fabrini, G.; Farag, N.; Nuccio, S. P.; Li, S.; Stewart, J. M.; Tang, A. A.; McCoy, R.; Owens, R. M.; Rothemund, P. W. K.; Franco, E.; Di Antonio, M.; Di Michele, L. *Nature Nanotechnology* **2024**, *19*, 1665–1673, Publisher: Nature Publishing Group.

[7] Laha, S.; Bauermann, J.; Jülicher, F.; Michaels, T. C.; Weber, C. A. *Physical Review Research* **2024**, *6*, 043092.

[8] Rumble, J.; others *CRC handbook of chemistry and physics*; CRC press Boca Raton, FL, 2017; Vol. 102.

[9] National Center for Biotechnology Information PubChem Compound Summary for CID 5957, Adenosine triphosphate. `https://pubchem.ncbi.nlm.nih.gov/compound/5957`, 2026; Accessed: 2026-06-11.

[10] Biffi, S.; Cerbino, R.; Bomboi, F.; Paraboschi, E. M.; Asselta, R.; Sciortino, F.; Bellini, T. *Proceedings of the National Academy of Sciences* **2013**, *110*, 15633–15637.

[11] Haugerud, I. S.; Jaiswal, P.; Weber, C. A. *The Journal of Physical Chemistry B* **2024**, *128*, 1724–1736.

[12] Stewart, J. M.; Li, S.; Tang, A. A.; Klocke, M. A.; Gobry, M. V.; Fabrini, G.; Di Michele, L.; Rothemund, P. W. K.; Franco, E. *Nature Communications* **2024**, *15*, 6244, Publisher: Nature Publishing Group.

[13] Xie, M.; Chen, W.; Vonk-de Roy, M.; Walther, A. *Nature Communications* **2025**, *16*, 8254, Publisher: Nature Publishing Group.

[14] Zaccolo, M.; Williams, D. M.; Brown, D. M.; Gherardi, E. *Journal of Molecular Biology* **1996**, *255*, 589–603.

7 Uncropped gel scans

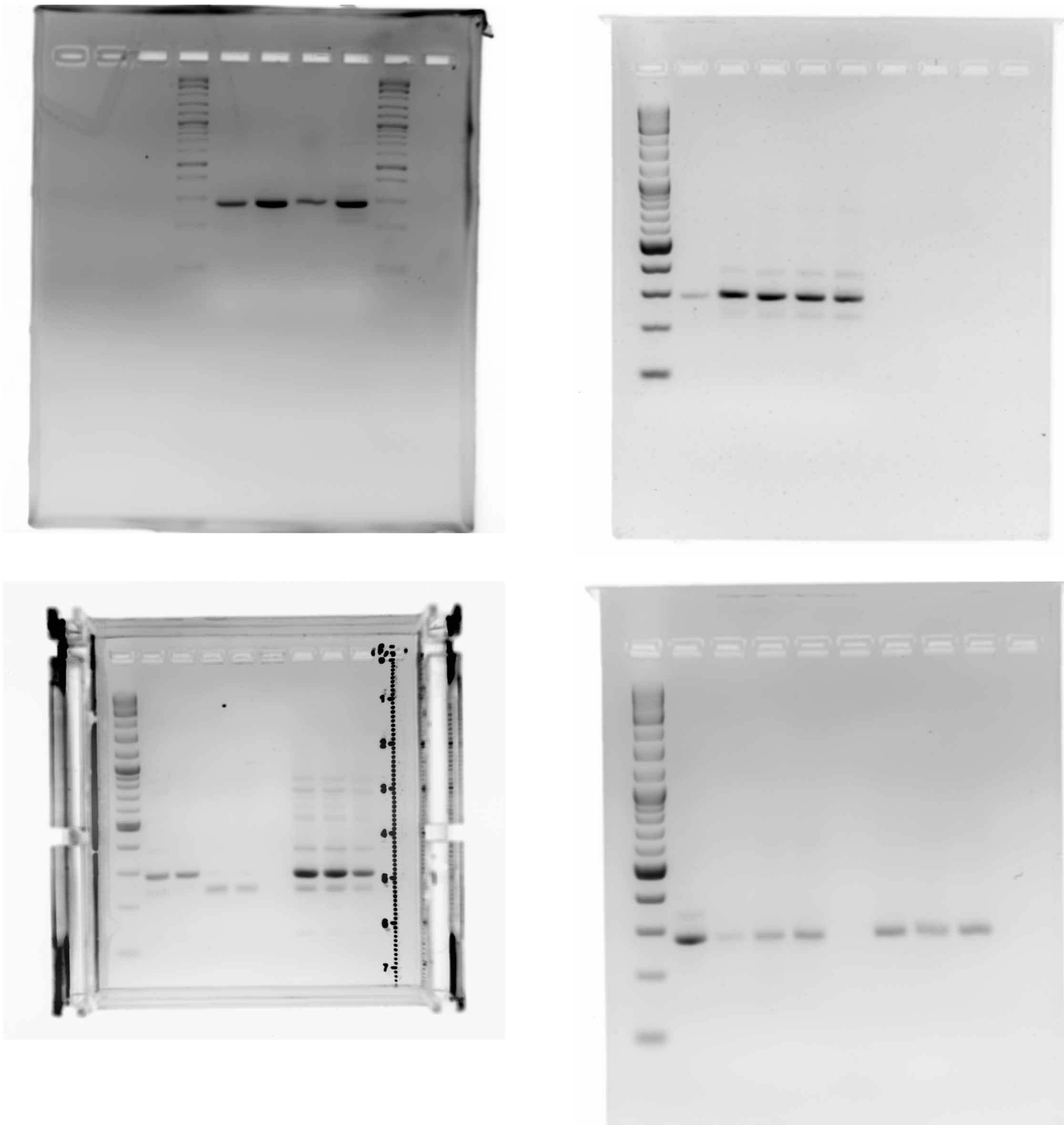

Supplementary Fig. 26: **Uncropped scans of all agarose gels shown in the Supplementary Information.** Each panel shows the complete scan of the gel from which the corresponding published panel was cropped. Top left: Full scan of the gel shown in Supplementary Fig. 1. Top right: Full scan of the gel shown in Supplementary Fig. 10a, which is also the gel shown in Fig. 2a of the main text. Bottom left: scan of the gel shown in Supplementary Fig. 11a. Bottom right: Full scan of the gel shown in Supplementary Fig. 12b; note that the lane immediately following the ladder, which was removed for visualisation in the published panel, is present here. No adjustments were applied to these images beyond inverting for clarity and linear brightness and contrast applied uniformly across the entire scan.
