# Supplementary material for: Genetic encoding and mutagenesis of RNA droplet phenotypes
Source: Nat Chem Eng. 2026 Oct 7;3(10):573–83. doi: 10.1038/s44286-026-00454-4 (PMC13680278; doi:10.1038/s44286-026-00454-4)
Supplement: Supplementary file 3 [file 44286_2026_454_MOESM3_ESM.pdf]

nature portfolio

Peer Review File

Genetic encoding and mutagenesis of RNA droplet phenotypes

Corresponding Author: Professor Kerstin Göpfrich

This file contains all reviewer reports in order by version, followed by all author rebuttals in order by version.

Version 1:

Reviewer comments:

Reviewer #1

(Comments for the Author)
The paper shows that RNA nanostar droplets produced co-transcriptionally from a DNA template not only encapsulate their own DNA, but can also evolve new architectures and behaviors when that DNA is mutated. Mutagenesis of the DNA template generates a minority population of “mutant” RNA nanostars that perturb the internal interactions of the condensate, driving stable vacuole formation, micron-scale droplet motility, and sub-compartmental cargo loading. This establishes RNA droplets as genotype–phenotype–function linked protocell-like systems suitable for experimental evolution and origins-of-life hypothesis.This study build on RNA nanostar which folds co-transcriptionally from a single RNA strand (adapted from Fabrini et al.)

Overall, this is a creative and well executed study that opens intriguing questions about the physical basis of genetically encoded architectures in RNA condensates.

Novelty. The system directly links sequence variation in the DNA template to measurable changes in RNA–RNA interaction strength and, in turn, to clear changes in droplet morphology. This creates a simple but informative genotype–phenotype connection at the level of material properties. The overall experimental design is solid and the main claims are convincingly supported. By mutating the DNA template, the authors show that vacuole formation can be genetically encoded. Non-specific PCR products (generated after many amplification cycles) appear to be responsible for the stable vacuoles observed in RNA droplets. Follow-up substitution mutagenesis then expands the phenotypic space. A mutation rate of ~3–10% produces a series of morphological transitions, ranging from vacuolated droplets to percolating networks and, eventually, RNA clusters. To address the mechanism of vacuole formation, the authors use a clever strategy based on WT/mutant binary mixtures, enabling the construction of a ternary phase diagram (dense phase, dilute phase, mutant fraction).

Minor points and Questions :
Given that non-specific PCR products are sufficient to produce vacuoles, could this information be used to design vacuole-forming sequences de novo?
The authors propose that vacuole formation originates from a local spinodal decomposition. It would be helpful if the authors could justify why spinodal demixing is the favored pathway here rather than classical nucleation. In large droplets, spinodal decomposition may typically typically produces patterns with a characteristic length scale at early times (competition between molecular interaction and interfacial cost). Is such an early-time length scale observable? Relatedly, does vacuole coarsening follow a $t^{1/3}$ scaling? Could a dilute “vapour-like” RNA phase inside a dense RNA phase also be considered? Do the relaxation dynamics of two interacting vacuoles provide clues about interfacial tension or osmotic pressure differences?

The mechanism depends on slow internal mixing relative to surface-driven material input. This condition is specific to the present RNA system and may not hold in protein condensates where exchange and turnover are faster.
The movies raise additional questions about vacuole dynamics:
– How does the presence of vacuoles influence droplet fusion?
– Why do multiple vacuoles often coexist instead of coalescing into a single interior cavity?
- Is their a Oswald rippening-like mechanism driving the dynamics of vacuoles ?
– Some vacuoles tend to disappear: are they simply out of focus or do they actually dissolve/shrink?

The proposed mechanism of droplet motility is that vacuole growth generates internal mass redistribution and displaces the center of mass, leading to droplet motion. This is plausible, but the current explanation is a bit short. Is their any analogy with self-propeled droplets in active systems ?

(Remarks on code availability)

Reviewer #2

(Comments for the Author)
The article by Vestraeten et al. presents a system of RNA-based synthetic condensate protocells that are under active control of a promoter, i.e. in which the RNA that constitutes the condensate is created in situ by transcription from a DNA template. While this has been done before, the novelty here lies mainly in the introduction of mutations in the DNA template that create mutant RNA species that alter the behavior of the droplets, most notably through creation of vacuoles. The authors characterize the mutant RNA by sequencing, then construct mimics of the mutant/wild type mixture that are able to reproduce the vacuole phenotype. They present a theory to explain the vacuole formation, and show that vacuole formation leads to novel droplet behaviors (enhanced diffusion, compartmentalizing other solutes).
Overall I found this to be a highly creative article. I agree that the work described has taken a significant step towards the creation of a DNA droplet system capable of some sort of evolution, though the present article falls well short of actually achieving that. I also appreciate that they brought a range of new techniques to bear on the field of nucleic acid condensates. The introduction of mutagenesis, and the use of sequencing to characterize the RNA, are very nice methodological advances. The specific results are interesting, albeit slightly confusing mechanistically (see below). I am tentatively inclined to be in favor of publication, though I have multiple queries of varying importance:
1) The discussion of ‘prerequisites for evolution’ in the introduction is interesting, but points (ii) and (iii) are hard to distinguish; i.e. they seem to say the same thing. Thus it is not obvious how the experiments have separately achieved (ii) vs. (iii). Can this be clarified? Also, I would think there is origin of life literature discussing definitions of minimal evolvable entities, but the definition (i,ii,iii) is not referenced; this should be rectified.
2) It is mentioned in the intro, and clear in the supplementary videos, that the vacuoles are extraordinarily stable, persisting in close proximity without merging (fusing) for up to 10 hours. Indeed it was hard for me to identify more than one or two vacuole fusion events (out of ~100s of vacuoles) in the very long supplementary videos. This is hard for me to understand without some specific stabilization mechanism—I am curious if it might be the case that the Mut RNA might act as a surfactant that stabilizes the vacuoles. There is mention of separate fluorescent labeling of the Mut and WT RNA; can these be imaged in different colors to see if there is accumulation of the Mut on the interface?
3) A general issue is that the system is much more complex than just a mixture of RNA species. Can the authors comment on the potential role of the RNA polymerase (which after all has DNA and RNA binding abilities), or the DNA template, in affecting the mixture and/or the droplet morphology? E.g. what happens if purified nanostars are mixed without polymerase or DNA present? I could imagine an argument that the concentrations of these other species so low as to not affect mixing or morphology, but if this is the stance of the authors, it should be made explicit.
4) I found it very interesting that the DNA template was engulfed within the RNA droplets, but I found the article to not use that fact in certain interpretations. First, is there any correlation between the location of vacuole appearance and the location of DNA clusters? Second, the model explaining the vacuole appearance assumes that most of the new RNA is formed outside of the droplet, which seems to contradict the fact that most of the template is inside in the droplet. Actually, I am not sure if ‘most’ of the template is inside; the images and discussion just indicate a lot is inside, but I take the point in the supplement that most of the volume is outside. So I suppose there are two related questions: i) can the authors make this all a bit more quantiatitve, e.g. by estimating the partition coefficient of DNA template in/out of the droplets (perhaps from fluorescent confocal measurements) and/or commenting on the volume ratio in/out of condensed phase; ii) does such an estimate support the assumption that most RNA is formed outside the droplet?
5) Droplet areas are used throughout the paper to quantify the amount of condensed phase. Area can be strongly affected by wetting, so it would be useful to show a confocal stack showing whether the droplets are wetting their container (the glass surface).
6) The explanation of the spinodal decomposition relies on a materials property, i.e. the slow diffusion of nanostars in the dense phase, which they support by reference to other works. This seems fine to me, except I think it would be useful to add at least some direct estimate of materials properties to the paper. Given the videos, it seems straightforward to estimate the inverse capillary velocity from the timescale of droplet fusion events. It would be helpful to report this value, so that the behavior of this particular system can more directly be compared to other systems.
7) While I applaud the creativity in creating two different types of mutants (through duplication/deletion in Fig 1, then the Fig. 3 substitutional approach), I will admit I found the switch to not be clearly motivated. If possible, it would help to add a few words clarifying the benefits of the two approaches, or the need to switch to a second method.
8) After much study, I believe (possibly naively) that I came to understand the model that is fleshed out in the supplement, and commented only briefly in the main text. Ultimately I feel that the authors should consider revising the main text paragraph to get the point more clearly across. However, let me try to re-state what they are proposing: just before phase separation occurs, the Mut/WT ratio in the homogeneous system is \lambda. Then phase separation occurs; the total ratio is still \lambda, but the actual ratio in each phase is no longer \lambda (being instead whatever ratios are found at the ends of the tie lines). But the transcription continues, driving (according to the assumption I asked about above) the dilute phase towards \lambda, which is not in equilibrium with the conditions inside the dense phase. This gives rise to the gradient and the spinodal decomposition. Is this the explanation? If so, then I wonder if adding some clear comments in the main text about the key issue—that initial equilibration at a bulk ratio of \lambda is later driven out of equilibrium by active maintenance of the dilute solution at ration \lambda—is better explained.

9) Some clarification of timing would be useful. The methods indicate transcription was allowed to proceed for 48 hours before imaging; is this really how data like Fig. 1d and 1e were acquired? Specifically, those panels show time points of 1h, 4h, etc; do those refer to 1h, 4h, etc., after the start of transcription, or 49h, 53h,…, after starting? Generally the very long times of the experiments are notable; is the RNA polymerase still active and transcribing throughout all this time (i.e. over multiple days)? Does the system run out of nucleotides?

(Remarks on code availability)

Reviewer #3

(Comments for the Author)
The authors showed that cotranscriptional condensation of RNA nanostars, which self-assemble via kissing-loop interactions, encapsulate their own DNA templates. By introducing mutations into the DNA templates, they showed that mutant nanostars drive the formation of long-lived vacuoles within the droplets. Theoretical modeling suggested a plausible mechanism for vacuole formation. Finally, the authors showed that these nonequilibrium systems give rise to spontaneous droplet motion and can be used for cargo sequestration. Taken together, the authors suggest that these systems exhibit several essential features of minimal synthetic cells.

In my opinion, the interesting results that are presented in this manuscript could be presented more clearly, both through specific clarifications and also potentially a reorganization of the document. I highlight primary areas of concern below:

1. The authors emphasize at the outset that RNA droplets encapsulate their DNA template upon formation, but it is not clear from the main text whether this must always be the case or whether this phenomenon is specific to the system studied here. Furthermore, the mechanistic reason for this encapsulation is not addressed.

2. The explanation of mutagenesis via PCR (second paragraph of the Results) is very brief given the importance of this to the subsequent results. A schematic figure in the main text that shows how self-complementary RNA act as primers would be helpful. Also, does PCR imply that the temperature is cycled, and if so, what happens to the droplets during this process?

3. I found the explanation of the experimental results on various mutagenesis schemes to be confusing because the specific hypotheses that were being tested were not clear. Why did the authors choose to perform mutagenesis in these particular ways? Is the key conclusion from all of this simply that mutagenesis, essentially no matter the details, leads to vacuole formation? Perhaps I missed it, but I did not see how these experiments were guided by rational hypotheses or how they led to a mechanistic understanding.

4. Although a mechanistic understanding is provided by the theoretical model, most of the details required to understand the explanation are contained in the supplementary information. In my view, it must be possible for the reader to understand the central conclusions of the theoretical model and how they relate to the experimental results in the main text. Several important aspects of the theoretical approach are omitted from the main text, including: Why is DNA not explicitly modeled in the free energy density? How do the sizes of the mutant and WT RNA enter into the theory, given that the main text only mentions the Chi parameters? If the spinodal is essential to the mechanism, why is it not shown on the phase diagram in the main text (the supplementary figures are much clearer)? In which direction do the concentration gradients go, and why does that lead to an instability (again, this is only clear in the supplement)?

5. How exactly is the phase diagram fit to the theory, and to what extent do the details matter? I ask because a key feature of the theoretical phase diagram is the negative slope of the lower arm of the binodal in the WT-Mut plane; however, there is no evidence supporting this in the limited number of experimental data points. Is this feature of the fitted phase diagram important for the model? If not, is a quantitative fit of the phase diagram necessary to provide a mechanistic explanation?

6. I was surprised to find that the main text does not explicitly state whether the vacuole formation is a kinetic or thermodynamic effect. This distinction is important in general, but even more so in this study because the authors claim that the vacuoles are "stable" at several places in the manuscript. Moreover, it is not clear whether the theoretical model addresses the origin of this stability.

7. I expected to see more connections between the theory and experiments. As currently presented, the theory is mostly developed on the basis of one reverse-engineered experimental system. However, the predictions of this theory do not appear to be tested experimentally except for the comment that gradients were observed transiently in a prior figure panel. Can the theory explain all the experimental results described previously, including the various behaviors of the M, M+L, and M+S systems in Figure 2 and the various morphologies observed in Figure 3? In my view, making connections to all the experimental results is essential to establish the mechanistic interpretation convincingly. Addressing this shortcoming would also help to address comment (3) above.

(Remarks on code availability)

Version 2:

Reviewer comments:

Reviewer #1

(Comments for the Author)
The authors have adequately addressed all the questions and comments raised during the review process.
Based on these revisions, I am satisfied with the responses and support the manuscript for publication.

Sincerely,

(Remarks on code availability)

Reviewer #2

(Comments for the Author)
I appreciate the long and detailed rebuttal provided by the authors. Many aspects of the article have improved as a result; particularly the logic of certain experiments and the clarity of the text.

Ultimately a fundamental experimental concern remains: In my initial review, I had asked (reviewer 2, comment 9) about the lifetime of the RNAP reaction, i.e. whether RNAP is still active and producing RNA throughout the 2-day time course of the typical experiment presented here. In response, the authors calculate the time to rNTP depletion. Here is their (supplementary) text describing that calculation:
The experimental system is prepared with an rNTP concentration of 10 mM and a DNA concentration of 45 nM. The transcription rate for the considered RNA composed of 200 nucleotides is about 1/s. We can thus estimate that it takes about 60 h to deplete most of the rNTP nutrients. We conclude that when vacuoles form (around 6h, see Fig. 1 main text and Supplementary Video S2), the T7 polymerase is active at rNTP excess.

I believe the authors miscalculated the depletion timescale. In fact, I can recreate their 60 hour number if I assume the RNAP transcription rate is 1 _nucleotide_ per second. However, the wording of their second sentence is more consistent with one _transcript_ being produced per second, implying a transcription rate of 200 nt/s. In fact, 200 nt/s is consistent with published estimates of T7 RNAP speed at 37C. Thus I believe the actual time to depletion is around 200 times faster, i.e. around 18 minutes rather than 60 hours (=3600 minutes).

These theoretical estimates correspond to the fastest possible depletion (e.g. making implicit assumptions that RNAP is saturating). Other effects could also serve to limit the reaction: e.g., multi-hour enzymatic reactions frequently become limited by the fragility of the enzyme itself. So it might be better to compare to published transcription experiments using T7 RNAP. From a quick literature search, I find several examples that report RNA production ceased after less than, or of order, 100 minutes in what appear to me to be roughly similar conditions, see e.g. DOI: 10.1038/s41598-024-59978-5, 10.1039/d5sm00868a, or the NEB technical note titled “Scaling of high-yield in vitro transcription reactions for linear increase in RNA production”. The longer experimental timescale vs. theory (100 min > 18 min) could be because the published experiments typically start with more than 10 mM rNTP (and so lasts longer), or because of reduced enzyme activity as the reaction proceeds, or some other mechanism.

I conclude that, after ~2 hours (and possibly even earlier), the rate of transcript generation is very likely to be nearly zero. This is before even the 6 hour timepoint of vacuole appearance, and so casts serious doubts on the interpretations provided by the authors. I welcome any correction to the estimates I provide and apologize if I made an error; but, failing that, the only way I see to resolve this is through direct measurement of RNA transcript production over time.

Finally, I will mention that, in trying to carry out these calculations, I ran into various difficulties due to a lack of clarity in the text. For example, the T7 RNAP concentration is not clearly stated. Further, despite my comments in the initial review, and despite what the authors say in the rebuttal, the Fig 1 caption has not been revised to clarify that the time points in Fig 1 d/e are times since the start of transcription; nor has the blanket statement in the methods that “all images are taken after 48 h of transcription” been revised to account for the Figure 1 data.

A final minor note: I appreciate the addition of materials properties, but the wording describing it is poor: the capillary velocity is presented on the bottom of page 3 in a sentence reading “Consistent with the long-lived nature of the vacuoles, fusion events proceed on long timescales (capillary velocity $\sim 10^{-2}\ \mu m\ s^{-1}$)…”. The way this is written makes it sound like the vacuoles are fusing (whereas they are analyzing droplet fusion events). Further, this sentence doesn’t spell out the actual logic the authors are proposing (which is that the material is highly viscous, which slows the dynamics of vacuole fusion).

(Remarks on code availability)

Reviewer #3

(Comments for the Author)
I find the revised manuscript to be substantially improved. The authors have clarified key points and worked to demonstrate the extent of agreement between the theoretical model and experimental results. In my view, the illustration and description of the vacuole-formation mechanism in the main text could be further improved, as this aspect of the manuscript was also unclear to other reviews and most of the adjustments have been made in the supplement; however, the current version is acceptable.

(Remarks on code availability)

Version 3:

Reviewer comments:

Reviewer #2

(Comments for the Author)
I appreciate the careful work that the authors have carried out to illuminate the transcription dynamics. I have no further comments, and feel that the is acceptable as it stands.

(Remarks on code availability)

Open Access This Peer Review File is licensed under a Creative Commons Attribution 4.0 International License, which permits use, sharing, adaptation, distribution and reproduction in any medium or format, as long as you give appropriate credit to the original author(s) and the source, provide a link to the Creative Commons license, and indicate if changes were made.
In cases where reviewers are anonymous, credit should be given to 'Anonymous Referee' and the source.
The images or other third party material in this Peer Review File are included in the article's Creative Commons license, unless indicated otherwise in a credit line to the material. If material is not included in the article's Creative Commons license and your intended use is not permitted by statutory regulation or exceeds the permitted use, you will need to obtain permission directly from the copyright holder.
To view a copy of this license, visit https://creativecommons.org/licenses/by/4.0/

Point-to-point response

Reviewer #1

"The paper shows that RNA nanostar droplets produced co-transcriptionally from a DNA template not only encapsulate their own DNA, but can also evolve new architectures and behaviors when that DNA is mutated. Mutagenesis of the DNA template generates a minority population of mutant RNA nanostars that perturb the internal interactions of the condensate, driving stable vacuole formation, micron-scale droplet motility, and sub-compartmental cargo loading. This establishes RNA droplets as genotype-phenotype-function linked protocell-like systems suitable for experimental evolution and origins-of-life hypothesis. This study build on RNA nanostar which folds co-transcriptionally from a single RNA strand (adapted from Fabrini et al.) Overall, this is a creative and well executed study that opens intriguing questions about the physical basis of genetically encoded architectures in RNA condensates.
Novelty. The system directly links sequence variation in the DNA template to measurable changes in RNA-RNA interaction strength and, in turn, to clear changes in droplet morphology. This creates a simple but informative genotype-phenotype connection at the level of material properties. The overall experimental design is solid and the main claims are convincingly supported. By mutating the DNA template, the authors show that vacuole formation can be genetically encoded. Non-specific PCR products (generated after many amplification cycles) appear to be responsible for the stable vacuoles observed in RNA droplets. Follow-up substitution mutagenesis then expands the phenotypic space. A mutation rate of $\sim 3-10\%$ produces a series of morphological transitions, ranging from vacuolated droplets to percolating networks and, eventually, RNA clusters. To address the mechanism of vacuole formation, the authors use a clever strategy based on WT/mutant binary mixtures, enabling the construction of a ternary phase diagram (dense phase, dilute phase, mutant fraction).

We thank the reviewer for their positive remarks on our work ("creative and well executed study"). We appreciate the thorough review and acknowledge the time that the reviewer put into reading our work and making good suggestions to push it further.

Comment 1: *"Given that non-specific PCR products are sufficient to produce vacuoles, could this information be used to design vacuole-forming sequences de novo?"*

The de novo design of vacuole-forming sequences is an interesting suggestion. Our results suggest that vacuole formation does not require one specific mutant sequence – instead it requires mechanistic properties.

To further test whether this structural information can be used for de novo sequence design, we generated additional mutant templates with similar secondary structures. Importantly, the KL mutant templates tested here are designed to reproduce the characteristic secondary-structure motif identified for the most abundant mutants in the sequencing data (Supplementary Fig. 14).

Specifically, we designed three variants with closely related secondary structures but differing numbers of kissing-loop (KL) interactions (de novo 1KL, 2KL, and 3KL mutants; Response Fig. 1a).

When transcribed together with the main WT template, all three de novo KL mutants yield vacuolated condensates (Response Fig. 1b). We have added these additional designs to the SI (Supplementary Fig. 15) and the corresponding discussion to the result section of the main text in the revised manuscript.

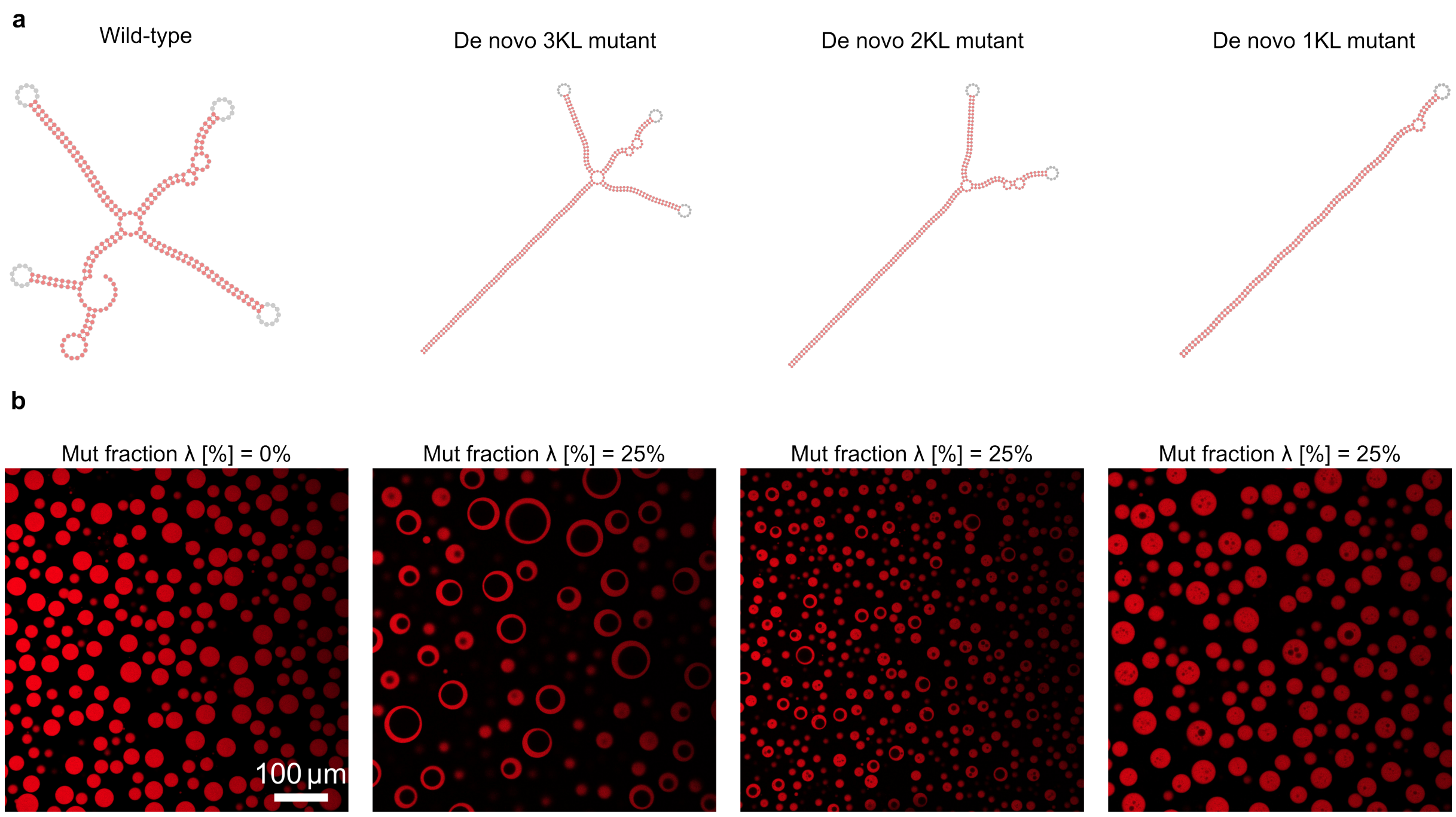

Response Fig. 1: **De novo mutant designs induce vacuole formation.** **a** Predicted secondary structures of the wild-type (WT) RNA nanostar (left) and three de novo designed mutant nanostars (3KL-1KL Mut). The mutants were designed to preserve the characteristic secondary-structure motif identified for the most abundant mutants in the sequencing data (Fig. 4 and Supplementary Fig. 14). The secondary structure of the mutant used for the mechanistic model, shown in Fig. 4 of the main text, corresponds to the 3KL mutant. **b** Representative confocal images of RNA condensates formed upon co-transcription of the main WT band with each de novo mutant template (at mutant fraction λ of 25%), showing robust vacuole formation for all three de novo designs. All nanostars (red) contain the malachite green aptamer and droplets are labelled with malachite green dye (λ_{ex} = $640\,\mathrm{nm}$). Scale bar, $100\,\mu\mathrm{m}$.

Comment 2: *"The authors propose that vacuole formation originates from a local spinodal decomposition. It would be helpful if the authors could justify why spinodal demixing is the favored pathway here rather than classical nucleation. In large droplets, spinodal decomposition may typically typically produces patterns with a characteristic length scale at early times (competition between molecular interaction and interfacial cost). Is such an early-time length scale observable? Relatedly, does vacuole coarsening follow a $t^{1/3}$ scaling? Could a dilute "vapour-like" RNA phase inside a dense RNA phase also be considered? Do the relaxation dynamics of two interacting vacuoles provide clues about interfacial tension or osmotic pressure differences?"*

Let us discuss the spinodal instability first. The spinodal instability is triggered by composition gradients inside the droplet that form due to the transcription reaction of RNA nanostars. Once the nanostar composition crosses the spinodal line in the phase diagram, a vacuole forms, which grows to the mature vacuoles observed experimentally. Accounting for the presence of noise enables slightly weaker composition gradients to stochastically nucleate vacuoles but does not affect the principle of vacuole formation. However, noise will slightly shift the onset of vacuole nucleation, and due to the sequential vacuole growth, mature vacuoles will form at different times and positions within the droplet. Thus, a regular spinodal pattern is easily disrupted by the noise while not noticeably affecting the onset of the instability.

We note that the experimentally considered droplets have a size in the order $10\,\mu\mathrm{m}$ to $100\,\mu\mathrm{m}$. Thus, such droplets contain roughly $(10^8 - 10^{11})$ nanostars, making the composition fluctuation on the scale of the concentration gradient negligible. Consistently, vacuole growth appears very deterministic rather than stochastic (see Supplementary Video S2) while vacuoles are nucleated at different times and positions.

The reviewer also asks about the early-time dynamics as well the late time coarsening dynamics. Experimentally, we observe that small vacuoles diffuse very quickly and fuse early on (see Supplementary Video S2). However, late-time coarsening is difficult to access due to the very slow diffusion of nanostars inside the droplet. On the time scale of the experiment of two days, single vacuoles still grow and have not (yet) transitioned to a clear Ostwald-like coarsening phase in which they compete with other vacuoles. Addressing the late-stage coarsening is an exciting question; however, it requires a new design of weaker kissing loops to significantly increase molecular diffusivity inside the droplets, which is beyond the scope of the current work – but we are looking forward to take this suggestion on for follow-up work.

Indeed, vacuoles can be considered as RNA nanostar poor phases ("vapour-like"). The dynamics of fusion between vacuoles could be used to assess the surface tension and viscosity of the RNA-rich phase. However, for such a purpose, studying the droplets without a vacuole likely represents the system of choice. Again, this is a great suggestion for a follow-up study.

Comment 3: *"The mechanism depends on slow internal mixing relative to surface-driven material input. This condition is specific to the present RNA system and may not hold in protein condensates where exchange and turnover are faster."*

We thank the reviewer for highlighting this important point. We agree that our proposed mechanism requires that internal mixing in the dense phase is slow compared to the rate of material input at the droplet surface (here, RNA production by transcription). We now stress in the Supplementary Information that this condition is fulfilled for our system and not necessarily for all biomolecular condensates.

At the same time, slow internal diffusion/transport is not unique to RNA droplets. A number of protein condensates have been shown to display significantly slower dynamics with high viscosity, even viscoelasticity and aging (up to dynamically arrested or glass-like states), in which internal rearrangements can become very slow[1]. Likewise, vacuoles have been reported in protein-rich condensates, including cases where limited diffusion in the dense phase plays a central role[2]. In a nutshell, the rate of change of the material input k (due to e.g. a change in temperature[2], the rate of transcription (our work), ...) has to be compared to the molecular diffusivity inside D_{in}. For sufficient gradients to appear, the corresponding reaction-diffusion length scale $\sqrt{D/k}$ has to be on the order of (or smaller than) the condensate size, which is actually the case for many condensates in the presence of perturbations and chemical processes. We now stress this aspect in the revised discussion of our manuscript.

Comment 4: *"The movies raise additional questions about vacuole dynamics: - How does the presence of vacuoles influence droplet fusion? - Why do multiple vacuoles often coexist instead of coalescing into a single interior cavity? - Is their a Oswald ripening-like mechanism driving the dynamics of vacuoles? - Some vacuoles tend to disappear: are they simply out of focus or do they actually dissolve/shrink?"*

We thank the reviewer for raising these interesting questions regarding vacuole dynamics observed in the movies.

We have not systematically assessed how the existence of vacuoles affects droplet fusion on a material property level. However, we find that vacuoles lead to an increased movement of the droplets (Fig. 5 in main text), which in turn increases the number of coalescence events and thus droplet size (Fig. 1f in main text).

In our view, the long lifetime of vacuoles is most naturally explained by the highly viscous nature of the RNA-rich phase, which strongly slows down internal rearrangements. As a result, interfacial relaxation and vacuole–vacuole coalescence proceed extremely slowly, allowing multiple vacuoles to persist for many hours.

To examine this more directly, we studied the bright-field movies of droplets and vacuoles. Compared to the confocal fluorescence timelapses, bright-field imaging provides a much larger depth of field, making it easier to assess whether vacuoles truly disappear instead of simply moving out of the confocal plane. Consistent with our theory, we observe only very few vacuole–vacuole fusion events over the duration of the 60 hour long movies. We now highlight representative examples in two new Supplementary Videos (Supplementary Videos S3 & S4): one showing the merger of two small vacuoles, and another showing fusion involving a large vacuole. These observations support the interpretation that vacuoles disappearing in fluorescence

confocal timelapses, most often arises from fusion with another vacuole or from motion out of the confocal plane, rather than rapid dissolution.

Regarding potential Ostwald ripening effects, over the timescale of our experiments, vacuoles tend to persist and, when visible, generally increase in size rather than shrinking, as required for coarsening. We discuss this point and its limitations in the revised manuscript.

Comment 5: *"The proposed mechanism of droplet motility is that vacuole growth generates internal mass redistribution and displaces the center of mass, leading to droplet motion. This is plausible, but the current explanation is a bit short. Is their any analogy with self-propeled droplets in active systems?"*

We agree that the explanation for the motion in the main text was too brief and we have expanded it. Mut droplets show significantly enhanced motility compared to WT droplets, whose dynamics are consistent with Brownian motion ($P < 0.001$, Fig. 5c). We propose that this motion arises from internal mass redistribution caused by vacuole nucleation and growth. Vacuoles remain stable over multi-hour trajectories, and because the vacuole-to-droplet volume ratio can be high, their formation produces a substantial redistribution of mass within the droplet. Crucially, vacuoles do not always nucleate at the droplet's center; asymmetric nucleation and growth shift the droplet's center of mass and thereby generate displacement.

Because new vacuoles nucleate at essentially random positions, this process does not establish a persistent polarity or propulsion axis as in self-propelled droplets[3]. Instead, each nucleation-and-growth event produces an intermittent, stochastic shift of the center of mass, which we expect to manifest as an increased effective translational diffusivity. Transcription continues after vacuoles first form (roughly 6 h after the start of transcription, see Supplementary Video S2 and Fig. 1f), so new droplet material and new vacuoles are continuously generated. Consequently, droplets can move, pause, and move again (Supplementary Fig. 23). Importantly, while the mechanism is rooted in symmetry breaking, our system differs from previously reported self-propelled droplets in that symmetry breaking is not driven by chemical turnover, surface-tension gradients, or external chemical gradients[3].

Reviewer #2

"The article by Vestraeten et al. presents a system of RNA-based synthetic condensate protocells that are under active control of a promoter, i.e. in which the RNA that constitutes the condensate is created in situ by transcription from a DNA template. While this has been done before, the novelty here lies mainly in the introduction of mutations in the DNA template that create mutant RNA species that alter the behavior of the droplets, most notably through creation of vacuoles. The authors characterize the mutant RNA by sequencing, then construct mimics of the mutant/wild type mixture that are able to reproduce the vacuole phenotype. They present a theory to explain the vacuole formation, and show that vacuole formation leads to novel droplet

behaviors (enhanced diffusion, compartmentalizing other solutes). Overall I found this to be a highly creative article. I agree that the work described has taken a significant step towards the creation of a DNA droplet system capable of some sort of evolution, though the present article falls well short of actually achieving that. I also appreciate that they brought a range of new techniques to bear on the field of nucleic acid condensates. The introduction of mutagenesis, and the use of sequencing to characterize the RNA, are very nice methodological advances. The specific results are interesting, albeit slightly confusing mechanistically (see below). I am tentatively inclined to be in favor of publication, though I have multiple queries of varying importance:"

We thank the reviewer for the positive feedback on our work ("creative article", "nice methodological advances"). We appreciate the thorough review and the time the reviewer put into reading our work and making helpful suggestions to improve our manuscript.

Comment 1: *"The discussion of 'prerequisites for evolution' in the introduction is interesting, but points (ii) and (iii) are hard to distinguish; i.e. they seem to say the same thing. Thus it is not obvious how the experiments have separately achieved (ii) vs. (iii). Can this be clarified? Also, I would think there is origin of life literature discussing definitions of minimal evolvable entities, but the definition (i,ii,iii) is not referenced; this should be rectified."*

We thank the reviewer for this helpful suggestion. We agree that points (ii) and (iii) were not stated in a sufficiently distinct way, and we have revised the Introduction to clarify their different roles.

In the revised text, we more explicitly distinguish (ii) genotype-dependent phenotypic variation from (iii) the emergence of non-trivial, potentially selectable phenotypic traits. Specifically, (ii) refers to the requirement that changes in genotype (mutations in the DNA template) lead to changes in droplet phenotype, i.e. that the system supports heritable phenotypic variation once genotype–phenotype coupling is in place. By contrast, (iii) is a stronger requirement: it denotes that mutagenesis can generate functional phenotypic features and increased complexity (rather than only loss-of-function outcomes, e.g. disrupted droplet formation, as shown in Fig. 3 for high mutational rates). This distinction is important because a system may satisfy (ii) even if mutations are purely destructive, while (iii) captures the appearance of emergent droplet-level traits that could, in principle, underlie differential performance under selection.

We also agree that our original (i–iii) framing should be referenced throughout the text. We have therefore added citations to classic formulations of the minimal conditions for Darwinian evolution (genetic encoding, variation and differential fitness)[4,5], as well as origin-of-life/protocell literature discussing minimal evolvable entities and requirements for protocells and Darwinian evolution[5–8].

Comment 2: *"It is mentioned in the intro, and clear in the supplementary videos, that the vacuoles are extraordinarily stable, persisting in close proximity without merging (fusing) for up*

to 10 hours. Indeed it was hard for me to identify more than one or two vacuole fusion events (out of ∼100s of vacuoles) in the very long supplementary videos. This is hard for me to understand without some specific stabilization mechanism. I am curious if it might be the case that the Mut RNA might act as a surfactant that stabilizes the vacuoles. There is mention of separate fluorescent labeling of the Mut and WT RNA; can these be imaged in different colors to see if there is accumulation of the Mut on the interface?"

We thank the reviewer for this insightful suggestion. A surfactant-like stabilization of the vacuoles is a plausible mechanism for long-lived hollow condensates, and such effects have been discussed previously[9]. We therefore directly tested whether the mutant RNA enriches at the vacuoles' interfaces.

To this end, we imaged WT and Mut nanostars in two colors by encoding WT with the malachite green aptamer (red) and Mut with the Broccoli aptamer (cyan). To ensure that the system was at equilibrium we annealed mixtures of purified WT RNA (63%) and Mut (37%) nanostars inside water-in-oil droplets. If Mut nanostars acted as a surfactant, we would expect preferential accumulation of the Mut signal at the droplet interface, accompanied by a depletion in the droplet interior. However, the confocal images (Response Fig. 2a) and the corresponding line profiles (Response Fig. 2b) show that both WT and Mut fluorescence are broadly homogeneous across the droplet cross-section, without systematic enrichment of mutant signal at the interface. These experiments therefore do not support a surfactant-like role of the Mut-nanostars (although we fully agree with the reviewer that this may have been an intuitive interpretation of our results).

We currently attribute the long lifetime of vacuoles primarily to slow dynamics: the RNA-rich phase is highly viscous and internal rearrangements are strongly hindered, which suppresses interface relaxation and vacuole coalescence on experimental timescales. We have added this control experiment to the SI Supplementary Fig. 21 and the corresponding discussion to the main text of the revised manuscript.

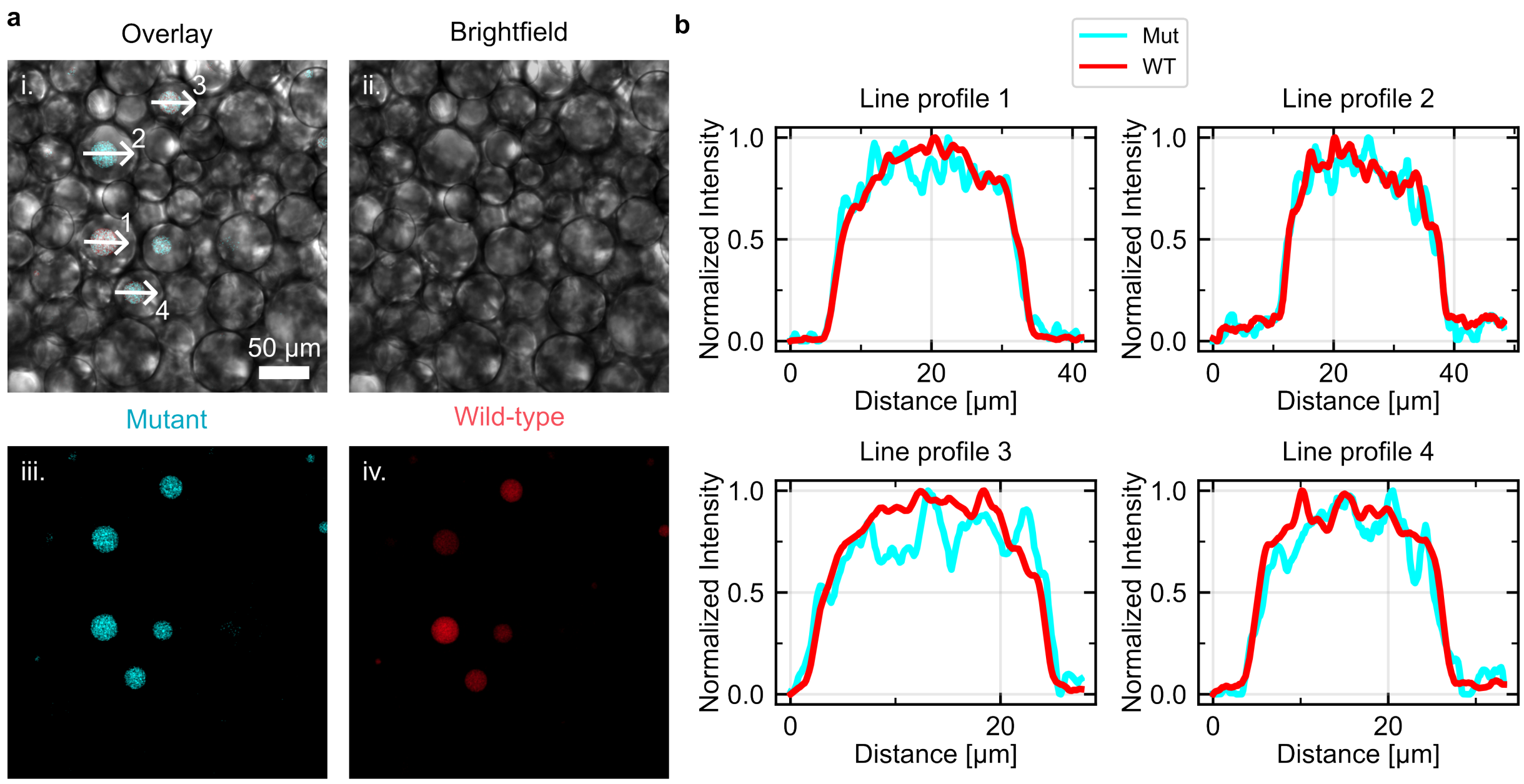

Response Fig. 2: **Confocal imaging of WT and Mut nanostars to test whether Mut acts as a surfactant. a** Representative confocal image of condensates formed by annealing purified WT and Mut RNA nanostars inside water-in-oil droplets. WT nanostars (red) contain the malachite green aptamer and are labelled with malachite green dye (λ_{ex}=640 nm), Mut nanostars (cyan) contain the Broccoli aptamer and are labelled with DFHBI-1T (λ_{ex}=488 nm). Arrows indicate lines used for line profiles. **b** Normalised intensity profiles extracted from the droplets marked in **a**, comparing WT (red) and Mut (cyan) fluorescence across the droplet. Both signals are broadly homogeneous across the droplet cross-section and no systematic enrichment of Mut at the droplet interface is observed. Scale bar, 50 µm. All droplets shown are from a single field of view from a single experiment. Mut nanostars do not accumulate at the interfaces, which suggests that they do not act as surfactants.

Comment 3: *"A general issue is that the system is much more complex than just a mixture of RNA species. Can the authors comment on the potential role of the RNA polymerase (which after all has DNA and RNA binding abilities), or the DNA template, in affecting the mixture and/or the droplet morphology? E.g. what happens if purified nanostars are mixed without polymerase or DNA present? I could imagine an argument that the concentrations of these other species so low as to not affect mixing or morphology, but if this is the stance of the authors, it should be made explicit."*

We thank the reviewer for raising this important point. We agree that, in principle, additional components in the transcription reaction (DNA template, RNA polymerase and buffer components like nucleotides) could influence the phase behaviour and/or droplet morphology.

Our mechanistic model is explicitly an active one: the vacuoles arise from transcription-driven

kinetics (continuous RNA production leading to concentration gradients within droplets), rather than from the equilibrium mixing of a fixed composition of RNA species. Therefore, simply mixing purified nanostars post-transcription (i.e. in the absence of ongoing RNA production) is not expected to reproduce the vacuolation dynamics described here, because the key non-equilibrium driving (transcription) is removed.

That said, we agree it should be stated explicitly how the DNA and RNA polymerase contribute to the material properties of the dense phase. In our experiments, both species are present at very low concentrations compared to the RNA (DNA template in the ngμL^{-1} range) and thus we expect them to have a negligible contribution to the bulk mixing thermodynamics and viscoelastic properties of the RNA-rich dense phase. We have added these points to the revised manuscript to make this assumption explicit and to clarify that the DNA template and RNA polymerase are treated as catalytic components that enable the active production of RNA nanostars, rather than as major droplet-forming constituents.

Comment 4: *"I found it very interesting that the DNA template was engulfed within the RNA droplets, but I found the article to not use that fact in certain interpretations. First, is there any correlation between the location of vacuole appearance and the location of DNA clusters? Second, the model explaining the vacuole appearance assumes that most of the new RNA is formed outside of the droplet, which seems to contradict the fact that most of the template is inside in the droplet. Actually, I am not sure if 'most' of the template is inside; the images and discussion just indicate a lot is inside, but I take the point in the supplement that most of the volume is outside. So I suppose there are two related questions: i) can the authors make this all a bit more quantitative, e.g. by estimating the partition coefficient of DNA template in/out of the droplets (perhaps from fluorescent confocal measurements) and/or commenting on the volume ratio in/out of condensed phase; ii) does such an estimate support the assumption that most RNA is formed outside the droplet?"*

We thank the reviewer for these thoughtful questions and agree that the manuscript would benefit from a clearer, more quantitative discussion of DNA localization and how this relates to our modeling assumptions.

First, while we consistently observe that DNA clusters co-localize with the RNA-rich condensates (Response Fig. 3a), we do not find evidence that vacuole nucleation is spatially correlated with the DNA. In particular, in the dual-channel confocal images the DNA signal is not consistently enriched at vacuole interfaces (Response Fig. 3b), which we would expect if vacuoles formation was triggered at the DNA clusters. We therefore interpret DNA encapsulation as a robust feature of co-transcriptional assembly, but not as a direct spatial determinant of where vacuoles form.

Second, we agree that the statement "most RNA is formed outside of the droplet" requires clarification in light of the observation that a fraction of templates is retained within droplets. Two points are relevant here. (i) Although DNA is clearly present inside droplets, our data do not allow a reliable estimate of ratio of the DNA concentration in the dilute phase because the

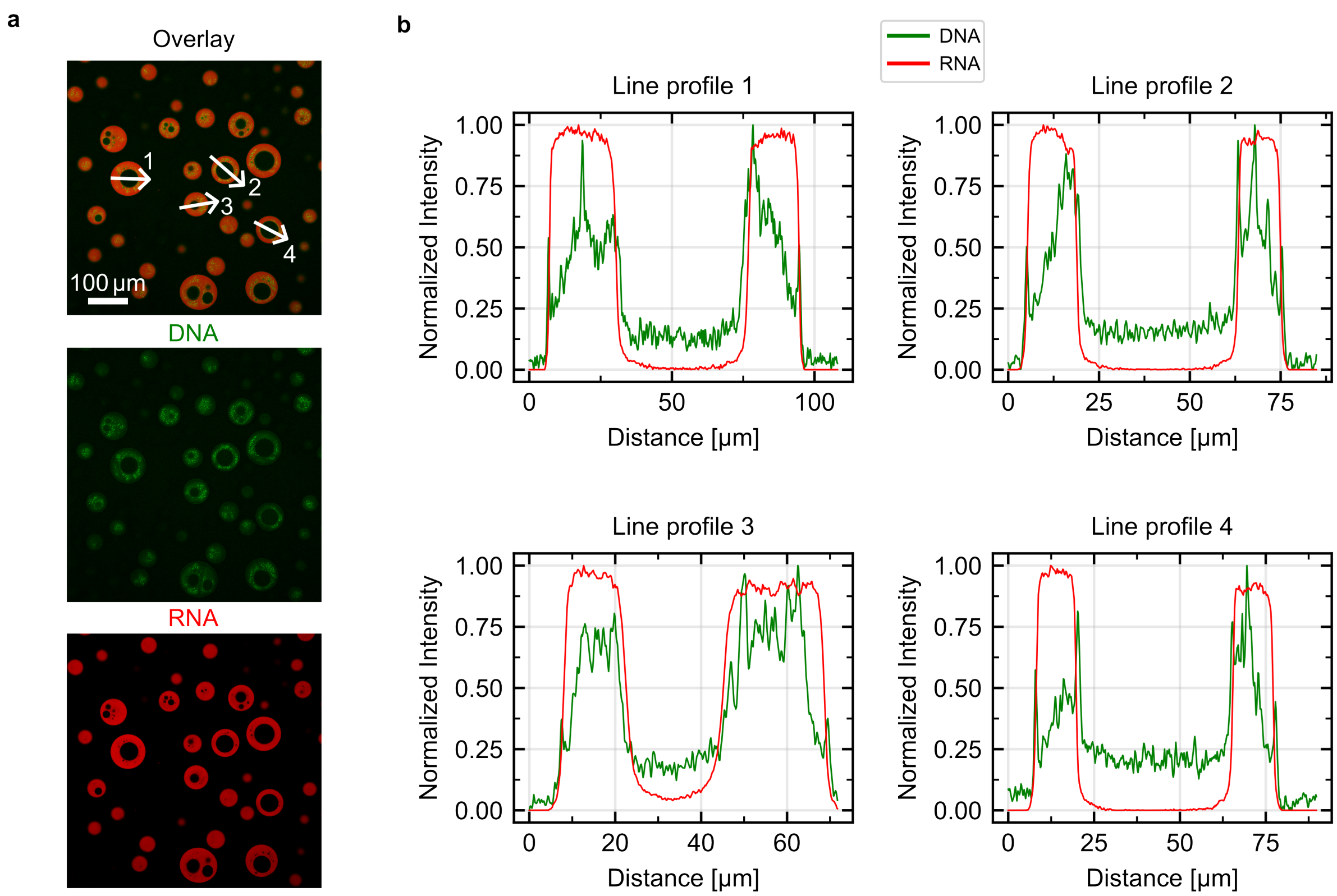

Response Fig. 3: **Co-localization of DNA templates with RNA droplets. a** Representative confocal images of RNA droplets (red) formed by in vitro transcription from fluorescently labelled DNA templates (green). RNA droplets contain the malachite green aptamer and are labelled with malachite green dye (λ_{ex}=640 nm), DNA templates are labelled with fluorescein-dUTP (λ_{ex}=488 nm). Overlay shows co-localization of DNA with the RNA-rich condensates, consistent with encapsulation/retention of the DNA template during co-transcriptional droplet formation. Scale bar, 100 µm. **b** Line profile analysis of DNA fluorescence relative to the RNA fluorescence signal across droplets confirms that the DNA signal is localized within the condensate interior and does not show systematic enrichment at the interface of vacuolated droplets. We also cannot observe DNA templates inside of the vacuoles (although this may be due to low signal when the aggregates are dissolved in bulk solution).

out-of-droplet signal is close to the background and influenced by imaging limitations (fast diffusion of the DNA template in the dilute phase and low background to noise ratio). A robust partition coefficient would therefore require additional dedicated experiments and calibration standards, which we consider beyond the scope of the current revision and better suited for future work.

(ii) Independent of DNA partitioning, we can estimate the fraction of total volume occupied by the condensed phase from the droplet size distribution and droplet number density in a typical field of view. We have added the details of this analysis to section 1.13 of the Supplementary Information. This analysis indicates that only a small fraction of the total sample volume is occupied by dense phase (of order $\sim$2% under our standard conditions). Because the dilute phase constitutes the vast majority of the reaction volume, it can dominate total RNA production even if a subset of templates is encapsulated. Moreover, the RNA-rich condensed phase is highly viscous, and we therefore expect that diffusion-limited supply of NTPs and slower transport of transcription components can further reduce the effective transcription rate inside droplets compared to the dilute phase.

Taken together, these considerations support our modeling assumption that the dominant contribution to RNA production occurs in the dilute phase outside droplets, while allowing that a fraction of transcription can occur from templates retained within droplets. We have revised the text to clarify this point, added the volume-fraction estimate, the new figure as Supplementary Fig. 7, and toned down the wording to avoid implying that all templates reside inside droplets.

Comment 5: *"Droplet areas are used throughout the paper to quantify the amount of condensed phase. Area can be strongly affected by wetting, so it would be useful to show a confocal stack showing whether the droplets are wetting their container (the glass surface)."*

We thank the reviewer for this helpful suggestion. We agree that the implications of using droplet area to estimate the droplet size can be biased by surface wetting. To address this, we now include a representative confocal z-stack with orthogonal views (XZ/YZ) to directly assess whether droplets wet the glass surface (Response Fig. 4).

The z-stack indicates that droplets remain largely spherical and do not display pronounced spreading on the glass, suggesting that wetting is not a major contributor under our imaging conditions. We do observe a slight flattening for larger droplets, consistent with partial gravitational settling/collapse rather than extensive wetting. We have added the z-stack figure to the SI (Supplementary Fig. 3) and an explicit statement to the Methods/Results of the revised manuscript to clarify that droplet areas used throughout the manuscript primarily reflect droplet size and are not subjected to strong wetting of the glass surface.

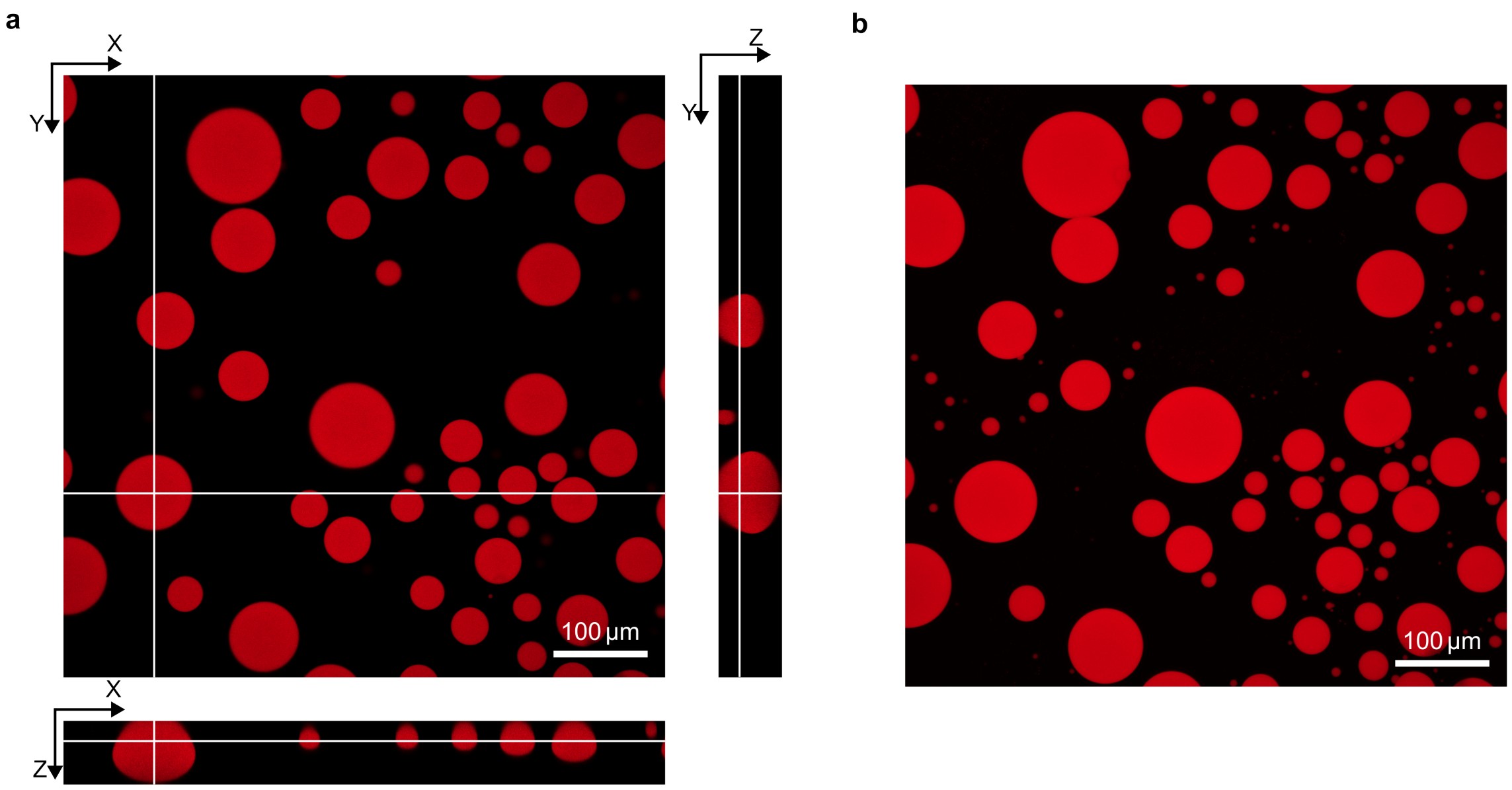

Response Fig. 4: **Confocal z-stack to assess droplet wetting.** **a** Representative confocal z-stack of WT RNA droplets (red), droplets contain malachite green aptamer and are labelled with malachite green dye (λ_{ex}= $640\,\mathrm{nm}$). The orthogonal views XZ (bottom) and YZ (right) are shown to visualize the droplet shape relative to the glass surface. The first frame was taken on the glass surface (z=0). The droplets are predominantly spherical, with only mild flattening consistent with gravitational settling rather than strong surface wetting/spreading. White cross-hairs indicate the position of the orthogonal slices. The stack spans a total height of $67.54\,\mu\mathrm{m}$ and contains 98 slices; the representative XY view shown corresponds to slice 33/98. **b** Maximum-intensity projection of the confocal z-stack shown in **a**. Scale bars, $100\,\mu\mathrm{m}$.

Comment 6: *"The explanation of the spinodal decomposition relies on a materials property, i.e. the slow diffusion of nanostars in the dense phase, which they support by reference to other works. This seems fine to me, except I think it would be useful to add at least some direct estimate of materials properties to the paper. Given the videos, it seems straightforward to estimate the inverse capillary velocity from the timescale of droplet fusion events. It would be helpful to report this value, so that the behavior of this particular system can more directly be compared to other systems."*

We appreciate the reviewer's suggestion. Using fusion events in the time-lapse videos of WT and Mut RNA droplets (Response Fig. 5a-c), we estimate a capillary velocity on the order of $\sim 10^{-2}\,\mu\mathrm{m\,s^{-1}}$, consistent with values reported previously for RNA nanostar condensates[10].

In the SI section 2, we now discuss the proposed measurement of the capillary velocity, which is about $\sim 10^{-2}\,\mu\mathrm{m\,s^{-1}}$, and thus consistent with previous estimates for RNA droplets[10,11].

For reference, this is about 100 fold lower than P granules (biomolecular condensate in the C. elegans embryo)[12,13].

In addition, we performed FRAP measurements on WT RNA droplets labelled with fluorescein-rUTP (Methods in Supplementary Section 1.4, Response Fig. 5d,e). We observe no significant recovery of the droplet material even 10 minutes after bleaching. Using these results and other references from literature[14], we estimate the diffusivity $D^{\text{in}} \simeq 5 \cdot 10^{-3} \mu m^2/s$ and viscosity $\eta \simeq 1 Pas$ inside. Via the capillary velocity we find a surface tension $\gamma \simeq 10^{-2} \mu N/m$. These estimates and the associated discussion are now included in the revised Supplementary Information (Section 2.2-2.4 and as new Supplementary Fig. 6) and referenced in the main text.

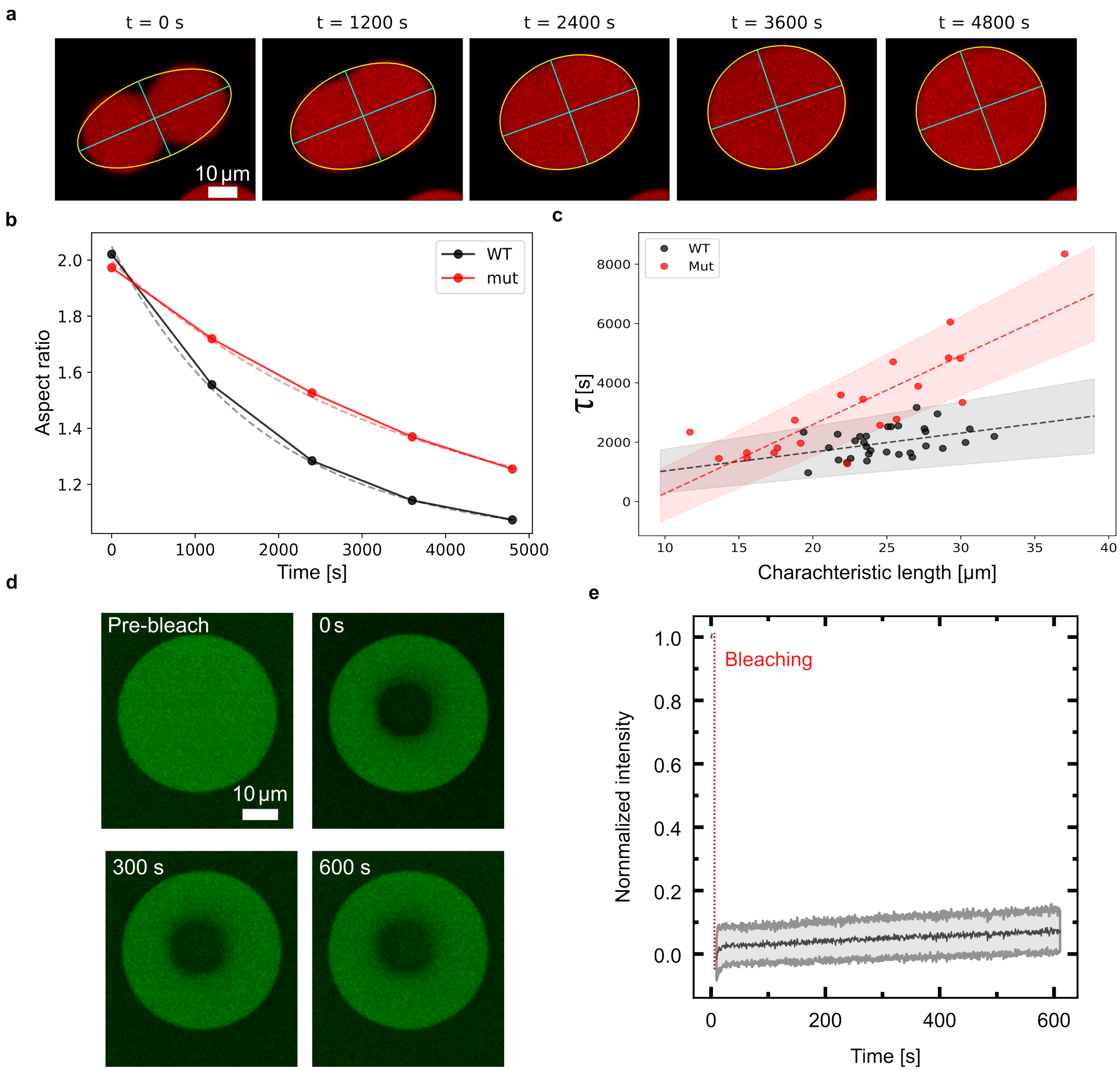
a
t = 0 s
t = 1200 s
t = 2400 s
t = 3600 s
t = 4800 s
10 µm
b
Aspect ratio
Time [s]
WT
mut
c
WT
Mut
τ [s]
Charachteristic length [µm]
d
Pre-bleach
0 s
300 s
600 s
10 µm
e
Bleaching
Normalized intensity
Time [s]

Response Fig. 5: **Quantification of the material properties of RNA droplets.** **a** Schematic illustration of the relaxation dynamics of RNA droplets used to quantify the capillary velocity. $v = \frac{\gamma}{\eta}$. **b** Example of the measured aspect ratio over time for a WT (black) and Mut (red) droplet. **c** Fitted inverse capillary velocity for Mut (red) and WT (black) droplets. Inverse capillary velocity for WT (2 replicates, N=31), v^{-1} = 63.70 ± $27.12\,\mathrm{s\,\mu m^{-1}}$ and for Mut (2 replicates, N = 20), v^{-1} = 231.96 ± $34.85\,\mathrm{s\,\mu m^{-1}}$. Hence for both condensates, $v \sim 10^{-2}\,\mathrm{\mu m\,s^{-1}}$ **d** Example of WT RNA droplet before and after bleaching, showing negligible recovery even after 10 minutes. Scale bar, $10\,\mathrm{\mu m}$. **e** Quantification of the fluorescence recovery for WT droplets (n = 2, T = $37\,^{\circ}\mathrm{C}$). Data was normalised to intensity before bleaching and background to account for background fluorescein signal.

Comment 7: *"While I applaud the creativity in creating two different types of mutants (through duplication/deletion in Fig 1, then the Fig. 3 substitutional approach), I will admit I found the switch to not be clearly motivated. If possible, it would help to add a few words clarifying the benefits of the two approaches, or the need to switch to a second method."*

We thank the reviewer for this comment and agree that the motivation for moving from the insertions/deletions mutants (Figs. 1 and 2) to the substitution-based approach (Fig. 3) was not explained clearly enough.

In brief, the two mutagenesis routes serve different purposes. The PCR byproduct route (Figs. 1 and 2) yields a small number of mutant classes at relatively high abundance, which makes it feasible to identify representative sequences, reconstruct a defined mutant, and use it for quantitative, mechanistic modeling (Fig. 4). To strengthen this part, we have added a de novo design variant upon request of the first reviewer (Response Fig. 1, new Supplementary Fig. 15). By contrast, the substitution/error-prone approaches in Fig. 3 generate a much broader diversity of mutants (each at low individual abundance), so they are not intended for sequence-level reconstruction; instead, they provide a widely used, general mutagenesis strategy (applicable to essentially any DNA sequence) with a tunable mutation rate. This allows us to test how vacuole formation depends on mutational load and to identify the functional window where vacuoles appear (and could essentially appear in a mutagenesis process in nature), as well as the loss-of-function regime at higher mutation rates.

We have added a short explanation clarifying this rationale to the revised manuscript.

Comment 8: *"After much study, I believe (possibly naively) that I came to understand the model that is fleshed out in the supplement, and commented only briefly in the main text. Ultimately I feel that the authors should consider revising the main text paragraph to get the point more clearly across. However, let me try to re-state what they are proposing: just before phase separation occurs, the Mut/WT ratio in the homogeneous system is λ. Then phase separation occurs; the total ratio is still λ, but the actual ratio in each phase is no longer λ (being instead whatever ratios are found at the ends of the tie lines). But the transcription continues, driving (according to the assumption I asked about above) the dilute phase towards λ, which is not*

in equilibrium with the conditions inside the dense phase. This gives rise to the gradient and the spinodal decomposition. Is this the explanation? If so, then I wonder if adding some clear comments in the main text about the key issue is that initial equilibration at a bulk ratio of λ is later driven out of equilibrium by active maintenance of the dilute solution at ration λ is better explained."

The reviewer summarizes well the key aspects of the mechanisms underlying the spinodal instability, eventually leading to the vacuoles. We agree that the main text was rather condensed to enable readability for a broad readership. Based on the reviewer comments, we have now revised the paragraphs on the theory and now stress the mechanisms in more detail. We appreciate this suggestion and the time the reviewer has put into digging into the model in detail.

Comment 9: *"Some clarification of timing would be useful. The methods indicate transcription was allowed to proceed for 48 hours before imaging; is this really how data like Fig. 1d and 1e were acquired? Specifically, those panels show time points of 1h, 4h, etc; do those refer to 1h, 4h, etc., after the start of transcription, or 49h, 53h after starting? Generally the very long times of the experiments are notable; is the RNA polymerase still active and transcribing throughout all this time (i.e. over multiple days)? Does the system run out of nucleotides?"*

We thank the reviewer for raising this point and agree that the timing was not described clearly enough. In the majority of experiments, samples were imaged after 48 h of transcription (as stated in the Methods). However, Figs. 1d and 1e are time-course experiments: imaging was initiated right after the start of transcription and continued for up to $60\,\mathrm{h}$. Accordingly, the time points shown in these panels ($1\,\mathrm{h}$, $4\,\mathrm{h}$, etc....) refer to the elapsed time since the start of transcription (i.e., 1–48h), not $49\,\mathrm{h}$, $53\,\mathrm{h}$, etc... We have revised the figure captions (and corresponding text) to make this more explicit.

Regarding the long experiment durations, RNA polymerase activity can persist over extended times under our reaction conditions. According to Ref. [15], when rNTPs deplete, the T7 polymerase enters the rNTP-limited kinetics and thus slows down for rNTPs concentration below $\sim 50\,\mu\mathrm{M}$. Using a sequence transcription rate of 1/sec and a DNA concentration of $45\,\mathrm{nM}$, we expect that the T7 polymerase enters the rNTP-limited regime after $60\,\mathrm{h}$. We conclude that when vacuoles form, the T7 polymerase is active at rNTP excess.

Reviewer #3

"The authors showed that cotranscriptional condensation of RNA nanostars, which self-assemble via kissing-loop interactions, encapsulate their own DNA templates. By introducing mutations into the DNA templates, they showed that mutant nanostars drive the formation of long-lived vacuoles within the droplets. Theoretical modeling suggested a plausible mechanism for vacuole formation. Finally, the authors showed that these nonequilibrium systems give rise to spon-

taneous droplet motion and can be used for cargo sequestration. Taken together, the authors suggest that these systems exhibit several essential features of minimal synthetic cells. In my opinion, the interesting results that are presented in this manuscript could be presented more clearly, both through specific clarifications and also potentially a reorganization of the document. I highlight primary areas of concern below:"

We thank the reviewer for the assessment that our results are interesting. We appreciate the feedback and have used it to enhance the clarity of manuscript.

Comment 1: *"The authors emphasize at the outset that RNA droplets encapsulate their DNA template upon formation, but it is not clear from the main text whether this must always be the case or whether this phenomenon is specific to the system studied here. Furthermore, the mechanistic reason for this encapsulation is not addressed."*

We thank the reviewer for raising this important point. In our experiments, co-localization of the DNA template with the RNA-rich condensate is a robust feature under the co-transcriptional assembly conditions used here. We do not intend to claim that DNA encapsulation must occur in all RNA droplet systems; rather, we view it as a consequence of the way in which RNA nanostars fold and assemble while being transcribed from a DNA template. We have clarified this scope in the revised manuscript, and we expect this coupling to be a general feature for RNA nanostar droplets that form co-transcriptionally from DNA templates under similar conditions.

Mechanistically, our working hypothesis is that nascent nanostar RNA begins to fold and engage in multivalent interactions co-transcriptionally while still physically connected to the DNA via the RNA polymerase. Importantly, we expect DNA encapsulation to occur only after sufficient RNA has accumulated for phase separation to take place. At that point, a subset of actively transcribing templates can become captured by the forming RNA-rich network through this transient tethering, whereas other DNA molecules in solution need not become encapsulated. Consistent with the volume of the dilute phase being much bigger than the RNA condensates' volume, we expect that most transcription occurs in the dilute phase outside the condensate, with only a fraction of templates becoming incorporated once droplets have formed. As above, directly verifying the detailed sequence of capture events and the spatial organization of DNA/polymerase/nascent RNA during growth would require substantially more complex experiments beyond the scope of the present work, and we now frame this as a plausible mechanistic interpretation in the revised manuscript. We have made our hypothesis on the mechanistics of DNA encapsulation more clear in the revised manuscript.

Comment 2: *"The explanation of mutagenesis via PCR (second paragraph of the Results) is very brief given the importance of this to the subsequent results. A schematic figure in the main text that shows how self-complementary RNA act as primers would be helpful. Also, does PCR imply that the temperature is cycled, and if so, what happens to the droplets during this process?"*

We agree that our description of the non-specific PCR mutagenesis was too brief given its importance. We have therefore added a schematic figure (Response Fig. 6), due to space constraints it is included in the Supplementary Information (Supplementary Fig. 2) rather than the main text. The schematic clarifies that the non-specific products arise because the WT DNA contains self-complementary regions that can enable primer-independent template–template priming during the annealing step, generating longer amplicons, and because partial complementarity can lead to mispriming at internal sites, generating shorter amplicons.

Importantly, PCR in our workflow refers to amplification of the DNA template only. This step is performed prior to in vitro transcription and droplet formation. Droplets are therefore not present during temperature cycling and are never exposed to PCR conditions. After PCR, the resulting DNA pool is purified and then used as the template for transcription at constant temperature ($37\,^{\circ}\mathrm{C}$), where RNA production and droplet formation occur.

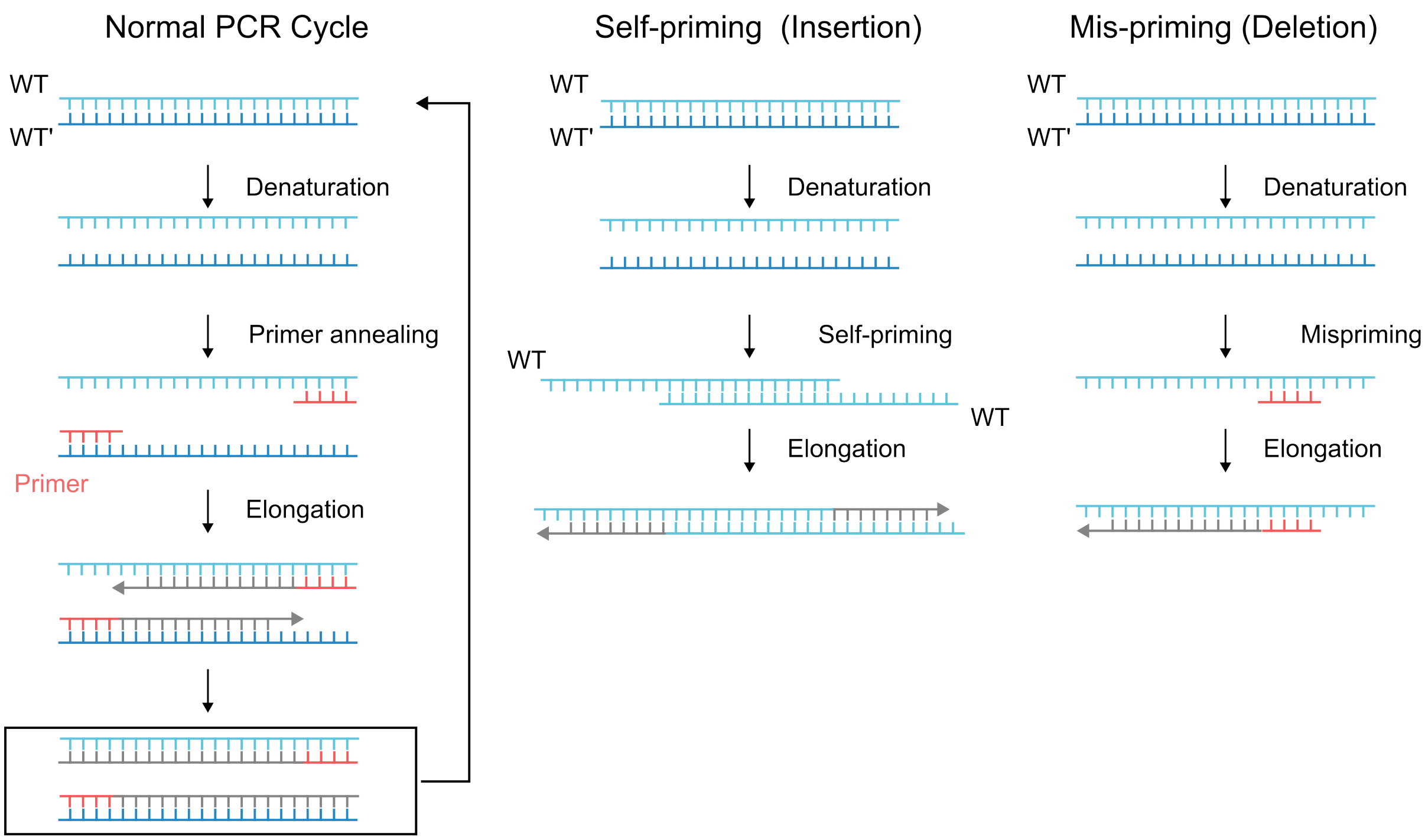

Response Fig. 6: **Schematic showing how non-specific PCR products can arise during amplification of the WT DNA template.** PCR proceeds through repeated cycles of denaturation (strand separation), annealing, and extension. After denaturation, the annealing step can occur via the intended binding of the forward and reverse primers to the template ends (left), followed by polymerase extension to yield the expected full-length amplicon. Alternatively, because the WT template contains self-complementary regions, partially annealed template strands can act as primers for one another (intermolecular self-priming; middle), and subsequent extension generates longer, duplicated/extended products. In addition, primers can anneal non-specifically to the DNA with partial complementarity (mis-priming; right), leading to extension from an incorrect start site and thus truncated (shorter) amplicons. Red segments indicate primers; grey arrows indicate polymerase extension; dotted regions indicate imperfect/partial base pairing.

Comment 3: *"I found the explanation of the experimental results on various mutagenesis schemes to be confusing because the specific hypotheses that were being tested were not clear. Why did the authors choose to perform mutagenesis in these particular ways? Is the key conclusion from all of this simply that mutagenesis, essentially no matter the details, leads to vacuole formation? Perhaps I missed it, but I did not see how these experiments were guided by rational hypotheses or how they led to a mechanistic understanding."*

We agree that the rationale linking the different mutagenesis strategies to the hypotheses being tested was not stated clearly enough, and we have revised the text to make this explicit.

Our hypothesis is that different types of mutations can induce vacuole formation when transcribing RNA droplets, *i.e.*: deletion, insertions and substitutions. The different mutagenesis schemes were therefore chosen to test complementary aspects of this hypothesis, while also balancing generality versus tractability for mechanistic modeling:

(i) **Non-specific PCR byproducts (Figs. 1–2):** This route produces a *limited number* of mutant sequence classes that occur at comparatively high abundance (on the order of $\sim$5%), enabling (a) identification of mutants by sequencing, and (b) reverse-engineering of a defined mutant nanostar that can be characterized quantitatively. These experiments therefore provide the foundation for the mechanistic model in Fig. 4 of the main text (phase diagram, free-energy fit, and instability criterion). We note that this route is *not* a general mutagenesis strategy: the specific byproducts depend on the particular template/primers and amplification conditions, and thus are not readily transferable to arbitrary DNA sequences.

(ii) **Substitution mutagenesis with controllable mutation rate (Fig. 3):** In contrast, this approach generates a *high diversity* of mutants, each at lower individual abundance, which makes sequence-level identification and modeling not feasible. Its purpose is instead to test a distinct hypothesis: that vacuole formation depends on the *mutation rate* and that beyond a threshold mutational load the system loses the ability to assemble droplets ("loss of function"). Importantly, these substitution-based approaches (including cycle-controlled mutagenesis[16]) are widely used in directed evolution and mutation/selection experiments and are broadly applicable to essentially any DNA sequence, providing a general and transferable route to explore phenotype versus mutation rate[17,18].

Regarding the reviewer's question: the key conclusion is that vacuole formation is promoted over a finite window of mutational perturbation. Sufficiently low mutational load yields vacuolated droplets, and excessive mutational load disrupts droplet formation (loss of function), as shown in Fig. 3.

Finally, we agree that only a subset of the mutagenesis experiments directly informs the mechanistic understanding. We now state explicitly that the mechanistic model (Fig. 4) is built from the mutant class that can be isolated and reconstructed from the non-specific PCR route (Figs. 1–2), whereas the rate-controlled substitution mutagenesis and error-prone PCR (Fig. 3) are used to probe generality/robustness and to identify the functional regime in which vacuoles can emerge.

Comment 4: *"Although a mechanistic understanding is provided by the theoretical model, most of the details required to understand the explanation are contained in the supplementary information. In my view, it must be possible for the reader to understand the central conclusions of the theoretical model and how they relate to the experimental results in the main text. Several important aspects of the theoretical approach are omitted from the main text, including: Why is DNA not explicitly modeled in the free energy density? How do the sizes of the mutant and WT RNA enter into the theory, given that the main text only mentions the Chi parameters? If the spinodal is essential to the mechanism, why is it not shown on the phase diagram in the main text (the supplementary figures are much clearer)? In which direction do the concentration gradients*

go, and why does that lead to an instability (again, this is only clear in the supplement)?"

It is a subtle balance between the accessibility of the main text to a broad readership and a maximally thorough, detailed line of argument, typically involving the introduction of many technical steps as well. In the revised manuscript, we now follow the reviewer's suggestion and have extended the discussion of the spinodal instability mechanism and its link to the concentration profile within the RNA nanostar-rich droplets. We considered adding the spinodal and the associated concentration-profile schematic directly onto the main-text phase diagram, but doing so would require overlaying multiple additional elements (spinodal, profile arrows, illustrative gradients, and fit/measurement annotations), which would make the figure overly crowded and potentially ambiguous given that it would combine experimental data, the thermodynamic fit, and the kinetic-model interpretation. To keep the main text accessible for a broader audience, we have also expanded the SI to further enhance clarity.

About the reviewer's questions: The DNA is not explicitly modeled by the free energy used for the phase diagram because of its relatively low mass concentration compared to the RNA nanostars. Specifically, the DNA concentration is $45\,\mathrm{nM}$, while nanostars reach concentrations in the order of μM. The ternary Flory-Huggins model has five parameters: two molecular masses ($i =$ Mut,WT) and three interaction parameters. As the reviewer proposed, we also stress this aspect in the main text. Moreover, we agree that the spinodal line and concentration profiles are key to vacuole formation. Thus, we now provide a very detailed explanation of the mechanism to the SI.

Comment 5: *"How exactly is the phase diagram fit to the theory, and to what extent do the details matter? I ask because a key feature of the theoretical phase diagram is the negative slope of the lower arm of the binodal in the WT-Mut plane; however, there is no evidence supporting this in the limited number of experimental data points. Is this feature of the fitted phase diagram important for the model? If not, is a quantitative fit of the phase diagram necessary to provide a mechanistic explanation?"*

We consider a ternary Flory Huggins model to fit the phase diagram to obtain three interaction parameters (now also stressed in the revised main text) and the two RNA nanostars' molecular masses using a least square fit. We note that the molecular masses are consistent with estimates based on nucleotide mass counting (see discussion in SI).

Still, we obtained various fits with very similar errors. We decided to favor the one where the model's tie lines agree best with the experimental one. The reason for this choice is that the tie line slope, particularly relative to the fraction of mutant-to-wildtype DNA, is the key thermodynamic parameter for the spinodal instability. However, for the chosen fit, the composition of the dilute branch of the binodal is slightly outside the experimental data errors. The qualitative discrepancy noted by the reviewer is that the data would correspond to a positively sloped nano-star dilute branch, while the fitted model slope is negative. However, we note that the slope is almost vertical in both case, with approximately 100 in the experiment and about -100 in the theory. A better fit can only be obtained when introducing more parameter which

turns however to be unnecessary evidenced e.g. in the good agreement between theory and experiment on the condensate radius above which vacuole form.

Comment 6: *"I was surprised to find that the main text does not explicitly state whether the vacuole formation is a kinetic or thermodynamic effect. This distinction is important in general, but even more so in this study because the authors claim that the vacuoles are "stable" at several places in the manuscript. Moreover, it is not clear whether the theoretical model addresses the origin of this stability."*

We thank the reviewer for pointing this out and agree that we did not state this distinction clearly enough in the main text. In our system, vacuole formation is a kinetic, non-equilibrium effect driven by ongoing transcription and the resulting concentration gradients within droplets.

We also agree that the term "stable" can be misleading if interpreted as thermodynamic equilibrium. By "stable" we mean long-lived on experimental timescales: once formed, vacuoles persist for many hours to days. Importantly, we do not claim that vacuoles represent the equilibrium state; rather, we expect that given sufficiently long times when transcription has arrested due to nucleotide depletion, they will relax/disappear. This relaxation is extremely slow because the RNA nanostar-rich phase is highly viscous and exhibits very slow internal rearrangements, consistent with previous reports of limited recovery in FRAP experiments for related RNA condensates. We have revised the abstract and main text to make this point explicit and to define "stable" as long-lived rather than thermodynamically stable.

Regarding theory, our model addresses the kinetic origin of vacuole formation and its long lifetime: it predicts that transcription-driven production can push the local composition inside droplets into an unstable regime, leading to vacuole formation via a kinetic (spinodal) instability. The model does not contain any equilibrium thermodynamic stabilization mechanism for vacuoles; instead, it explains why vacuoles form under active driving and why their relaxation is expected to be slow in a highly viscous, dynamically arrested material. We have clarified this scope in the revised manuscript.

Comment 7: *"I expected to see more connections between the theory and experiments. As currently presented, the theory is mostly developed on the basis of one reverse-engineered experimental system. However, the predictions of this theory do not appear to be tested experimentally except for the comment that gradients were observed transiently in a prior figure panel. Can the theory explain all the experimental results described previously, including the various behaviors of the M, M+L, and M+S systems in Figure 2 and the various morphologies observed in Figure 3? In my view, making connections to all the experimental results is essential to establish the mechanistic interpretation convincingly. Addressing this shortcoming would also help to address comment (3) above."*

We thank the reviewer for this important comment and agree that the links between theory and the full set of experiments were not presented clearly enough. We have revised the manuscript to

better connect the modeling assumptions and predictions to the experimental results in Figs. 2–3, and we added an additional experimental test of the key theoretical prediction.

First, we clarify that the sequences used for the reverse-engineered mutant and the subsequent modeling are directly motivated by the gel-purified byproduct systems in Fig. 2 (M, M+S and M+L). In particular, the mutant class that we reconstruct and use for the phase diagram originates from the long byproduct band (L) that drives vacuole formation in these mixtures. We now state this explicitly in the Results and refer the reader to the corresponding sequencing/gel analysis.

Second, the main quantitative prediction of the model is that vacuole formation is facilitated by a finite mutant fraction in the transcribed pool and that, above a characteristic droplet size, droplets enter a regime where an internal demixing instability can be triggered during growth. Consistent with this prediction, we find experimentally that vacuoles appear only once droplets exceed a radius of approximately $\sim 20\,\mu\mathrm{m}$. We quantified this by estimating the droplet radius at which vacuolation occurs (termed "onset radius", Response Fig. 7). We have now extended this onset-radius analysis to droplets formed from Mut as well as to the vacuolated droplets generated from the Fig. 2 byproduct systems (e.g. M+L and M+S conditions, new Supplementary Fig. 19). We have added this comparison to the revised manuscript to make clear that the experimentally observed size threshold for vacuolation is in line with the theory, providing an additional independent link between the model and the broader experimental dataset.

We tested the mutant-fraction prediction experimentally by transcribing WT together with the reconstructed mutant template at the mutant fractions used in the theory. Consistent with the prediction, we observe vacuole formation at 11% mutant DNA (Response Fig. 8b,c, new data was added to the SI as Supplementary Fig. 20). This provides a direct experimental validation of the model for vacuole onset shown in Response Fig. 8a. Again, the estimated onset radius matches ($26\,\mu\mathrm{m}$ to $30\,\mu\mathrm{m}$) our theoretical predictions for 11% Mut DNA ($\sim 26\,\mu\mathrm{m}$) well.

We also note that obtaining a full phase diagram requires substantial experimental effort and is not feasible for every sequenced mutant. Nevertheless, the model makes a general, testable statement: mutants that sufficiently alter the relevant kinetic parameters (in particular internal mixing/transport relative to material input) can shift the system into a vacuole-forming regime. The additional mixing experiment shown in Response Fig. 8 supports this central prediction, and we now highlight this more explicitly in the revised manuscript.

Finally, regarding Fig. 3, we clarify the scope of the theory. Substitution mutations can plausibly alter the effective interaction parameters of the nanostars (for example by perturbing KL energies or by changing nanostar flexibility via reduced base-pairing in stem regions), and thus can promote entry into the vacuole-forming regime. However, the other morphologies observed at very high mutation rates (e.g. disrupted droplets/aggregates/network-like structures) are not intended to be explained by the present phase-diagram-based model. These regimes correspond to loss of droplet-forming function under an excessive mutational load, which we now describe more clearly in the revised manuscript.

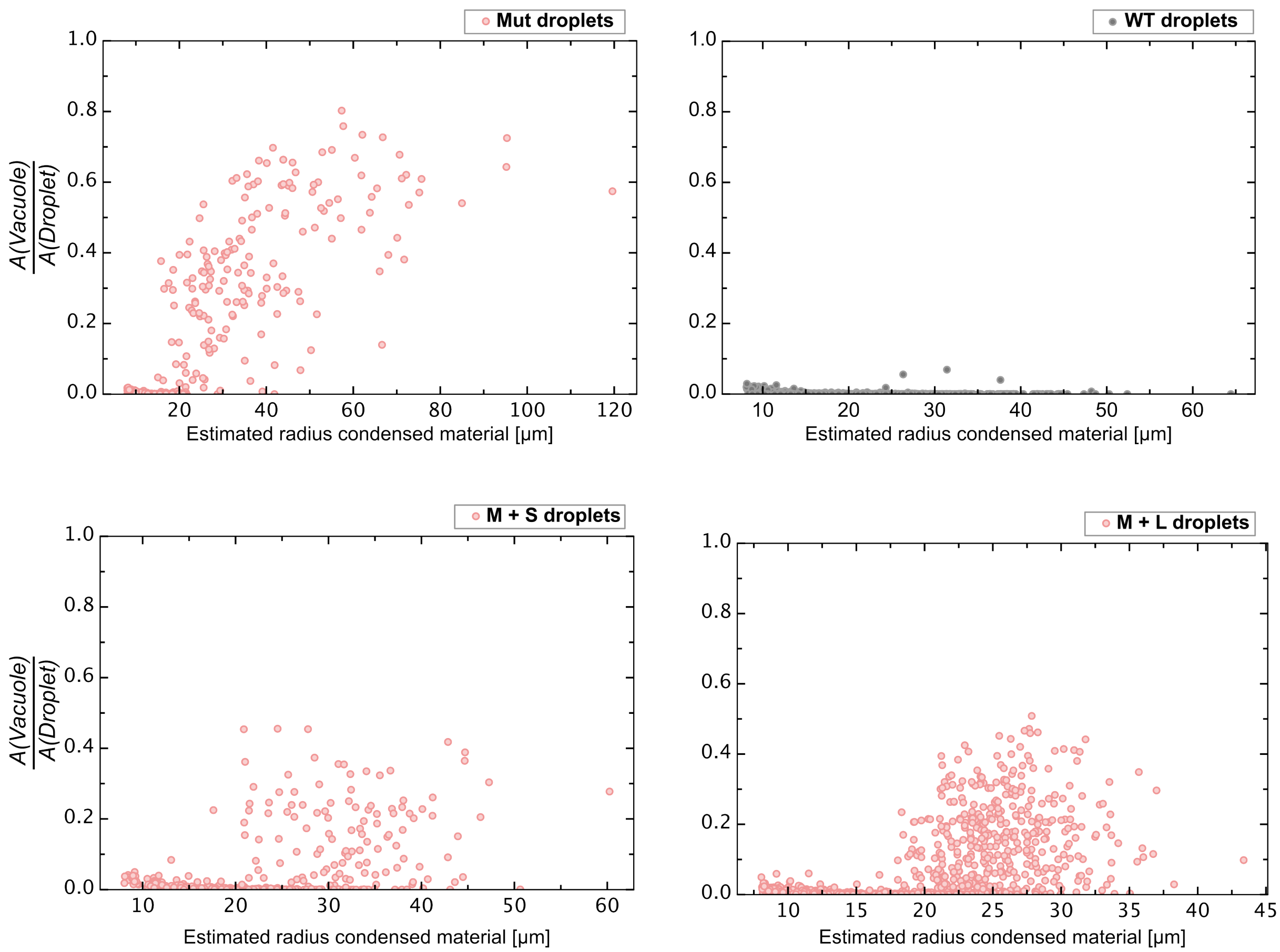

Response Fig. 7: **Estimated radius of condensed RNA at the onset of vacuolation.** We estimate the minimal droplet size at which vacuolation occurs, by measuring, in confocal images, the fluorescent area corresponding to the labelled RNA phase (see SI for more information). In accordance with the theory we find that only droplets above a certain size have vacuoles. For Mut droplets start forming vacuoles roughly around a radius of 15 μm to 20 μm. This is very close to the value predicted by our theoretical model for WT with 5% Mut-RNA, shown in Fig. 4c. No vacuoles are observed for WT in the droplet size range sampled.

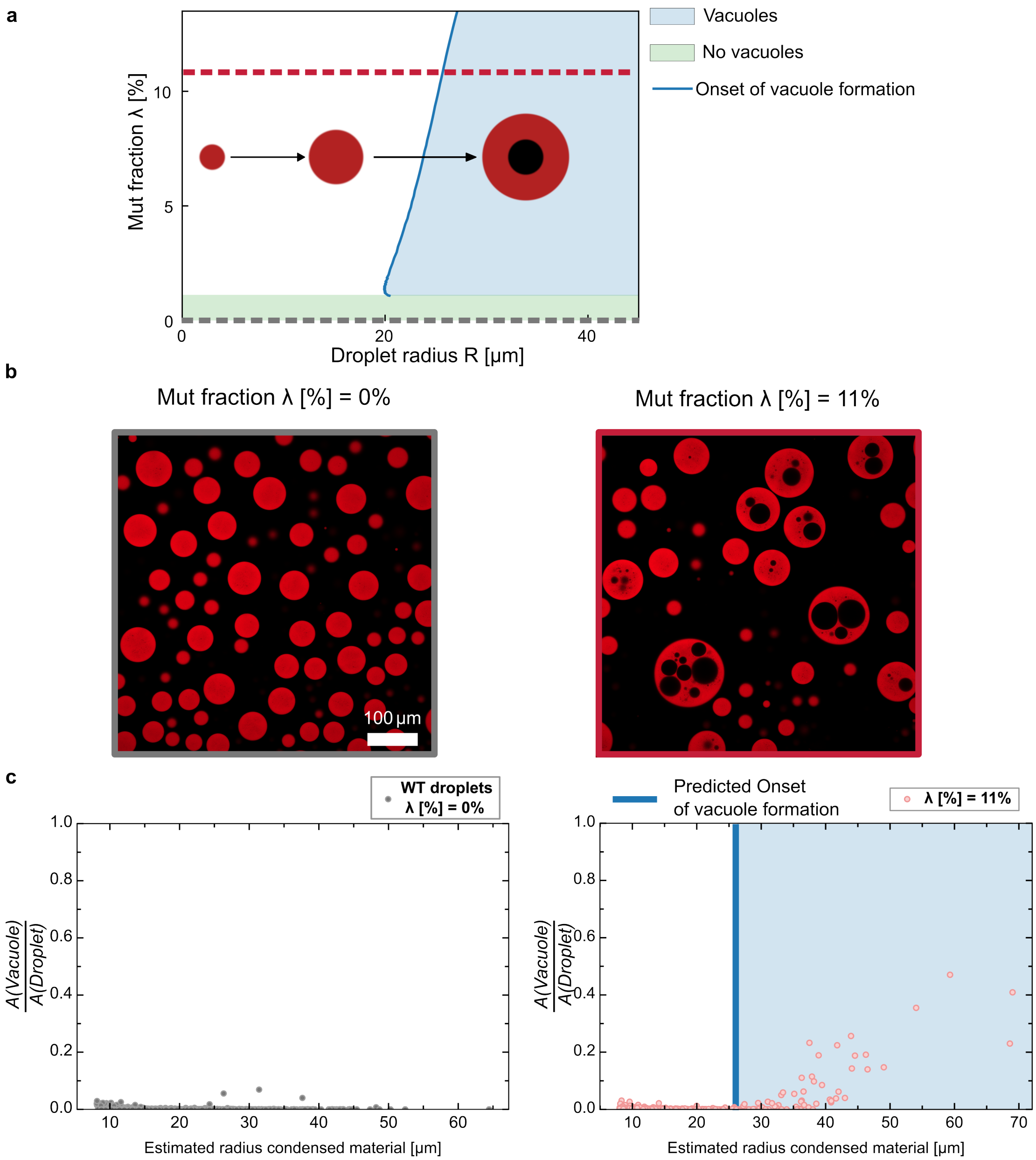

a
Vacuoles
No vacuoles
Onset of vacuole formation
Mut fraction λ [%]
Droplet radius R [μm]
b
Mut fraction λ [%] = 0%
Mut fraction λ [%] = 11%
100 μm
c
WT droplets
λ [%] = 0%
Predicted Onset
of vacuole formation
λ [%] = 11%
A(Vacuole)/A(Droplet)
Estimated radius condensed material [μm]

Response Fig. 8: **Experimental validation of the theoretical predictions for vacuole formation.** **a** Schematic of the model prediction: within our experimental conditions, vacuoles are expected only above a characteristic droplet size and only when a finite fraction of mutant species is present (blue region indicates the predicted vacuole-forming regime; dashed lines mark the mutant fraction used below). **b** Representative confocal images of droplets formed by transcription of WT only ($\lambda = 0\%$, left) and of WT with the reconstructed mutant template at $\lambda = 11\%$ (right). Scale bars, $100\,\mu\mathrm{m}$. **c** Quantification of the droplet radius at which vacuoles first appear ("onset radius") for WT droplets ($\lambda = 0\%$, left) and for WT with $\lambda = 11\%$ droplets (right). For $\lambda = 11\%$ the measured onset radius ($\sim 26\,\mu\mathrm{m}$ to $30\,\mu\mathrm{m}$) is in good agreement with the theoretical prediction ($\sim 26\,\mu\mathrm{m}$).

References

1. Jawerth, L., Fischer-Friedrich, E., Saha, S., Wang, J., Franzmann, T., Zhang, X., Sachweh, J., Ruer, M., Ijavi, M., Saha, S., Mahamid, J., Hyman, A.A., and Jülicher, F. (2020). Protein condensates as aging Maxwell fluids. Science *370*, 1317–1323. URL: `https://www.science.org/doi/10.1126/science.aaw4951`. doi: `10.1126/science.aaw4951`. Publisher: American Association for the Advancement of Science.

2. Erkamp, N.A., Verwiel, M.A.M., Qian, D., Sneideris, T., Spaepen, F.A., Weitz, D.A., van Hest, J.C.M., and Knowles, T.P.J. (2024). Biomolecular condensates with complex architectures via controlled nucleation. Nature Chemical Engineering *1*, 430–439. URL: `https://www.nature.com/articles/s44286-024-00077-7`. doi: `10.1038/s44286-024-00077-7`. Publisher: Nature Publishing Group.

3. Maass, C.C., Krüger, C., Herminghaus, S., and Bahr, C. (2016). Swimming Droplets. Annual Review of Condensed Matter Physics *7*, 171–193. URL: `https://www.annualreviews.org/content/journals/10.1146/annurev-conmatphys-031115-011517`. doi: `10.1146/annurev-conmatphys-031115-011517`. Publisher: Annual Reviews.

4. Lewontin, R.C. (1970). The Units of Selection. Annual Review of Ecology, Evolution, and Systematics *1*, 1–18. URL: `https://www.annualreviews.org/content/journals/10.1146/annurev.es.01.110170.000245`. doi: `10.1146/annurev.es.01.110170.000245`. Publisher: Annual Reviews.

5. Szathmáry, E., Santos, M., and Fernando, C. (2005). Evolutionary Potential and Requirements for Minimal Protocells. In P. Walde, ed. Prebiotic Chemistry pp. 167–211.. Berlin, Heidelberg: Springer. ISBN 978-3-540-31457-8 pp. 167–211. URL: `https://doi.org/10.1007/tcc001`. doi: `10.1007/tcc001`.

6. Adamala, K., and Szostak, J.W. (2013). Competition between model protocells driven by an encapsulated catalyst. Nature chemistry *5*, 495–501. URL: `https://pmc.ncbi.nlm.nih.gov/articles/PMC4041014/`. doi: `10.1038/nchem.1650`.

7. Matsuura, T., Yamaguchi, M., Ko-Mitamura, E.P., Shima, Y., Urabe, I., and Yomo, T. (2002). Importance of compartment formation for a self-encoding system. Proceedings of the National Academy of Sciences *99*, 7514–7517. URL: `https://www.pnas.org/doi/abs/10.1073/pnas.062710399`. doi: `10.1073/pnas.062710399`. Publisher: Proceedings of the National Academy of Sciences.

8. Matsumura, S., Kun, , Ryckelynck, M., Coldren, F., Szilágyi, A., Jossinet, F., Rick, C., Nghe, P., Szathmáry, E., and Griffiths, A.D. (2016). Transient compartmentalization of RNA replicators prevents extinction due to parasites. Science *354*, 1293–1296. URL: `https://`

www.science.org/doi/full/10.1126/science.aag1582. doi: 10.1126/science.aag1582. Publisher: American Association for the Advancement of Science.

9. Alshareedah, I., Moosa, M.M., Raju, M., Potoyan, D.A., and Banerjee, P.R. (2020). Phase transition of RNAprotein complexes into ordered hollow condensates. Proceedings of the National Academy of Sciences *117*, 15650–15658. URL: https://www.pnas.org/doi/10.1073/pnas.1922365117. doi: 10.1073/pnas.1922365117. Publisher: Proceedings of the National Academy of Sciences.

10. Fabrini, G., Farag, N., Nuccio, S.P., Li, S., Stewart, J.M., Tang, A.A., McCoy, R., Owens, R.M., Rothemund, P.W.K., Franco, E., Di Antonio, M., and Di Michele, L. (2024). Co-transcriptional production of programmable RNA condensates and synthetic organelles. Nature Nanotechnology *19*, 1665–1673. URL: https://www.nature.com/articles/s41565-024-01726-x. doi: 10.1038/s41565-024-01726-x. Publisher: Nature Publishing Group.

11. Stewart, J.M., Li, S., Tang, A.A., Klocke, M.A., Gobry, M.V., Fabrini, G., Di Michele, L., Rothemund, P.W.K., and Franco, E. (2024). Modular RNA motifs for orthogonal phase separated compartments. Nature Communications *15*, 6244. URL: https://www.nature.com/articles/s41467-024-50003-x. doi: 10.1038/s41467-024-50003-x. Publisher: Nature Publishing Group.

12. Brangwynne, C.P., Eckmann, C.R., Courson, D.S., Rybarska, A., Hoege, C., Gharakhani, J., Jülicher, F., and Hyman, A.A. (2009). Germline P Granules Are Liquid Droplets That Localize by Controlled Dissolution/Condensation. Science *324*, 1729–1732. URL: https://www.science.org/doi/full/10.1126/science.1172046. doi: 10.1126/science.1172046. Publisher: American Association for the Advancement of Science.

13. Fritsch, A.W., Diaz-Delgadillo, A.F., Adame-Arana, O., Hoege, C., Mittasch, M., Kreysing, M., Leaver, M., Hyman, A.A., Jülicher, F., and Weber, C.A. (2021). Local thermodynamics govern formation and dissolution of Caenorhabditis elegans P granule condensates. Proceedings of the National Academy of Sciences *118*, e2102772118. URL: https://www.pnas.org/doi/10.1073/pnas.2102772118. doi: 10.1073/pnas.2102772118. Publisher: Proceedings of the National Academy of Sciences.

14. Xie, M., Chen, W., Vonk-de Roy, M., and Walther, A. (2025). Constructing synthetic nuclear architectures via transcriptional condensates in a DNA protonucleus. Nature Communications *16*, 8254. URL: https://www.nature.com/articles/s41467-025-63445-8. doi: 10.1038/s41467-025-63445-8. Publisher: Nature Publishing Group.

15. Thomen, P., Lopez, P.J., Bockelmann, U., Guillerez, J., Dreyfus, M., and Heslot, F. (2008). T7 RNA Polymerase Studied by Force Measurements Varying Cofactor Concentration. Biophysical Journal *95*, 2423–2433. URL: https://www.cell.com/biophysj/abstract/S0006-3495(08)78388-7. doi: 10.1529/biophysj.107.125096. Publisher: Elsevier.

16. Zaccolo, M., Williams, D.M., Brown, D.M., and Gherardi, E. (1996). An approach to random mutagenesis of DNA using mixtures of triphosphate derivatives of nucleoside analogues. Journal of Molecular Biology *255*, 589–603. doi: 10.1006/jmbi.1996.0049.

17. Neylon, C. (2004). Chemical and biochemical strategies for the randomization of protein encoding DNA sequences: library construction methods for directed evolution. Nucleic Acids Research *32*, 1448–1459. URL: https://doi.org/10.1093/nar/gkh315. doi: 10.1093/nar/gkh315.

18. Piganeau, N., Thuillier, V., and Famulok, M. (2001). *In vitro* selection of allosteric ribozymes: theory and experimental validation1. Journal of Molecular Biology *312*, 1177–1190. URL: https://www.sciencedirect.com/science/article/pii/S0022283601949812. doi: 10.1006/jmbi.2001.4981.

Point-to-point response

Reviewer #1

"The authors have adequately addressed all the questions and comments raised during the review process. Based on these revisions, I am satisfied with the responses and support the manuscript for publication."

We thank Reviewer #1 for their positive assessment and for supporting the publication of our work in *Nature Chemical Engineering*.

Reviewer #2

"I appreciate the long and detailed rebuttal provided by the authors. Many aspects of the article have improved as a result; particularly the logic of certain experiments and the clarity of the text. Ultimately a fundamental experimental concern remains: In my initial review, I had asked (reviewer 2, comment 9) about the lifetime of the RNAP reaction, i.e. whether RNAP is still active and producing RNA throughout the 2-day time course of the typical experiment presented here. In response, the authors calculate the time to rNTP depletion. Here is their (supplementary) text describing that calculation:The experimental system is prepared with an rNTP concentration of 10 mM and a DNA concentration of 45 nM. The transcription rate for the considered RNA composed of 200 nucleotides is about 1/s. We can thus estimate that it takes about 60 h to deplete most of the rNTP nutrients. We conclude that when vacuoles form (around 6h, see Fig. 1 main text and Supplementary Video S2), the T7 polymerase is active at rNTP excess."

We thank Reviewer #2 for acknowledging that many aspects have been improved. We address the remaining concerns below.

Comment 1: "I believe the authors miscalculated the depletion timescale. In fact, I can recreate their 60 hour number if I assume the RNAP transcription rate is 1 nucleotide per second. However, the wording of their second sentence is more consistent with one transcript being produced per second, implying a transcription rate of 200 nt/s. In fact, 200 nt/s is consistent with published estimates of T7 RNAP speed at 37C. Thus I believe the actual time to depletion is around 200 times faster, i.e. around 18 minutes rather than 60 hours (=3600 minutes)."

We thank the reviewer for pointing out our calculation mistake, we indeed accidentally dropped the factor of 200. Please note that, in the previous submission, the theoretical computations correctly included the factor of 200; it was only dropped in the estimate. We find that the resulting change in the depletion time scale does not impact our findings on the mechanism of vacuole formation.

However, the point that the reviewer raised made us dig deeper. We suspect that the actual transcription rate in our system differs from the numbers in literature (200 nt/s) due to the different reaction conditions. We thus measured the transcription rate directly.

To this end, we consider the transcription of the WT2 nanostar (shown in Figure 3 in the main text) over time. We use the WT2 nanostar instead of the WT since the broccoli aptamer has more routinely been used to monitor transcription and *Fabrini et al.*[1] have reported unexpected behaviour of the malachite green dye in similar plate reader assays. We performed a plate reader assay measuring the emission of the DFHBI-1T dye.We find that transcription of WT2 follows a similar trend as the one described by *Fabrini et al.* for droplet B in their system[1]; see Response Fig. 1a below.

We fit this experimental data with the kinetic theory for transcription (see Eq. 23 and Eq. 24 in Supplementary Information) with a Hill coefficient of 1. The fit agrees well with the measured data (see Response Fig. 1b,c), yielding an effective RNA-transcription rate of $\tilde{k} \simeq 0.193\,\mathrm{s}^{-1}$. For WT2, the RNA is 257 nucleotides long, corresponding to a transcription rate of $\sim$ 49.7 nts/sec. This value is about a factor of four lower than the literature value at optimal bulk conditions[2,3].

The differences between our measured transcription rate and the ones reported in literature might occur as our reaction conditions are not optimized for fast transcription, as we are not using the proprietary buffer provided by NEB but a home made one, in an effort to better model our system. Also, the NEB kit states it should be used with 1 µg of DNA template. Whereas our reaction takes place with only 120 ng of DNA template. The NEB note also states that for an increased overall yield (for short transcripts <0.3kb) it is recommended to let the reaction run over night (see HiScribe® T7 High Yield RNA Synthesis Kit).

Thus, to reconsolidate these new findings, we updated our calculations with the actual experimental values. Details are given in a new SI Section 2.1 and 2.2.

Comment 2: ”These theoretical estimates correspond to the fastest possible depletion (e.g. making implicit assumptions that RNAP is saturating). Other effects could also serve to limit the reaction: e.g., multi-hour enzymatic reactions frequently become limited by the fragility of the enzyme itself. So it might be better to compare to published transcription experiments using T7 RNAP. From a quick literature search, I find several examples that report RNA production ceased after less than, or of order, 100 minutes in what appear to me to be roughly similar conditions, see e.g. DOI: 10.1038/s41598-024-59978-5, 10.1039/d5sm00868a, or the NEB technical note titled “Scaling of high-yield in vitro transcription reactions for linear increase in RNA production”. The longer experimental timescale vs. theory (100 min >18 min) could be because the published experiments typically start with more than 10 mM rNTP (and so lasts longer), or because of reduced enzyme activity as the reaction proceeds, or some other mechanism. I conclude that, after $\sim$ 2 hours (and possibly even earlier), the rate of transcript generation is very likely to be nearly zero. This is before even the 6 hour timepoint of vacuole appearance, and so casts serious doubts on the interpretations provided by the authors. I welcome any correction to the estimates I provide and apologize if I made an error; but, failing that, the only way I see to resolve this is

through direct measurement of RNA transcript production over time."

We thank the reviewer for this very thoughtful review. We indeed performed a direct measurement of RNA transcript production over time, as detailed in the response to Comment 1.

From these new experiments, we find that transcription reaches saturation after about 16 h, so after the onset of vacuole formation (Response Fig. 1a,b). Naturally, the reaction enters the nutrient limited regime earlier, namely when the production rate has decreased to half of its maximal value. In our case this happens after approximately 3.2 h (Response Fig. 1d). We now also provide a detailed discussion of the depletion time in the SI (see section 2.1 and 2.2).

Importantly, our proposed mechanism does not require transcription to remain at its maximal rate throughout the full 48-h observation period. The plate-reader measurements show approximately linear RNA accumulation during the first ($\sim$2 h), which overlaps with the earliest detectable vacuole formation in Mut droplets (Supplementary Video S2). During this initial phase, transcription drives condensate growth and establishes spatial composition gradients. Because molecular diffusion within the RNA-rich phase is slow, these gradients and the resulting structural rearrangements can persist and continue to evolve even as the transcription rate subsequently decreases.

Thus, the experimentally measured early transcription dynamics are sufficient to support the proposed mechanism, while sustained maximal transcription over the entire experimental time course is not required.

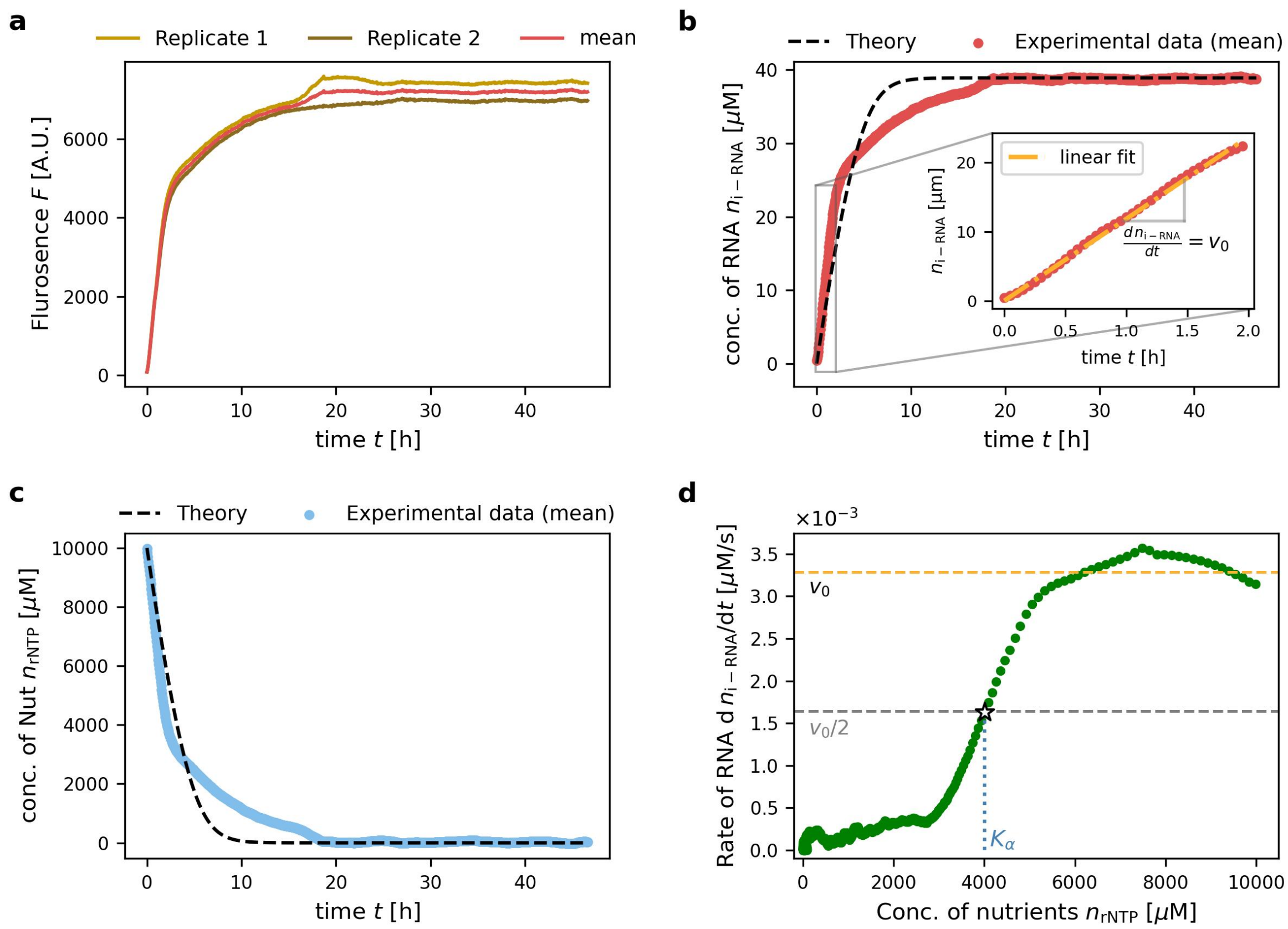

Response Fig. 1: **Measurement of transcription rate and threshold nutrient concentration** (a) Raw fluorescence traces from two transcription reactions and their mean, showing the accumulation of WT2 RNA over time. Fluorescence arises from DFHBI-1T binding to the Broccoli aptamer embedded within the WT2 nanostar, hence fluorescence increases with RNA concentration. The plateau fluorescence is used to calibrate the signal into RNA concentration by assuming complete consumption of the initially supplied nucleotides. (b) Time evolution of WT2-RNA concentration $n_{\text{i-RNA}}$ (where i=WT2): the theoretical prediction (black dashed line) closely follows the experimental data (red) across the full reaction. The inset highlights the early-time linear regime, where nucleotides are abundant and the production rate is approximately maximal. (c) Time-dependent nucleotide concentration inferred from the stoichiometric consumption of nucleotides (given in Eq. 27) during RNA production. (d) RNA production rate as a function of nucleotide concentration. The transcription rate approaches zero at low nutrient concentration and saturates at high nutrient concentration. We obtain threshold nutrient concentration K_α when production rate is $v_0/2$.

Comment 3: "Finally, I will mention that, in trying to carry out these calculations, I ran into various difficulties due to a lack of clarity in the text. For example, the T7 RNAP concentration is not clearly stated."

We thank the reviewer for pointing this out and agree that the description of polymerase input should be as transparent as possible. Unfortunately, to our knowledge, the HiScribe T7 transcription kit does not provide the T7 RNA polymerase concentration in molar units (or a calibrated enzyme-unit activity per volume that can be directly converted to a molar concentration). For this reason, we cannot report an absolute RNAP concentration in $\mathrm{mol\,L^{-1}}$.

To ensure reproducibility, we state explicitly in the Methods the exact volume of the kit-supplied T7 RNA polymerase stock added per reaction (and the final reaction volume), i.e. the polymerase input is fully specified in terms of the vendor reagent and its dilution.

We agree that reporting activity units or molar concentrations would be preferable when available, and in future work we will prioritise polymerase sources that provide calibrated enzymatic units and/or molar concentration to facilitate such calculations.

Comment 4: "Further, despite my comments in the initial review, and despite what the authors say in the rebuttal, the Fig 1 caption has not been revised to clarify that the time points in Fig 1 d/e are times since the start of transcription; nor has the blanket statement in the methods that "all images are taken after 48 h of transcription" been revised to account for the Figure 1 data."

We thank the reviewer for highlighting this and agree that the timing needed to be stated more explicitly. In the revised manuscript, we have now corrected both items. First, the caption of Fig. 1d,e specifies that the indicated time points represent the elapsed time since the start of transcription and that these panels show a time course. Second, we have revised the Methods to clarify that images used for endpoint quantification, including droplet-area measurements, are generally acquired approximately $48\,\mathrm{h}$ after transcription is initiated. The time-lapse datasets shown in Fig. 1d,e and quantified in Fig. 1f are exceptions, and their time stamps indicate the elapsed time since the start of transcription.

Comment 5: "A final minor note: I appreciate the addition of materials properties, but the wording describing it is poor: the capillary velocity is presented on the bottom of page 3 in a sentence reading "Consistent with the long-lived nature of the vacuoles, fusion events proceed on long timescales (capillary velocity $\sim 10^{-2}\mathrm{\mu m\,s^{-1}}$)...". The way this is written makes it sound like the vacuoles are fusing (whereas they are analyzing droplet fusion events). Further, this sentence doesn't spell out the actual logic the authors are proposing (which is that the material is highly viscous, which slows the dynamics of vacuole fusion)."

We thank the reviewer for this helpful remark and agree that our original wording was ambiguous. We have revised the sentence in the main text to make the intended logic explicit:

the low capillary velocity indicates viscous RNA-rich phase, which in turn supports our interpretation that internal rearrangements are slow and that vacuole–vacuole coalescence is rare on experimental timescales.

Reviewer #3

"The revised manuscript is substantially improved. The illustration and description of the vacuole-formation mechanism in the main text could be further improved, but the current version is acceptable."

We thank Reviewer #3 for their careful assessment and positive evaluation.

References

1. Fabrini, G., Farag, N., Nuccio, S.P., Li, S., Stewart, J.M., Tang, A.A., McCoy, R., Owens, R.M., Rothemund, P.W.K., Franco, E., Di Antonio, M., and Di Michele, L. (2024). Co-transcriptional production of programmable RNA condensates and synthetic organelles. Nature Nanotechnology *19*, 1665–1673. URL: `https://www.nature.com/articles/s41565-024-01726-x`. doi: `10.1038/s41565-024-01726-x`. Publisher: Nature Publishing Group.

2. Pavco, P.A., and Steege, D.A. (1991). Characterization of elongating t7 and sp6 rna polymerases and their response to a roadblock generated by a site-specific dna binding protein. Nucleic acids research *19*, 4639–4646.

3. Sastry, S.S., and Ross, B.M. (1997). Nuclease activity of t7 rna polymerase and the heterogeneity of transcription elongation complexes. Journal of Biological Chemistry *272*, 8644–8652.

Point-to-point response

Manuscript NATCHEMENG-25051060C, *"Genetic encoding and mutagenesis of RNA droplet phenotypes"*.

We thank the Editor and the Reviewers for their continued engagement with our manuscript. In this round the revised manuscript was seen by Reviewer #2, whose comment is reproduced and addressed below. All editorial and formatting requirements raised in the Author Checklist are addressed in the completed checklist submitted alongside this response.

Reviewer #2

"I appreciate the careful work that the authors have carried out to illuminate the transcription dynamics. I have no further comments, and feel that the is acceptable as it stands."

We thank Reviewer #2 for this positive assessment and for supporting publication of our work in *Nature Chemical Engineering*.

We are also grateful for the reviewer's persistence on the question of transcription lifetime in the previous round. We believe the manuscript is stronger for this addition.

No further changes have been made in response to the reviewers.

Changes made in this round

For completeness, the revisions made since the previous version are editorial and formatting changes requested in the Author Checklist rather than responses to reviewer comments. They are documented in full in the completed Author Checklist, and comprise:

- Restructured end matter, with *Data availability* and *Code availability* as separate sections.
- Figure and Supplementary figure legends revised to state exact sample sizes, exact P values, the statistical test used, the definition of error bars and box-plot elements and the number of independent repeats.
- Supplementary Figs. 5 and 13 re-plotted with individual data points overlaid on the bar charts.
- Uncropped scans of all gels shown in Supplementary Figures added at the end of the Supplementary Information; the uncropped scan of the gel in Fig. 2a supplied as Source Data.
- Source Data files supplied for all graphs in Figs. in the main text.

- Oligonucleotide sequences additionally supplied in machine-readable form as Supplementary Data 1, referenced from the Methods.
- A graphical abstract and a completed Reporting Summary supplied.
- All Editorial comments are also addressed.
