# Supplementary material for: Genetic encoding and mutagenesis of RNA droplet phenotypes
Source: Nat Chem Eng. 2026 Oct 7;3(10):573–83. doi: 10.1038/s44286-026-00454-4 (PMC13680278; doi:10.1038/s44286-026-00454-4)

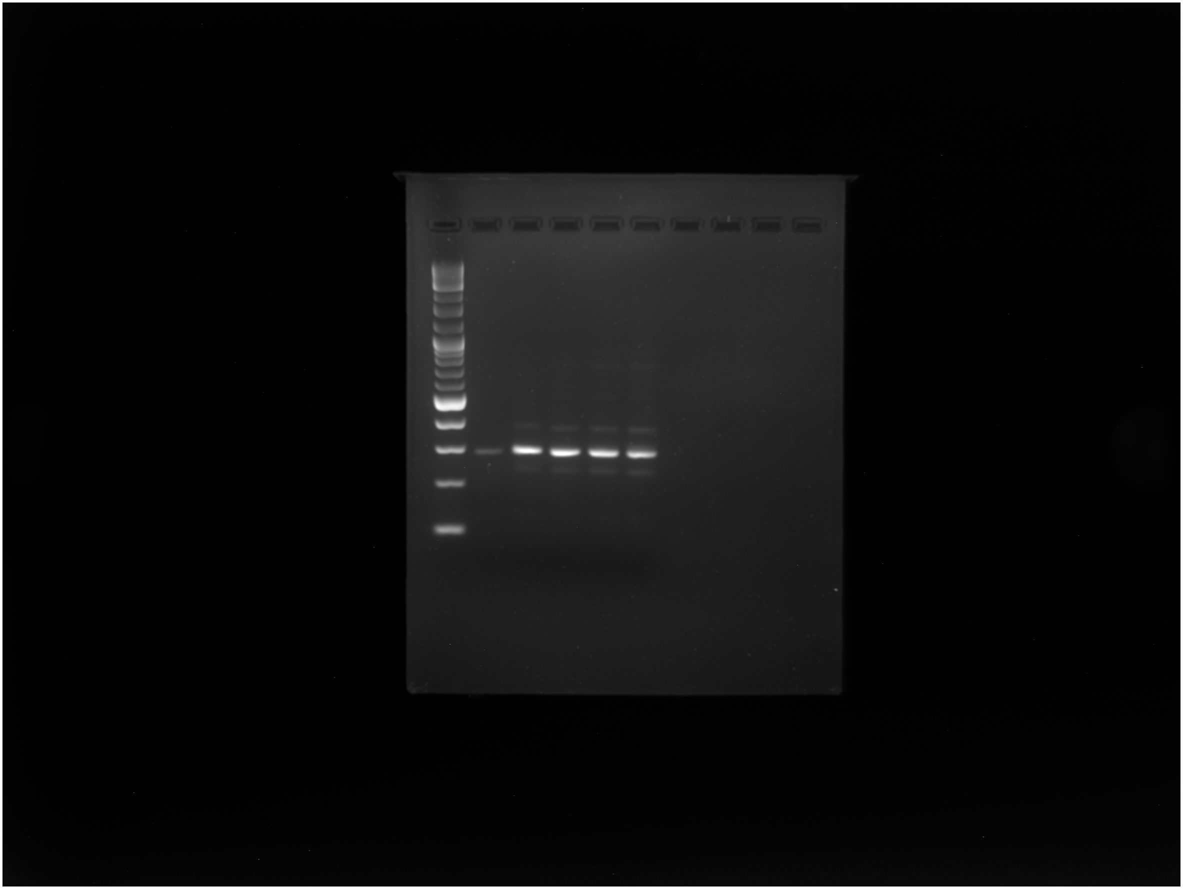

Supplement: Supplementary file 4 [file 44286_2026_454_MOESM12_ESM.zip › FIgure2_gel.pdf]
